# Hybrid Nanofibers for Multimodal Accelerated Wound Healing

**DOI:** 10.1002/adhm.202504029

**Published:** 2026-01-28

**Authors:** Viraj P. Nirwan, Bence Bajusz, Norbert Fabók, Marina Rudan Dimlic, Jelena Budimir, Tshepang Mqatywa, Miklós Gyöngy, Márton Ferencz, Dorottya Kocsis, Olexandr Bondarenko, Mariia Rolduhina, Milena Lengyel, Istvan Antal, Rebecca Hengsbach, Franciska Erdő, Amir Fahmi

**Affiliations:** ^1^ Faculty of Technology and Bionics Rhine‐Waal University of Applied Sciences Marie‐Curie‐Straße 1 47533 Kleve Germany; ^2^ Faculty of Information Technology and Bionics Pázmány Péter Catholic University Budapest Hungary; ^3^ Dermus Budapest Hungary; ^4^ MedILS University of Split Split Croatia; ^5^ Dnipro State Medical University Dnipro Ukraine; ^6^ Department of Pharmaceutics, Faculty of Pharmaceutical Sciences Semmelweis University Budapest Hungary

**Keywords:** accelerated wound healing, biocompatible polymers, electrospinning, functionalized hybrid nanofibers, in vivo analysis

## Abstract

Wound healing is a complex physiological process that demands multifunctional therapeutic approaches to ensure effective recovery. This study presents a straightforward approach using blend electrospinning to produce multimodal hybrid nanomaterials that accelerate the wound healing process. Poly(L‐lactide‐co‐ε‐caprolactone) (PLCL), cellulose acetate (CA), and polyethylene oxide (PEO) were utilized as biodegradable, compatible, and compliant polymers for generating nanofibers. Hybrid nanofibers functionalized with dexamethasone, ascorbic acid, and hyperbranched polymers introduce anti‐inflammatory, regenerative, and antimicrobial properties. Pristine nanofibers with diameters of 0.818 ± 0.028 and 0.845 ± 0.039 µm were generated, while drug‐loaded fibers with average diameters of 1.075 ± 0.055 and 1.235 ± 0.075 µm were obtained. The fibers demonstrated a porosity ranging from 72 % to 86 %. Further, attenuated total reflectance Fourier transform infrared spectroscopy (ATR‐FTIR), thermogravimetric analysis (TGA), and contact angle, as well as zeta potential measurements, highlight the physicochemical properties of the fibers. In vivo studies of the nanofibers demonstrated that by day 11, there was a significant acceleration in wound healing. A remarkable acceleration was observed in cell proliferation, granulation, and remodeling phases. The findings emphasize the potential of multimodal hybrid nanofibers as advanced wound dressings and the importance of integrative strategies in wound care.

## Graphical abstract

Fabrication of wound healing scaffolds based on biocompatible nanofibers. Nanofibers offering high surface area, flexibility, and biocompatibility significantly improved the healing outcome in vivo. Histological, immunological, and anti‐inflammatory markers are noticeably better in treated wounds. The scalable approach addresses a critical need for a holistic methodology in regenerative medicine.

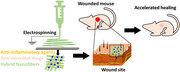

## 1 Introduction

The skin is the primary barrier and the first line of defense against pathogens and harsh environments (temperature, humidity, light, etc.). Additionally, it regulates and internalizes organs and complicated biochemistry [1]. Therefore, any disruption to the continuation of the protective skin barrier can expose the organism to a range of consequential threats, impairing its survivability. The clinical, economic, and societal importance of developing effective wound healing bandages is underlined by the vast number of studies that have been performed since the turn of the century. It is a complex process that involves the cooperation of multiple hormones, proteins, and biomolecules working in tandem across various phases of wound healing. Effective wound healing takes place in four stages, which are generally overlapping and vary in duration based on the site and severity of the wound [2, 3]. The initial phase of wound healing is hemostasis, followed by inflammation, which leads to proliferation and, finally, remodeling. Furthermore, the wound itself can be categorized based on the severity, site, and infection state. Sethuram et al. provide an exhaustive list of the subcategories of wounds, including “closed wounds, acute or chronic wounds, clean or contaminated wounds, internal or external wounds, non‐penetrating wounds such as abrasions, lacerations, bruises, concussions, and penetrating wounds such as stab wounds, cuts, surgical wounds, etc” [4]. Among them, infected chronic wounds lead to high mortality rates and cause an enormous economic burden on the healthcare infrastructure. Traditional wound healing strategies often follow a 1D approach using textile bandages, grafting, antibiotic injections, or extensive surgeries based on the severity. And their combination with drugs can lead to bandages having poor gas exchange ability and moisturizing capability [5, 6]. Furthermore, the efficacy of the drugs decreases due to insufficient release or instability [7]. Similarly, grafting and surgeries are extensive procedures with associated costs and often require repetition due to complications [8]. Largely, these strategies fail to consider the internal mechanism of wound healing, isolating the cell mechanisms, including hormone exchanges taking place at the wound site. Ideally, a wound dressing should possess good biocompatibility, degradability, breathability, water absorption, dust resistance, and antibacterial properties [9]. Here, advancements in nanomaterials, specifically drug‐loaded nanofibers, have shown enormous potential [10, 11]. Their intrinsic morphology, physicochemical properties, and antimicrobial effect have been shown to support and accelerate wound healing at various stages due to their responsive nature [12, 13]. Electrospinning generated nanofibers provide numerous advantages, such as the utilization and combination of a diverse range of biopolymers (silk, collagen, gelatin, chitosan, poly(lactic acid), poly(vinyl alcohol), poly(ε‐caprolactone), and (PLCL)) [14, 15, 16, 17, 18, 19, 20, 21], antimicrobial metal nanoparticles (zinc oxide nanoparticles, silver nanoparticles, and silver oxide) and biomolecules [22, 23, 24]. Additionally, allowing the tailoring of morphology, mechanical, chemical, and electrical properties based on the applications [9, 25, 26]. Resulting in multifunctional hybrid nanofibers with tunable characteristics using reproducible processes suitable for scaling up and large‐scale transformation.

A wide selection of natural and synthetic polymers has been used to generate nanofibers for inducing and promoting cell growth. Classically, the nanofibers are functionalized using antimicrobial agents distributed across a polymer matrix [27, 28, 29]. Often, hybrid nanofibers containing anti‐inflammatory biomolecules [30, 31] and nanoparticles have been used as vehicles for drug delivery for targeted wound healing [2, 32, 33]. The comprehensive and complicated nature of the wound healing mechanism requires a holistic approach. The material should be multifunctional to support the overlapped wound healing phases as they manifest while preventing potential infections. It should provide a characteristic atmosphere to support cell growth and tissue formation. Consequently, possessing the support structure, antimicrobial/anti‐inflammatory potential, and growth factor side by side can increase their accessibility and availability. PLCL, CA, and PEO are biodegradable and biocompatible polymers that have been used to generate nanofibers and tissue regeneration. The scaffolds generated using these polymers as a blend or in isolation have shown good biocompatibility in vitro [34, 35, 36, 37, 38]. So far, dexamethasone has been quite effective as an anti‐inflammatory agent [39, 40], whereas ascorbic acid has been shown to improve wound healing outcomes by supporting all phases [41]. Additionally, hyperbranched polymers have shown effective antibacterial properties with minimal effect on cell growth and compatibility [42, 43, 44]. Hence, multiple formulations were developed in this study using these polymers and functional moieties, and were electrospun to generate multifunctional, responsive nanofiber scaffolds for accelerated wound healing. Based on their intrinsic interactions with water, PLCL and PEO were used as a matrix to obtain nanofibers possessing hydrophobic and hydrophilic properties. CA was used as a co‐spinning agent for nanofibers to provide structural support and improve electrospinning. While dexamethasone, ascorbic acid, and hyperbranched polymers were added as anti‐inflammatory, antimicrobial, and supplement agents to support nanofibers. Fabricated nanofibers were characterized using physicochemical analyses such as scanning electron microscopy (SEM), contact angle, zeta potential, and porosity measurements, thermogravimetric analysis (TGA), and attenuated total reflection Fourier‐transform infrared spectroscopy (ATR‐FTIR). Thereafter, the effectiveness of the nanofiber patches was studied in vivo, and finally, histological evaluations were performed. This study underscores that improved and effective wound healing outcomes require comprehensive and holistic strategies.

## 2 Materials and Methods

### 2.1 Materials

Poly(l‐lactide‐co‐ε‐caprolactone) (70:30) (PURASORB PLC 7015) (PLCL) copolymer was bought from Corbion N.V., Netherlands. Formic acid, dichloromethane (DCM), and trifluoroacetic acid (TFA) from Carl Roth, GmbH Karlsruhe, Germany, were used as solvents. Dexamethasone was purchased from Molekula GmbH. Hyperbranched bis‐MPA PEG 10k Generation 4 hyperbranched polymer was provided by Polymer Factory Sweden AB. Ascorbic acid was purchased from VWR Chemicals, poly (ethylene glycol) 300 kg mol^−1^ from Sigma–Aldrich, Merck KGaA, Darmstadt, Germany, and cellulose acetate 100 kg mol^−1^ from Thermo Scientific Chemicals. Cosmos cover spray (Paul Hartmann AG, Heidenheim, Germany), isoflurane (Baxter, Deerfield, IL, USA).

### 2.2 Fabrication of Wound Dressings

Fabrication of nanofibers was performed on a climate‐controlled electrospinning device with a flow‐controlled pump, a positive voltage generator up to 25 kV, and a negative voltage generator to −4 kV. The fibers were collected on a rotating drum collector rotating at 200 rpm and covered with parchment paper for easy removal of fibers. The nanofibers were generated using the compositions and parameters given in Table 1.

**TABLE 1:** A brief description of the compositions used for the fabrication of nanofibers, including the electrospinning parameters implemented to obtain nanofibers.

Sample name	Polymer 1	Polymer 2	Electrospinning parameters
CA‐PLCL pristine	CA 1.36 g in 8 mL TFA	PLCL 0.46 g in 2 mL DCM	14 kV/−4 kV; 0.7 mL h^−1;^ 35 °C, 30 %
CA‐PLCL loaded	CA 1.36 g in 8 mL TFA + 100 mg dexamethasone, + 100 mg hyperbranched polymer	PLCL 0.46 g in 2 mL DCM + 100 mg ascorbic acid	14 kV /−4 kV; 0.7 mL h^−1;^ 35 °C, 30 %
PEO‐PLCL pristine	PEO 0.6 g in 9 mL formic acid	PLCL 0.46 g in 3 mL DCM	17 kV/−4 kV; 0.7 mL h^−1;^ 20 °C, 30 %
PEO‐PLCL loaded	PEO 0.6 g in 9 mL formic acid + 100 mg dexamethasone + 100 mg hyperbranched polymer	PLCL 0.46 g in 3 mL DCM + 100 mg ascorbic acid	17 kV/−4 kV; 0.7 mL h^−1;^ 20 °C, 30 %

### 2.3 Scanning Electron Microscopy of Nanofibers

The wound dressing polymers (CA‐PLCL pristine, CA‐PLCL loaded, PEO‐PLCL pristine, PEO‐PLCL loaded) were analyzed using a Hitachi TM4000Plus II SEM using 15 keV acceleration voltage in secondary electron mode (SE) at an 11 mm working distance.

### 2.4 Water Uptake Capacity Test

The water absorption capacity of pristine and drug‐loaded electrospun nanofiber matrices was determined following established protocols with minor modifications [45, 46, 47]. Rectangular samples (10 × 10 mm) were cut and weighed to determine their initial dry mass (W_0_) using an analytical balance (Ohaus, Pioneer, Merck, Budapest, Hungary). Each sample was immersed in 2 mL of MilliQ water and incubated at room temperature for 8 or 24 h.

After every interval, samples were removed, gently blotted to eliminate superficial water, and weighed to obtain the swollen mass (Wt_n_). Water uptake was calculated using Equation (1):

(1) $$\mathrm{Water}\;\mathrm{uptake}\left(\%\right)=\left(\left(\left({\mathrm{Wt}}_{n}\right)/W_{o}\right)-1\right)\times 100$$

All experiments were performed in triplicate (*n* = 3), and results are reported as mean ± standard deviation (SD).

### 2.5 Layer Thickness Determination

The thickness of the nanofiber matrices was determined using a digital caliper (Mitutoyo micrometer, Kvalifix Kft, Budapest, Hungary). Thickness measurements were performed on single‐, double‐, and quadruple‐layer samples, and the average single‐layer thickness was calculated. In all cases, the basal paper carrier layer was included during measurement. The procedure followed previously reported methods [45, 46]. Results are expressed as mean ± SD.

### 2.6 Mechanical Characterisation: Tensile Test

Tensile strength measurements were performed by Zwicki‐Line Z005 table top universal testing machine (ZwickRoell GmbH & Co. KG, Ulm, Germany) equipped with a 5 kN load cell. The samples (length x width: 20 × 10 mm) were kept at room temperature in closed containers for 24 h prior to the measurements, then fixed in a pincer grip and tested at a crosshead speed of 0.1 mm/min. The initial distance between the grips was set to 10 mm. The stress–strain curves were recorded using TestXpert II software (ZwickRoell GmbH & Co. KG) until sample rupture. Young's modulus was calculated from the slope of the linear elastic region in the stress–strain curve, where stress (σ) is proportional to strain (ε) per Hooke's law:

(2) $$E=\frac{\sigma }{\varepsilon }$$

Results of tensile strength measurement of nanofiber wound dressings (*n* = 5) are shown in Figure S3A,B.

### 2.7 Contact Angle, Zeta Potential, and Porosity Measurements

The wettability of the nanofibers was examined using a Goniometer OCA 35 with tilting unit TBU 90E´DataPhysics Instruments, Germany, attached with a camera iDS UI‐3360CP‐M‐GL R2. Water droplets (5 µL) were deposited on the samples and monitored for up to 120 s. Zeta potential measurements, including conductivity and electrophoretic mobility of the nanofibers dispersed in ethanol, were conducted on a Malvern Zetasizer NanoS equipped with a HeNe laser (*λ* = 633 nm) with *P* = 4 mW. Each measurement was done three times, with 20 repetitions each, after an equilibration time of 120 s using DTS1070 cuvettes.

The porosity of generated fibers was measured using the liquid pycnometer method [48, 49]. Ethanol was used to impregnate scaffolds, and a vacuum was applied to ensure the removal of trapped air within the fibers. Initially, the weight of the pycnometer filled with ethanol (W_1_) and the weight of dry fibers (W_s_) were measured. Thereafter, the fibers were immersed in a pycnometer, and a stopper was inserted. The pycnometer was kept under a vacuum to release the trapped air. The volume of displaced ethanol was replenished as the weight was measured (W_2_). Finally, the weight of the pycnometer was measured (W_3_) after removing the fibers. Total porosity of the fibers was calculated using the following equation:

(3) $$Porosity\left(\%\right)=\frac{W_{2}-W_{3}-W_{s}}{W_{1}-W_{3}}\times 100$$

### 2.8 Thermal Analysis and Attenuated Total Reflection (ATR) Fourier‐Transform Infrared Spectroscopy (FTIR)

The effect of the addition of the various drugs and biomolecules on the thermal stability of the nanofibers, including the weight loss behavior, was analyzed using thermogravimetric analysis (TGA). To perform TGA (Perkin Elmer‐ TGA 4000), fiber samples were cut, and ∼10 mg of fibers were taken in a ceramic cuvette, which was subjected to a temperature program running from 30 °C to 700 °C at 10°C min^−1^. The spectroscopy analysis was performed here to characterize the change in composition and related changes in vibration frequencies, which might have occurred due to the addition of biomolecules. The PerkinElmer Spectrum 2000 spectrometer with the ATR assembly was used to perform FTIR spectroscopy at a scanning resolution of 2 cm^−1^. Electrospun fibers were pressed simply against the crystal on the top plate for analysis, and the spectra were recorded. The data analysis and graphs for TGA and FTIR were prepared using OriginLab.

### 2.9 In Vitro Cytotoxicity

The in vitro cytotoxicity of the nanofiber bandages was evaluated using the MTT (3‐(4,5‐dimethylthiazol‐2‐yl)‐2,5 diphenyl tetrazolium bromide) assay on human immortalized keratinocyte (HaCaT) and primary dermal fibroblast (FIB) cell lines. Cells were seeded at a confluency of 35 000 and 15 000 cells per well of a 96‐well plate for HaCaT and FIB, respectively. After 24 h, metabolic activity was determined following the manufacturer's instructions. Briefly, a sterile filtered MTT stock solution (5 mg mL^−1^ in phosphate buffer saline, PBS, pH 7.4) was added to treated cells to achieve a final concentration of 0.5 mg mL^−1^. After 2 h of incubation at 37 °C and 5 % CO_2_, the unreacted dye was removed, and the resulting formazan crystals were dissolved in DMSO. Absorbance was measured at 595 nm using a multimode plate reader (EnSightTM PerkinElmer, USA). Cell viability was calculated as a percentage relative to untreated controls.

To prepare nanofiber solutions, the electrospun wound dressings were flash‐frozen in liquid nitrogen, ground into powder, and dissolved in 25 % DMSO (v/v) in water. The cells were treated with the nanofiber supernatant (0.25–4.0 mg mL^−1^). Additionally, cytotoxicity of the individual active components, dexamethasone and ascorbic acid, was evaluated independently in both cell types.

### 2.10 Animals

The hairless Crl:SKH1‐Hrhr (referred to as SKH1) mouse strain on a BALB/c genetic background was purchased from Charles River Laboratories (Germany) [50]. Animals were bred and housed individually (Techniplast, Buguggiate, Italy) in a conventional animal facility. Animals were fed standard chow ad libitum and had unlimited access to water. Age‐ and gender‐matched animals were used for the experiments. All experiments were approved by the Animal Experimentation Review Board of Semmelweis University and the Government Office for Pest County (Hungary) (Ethical approval number: PE/EA/00353‐2/2025).

### 2.11 In Vivo Wound Healing in Mice

In total, 25 hairless SKH1 mice of both genders were used for the experiments. Animal care and experimental protocol were approved by the Animal Experimentation Review Board of Semmelweis University and the Government Office for Pest County (Hungary).

Before conducting the experimental protocol, all mice were housed in a conventional area to undergo an acclimatization phase lasting one week to reduce stress. On the day of surgical wounding, the animals were randomly divided into 5 groups (5 animals/group). Under inhalation anesthesia (2 % isoflurane in oxygen), a single round full thickness skin wound was created on the back of each animal by a sterile, disposable 4 mm diameter skin biopsy punch (Figure S1). Animals were assigned to the following groups: (1) wounded and using Cosmos cover spray, (2) wounded and covering by PEO‐PLCL nanofiber wound dressing and Cosmos cover spray, (3) wounded and covering by PEO‐PLCL nanofiber loaded with dexamethasone and ascorbic acid wound dressing and Cosmos cover spray, (4) wounded and covering by CA‐PLCL nanofiber wound dressing and Cosmos cover spray, (5) wounded and covering by CA‐PLCL nanofiber loaded with dexamethasone and ascorbic acid wound dressing and Cosmos cover spray. All experimental groups were treated with a secondary dressing (transparent dressing—Cosmos cover spray) that serves to keep scaffolds in place and to maintain wound sterility.

According to the experimental design, the animals were weighed on the first and the last day, and the wounds were photographed every other day. Simultaneously, the diameters of the wounds were determined digitally on the photos. This procedure was performed under light isoflurane anesthesia. Fresh nanofiber dressings were placed on the wounds on the first and eighth days. On day 15, the wound dressings were removed, the animals were sacrificed by CO_2_ inhalation overdose followed by cervical dislocation, and the wounds were photographed before tissue excision. Tissues taken from the wounded and normal areas were fixed in 4 % neutral buffered paraformaldehyde for histological analysis.

### 2.12 Morphological Evaluation of Wound Closure

The healing area was measured according to the previously described protocol on a schedule, as seen in Table S1 [51]. At 1‐3‐5‐7‐9‐11‐13‐15 days, wounds were photographed, and the wound area was measured by Image J software (v1.54m, National Institutes of Health, US). Wound areas and diameters were expressed as a percentage of area/diameter at day 1:

(4) $$\begin{array}{ccc} & & \mathrm{woun}\mathrm{dare}\mathrm{aord}\mathrm{iame}\mathrm{tera}\mathrm{tdayX}(\%)\\ & & \;=\frac{\mathrm{wound}\;\mathrm{area}\;\mathrm{or}\;\mathrm{diam}\mathrm{eter}\;\mathrm{at}\;\mathrm{day}\;X}{\mathrm{wound}\;\mathrm{area}\;\mathrm{or}\;\mathrm{diam}\mathrm{eter}\;\mathrm{at}\;\mathrm{day}\;1}\times 100 \end{array}$$

### 2.13 High‐Frequency Ultrasound and Optical Imaging

The aim of taking measurements with the Dermus SkinScanner was to perform measurements of wound size and depth with time (as an outcome measure of healing), both using its “dermoscopy” (optical) and high‐frequency ultrasound mode. The Dermus SkinScanner employs a dermoscopy‐assisted high‐frequency ultrasound device primarily used for the study of skin, with applications in clinical imaging. It has an ultrasound frequency of 20–40 MHz, an optical field of view of 12 × 12 mm, and an ultrasound field of view of 10 mm depth x 12 mm lateral. We focused on the optical and ultrasound monitoring of wound healing. The aim was to compare day‐to‐day changes during a 15‐day period using our dermoscopy‐assisted high‐frequency ultrasound (derma‐HFUS) device. This study concludes that the derma‐HFUS device was effective in monitoring the healing process both on the surface and the depth of the mouse skin.

### 2.14 Image Analysis

The image analysis software ImageJ was used for two purposes. First, the diameters of nanofibers were analyzed for distribution frequency (Figures  2 and 3). Second, the wound diameters and areas were determined digitally (Figure 9A,B).

The fiber diameters were measured on the SEM images using ImageJ software (v1.54m, National Institutes of Health, US). Following this, the images underwent a series of adjustments to enhance the brightness and contrast. Subsequently, the Otsu algorithm was utilized for automatic threshold determination. The images were then divided into four equal segments. The diameters of 25 nanofibers were then measured along the left and bottom edges of the segmented images.

The ImageJ software was used to find the area and diameter of the wounds on the photos as well. To measure their area, we carefully traced the outlines of the wounds with one of the software's built‐in features. This resulted in a closed polygon given in pixels. For the diameter, we used another feature, drawing a straight line from one side, across the center of the wound, and to the opposite side, then taking the length of the drawn line. This was repeated three more times, each time rotating the line by 45°. After that, the average of the four lines was calculated. These processes were necessary due to the wounds being either circular or oval in shape.

### 2.15 Histological Evaluation of Wounded Tissues

Tissue infiltration was performed using a Microm STP‐120 tissue processor (Thermo Fisher Scientific, Germany). After formalin fixation, samples were dehydrated in a graded series of isopropanol (70 %, 80 %, 95 %, and three changes of 100 %, each for 90 min), cleared in xylene, and infiltrated with molten paraffin in two changes, each for 120 min. The paraffin‐embedded tissues were then embedded into paraffin blocks using a HistoStar embedding station (Thermo Fisher Scientific, USA).

Serial sections, no more than 4 µm thick, were obtained from the blocks using a Thermo HM 355S microtome (Thermo Fisher Scientific, Germany). Sections of each tissue sample (4 µm) were used for routine histological staining with hematoxylin and eosin [52], Goldner's trichrome [53], and subsequent immunohistochemical staining.

Microscopic examination of histological sections was performed using an Axio Imager 2 microscope (Zeiss, Germany) at x100, x200, and x400 magnifications. Tissue inflammation was evaluated using semiquantitative scoring system for the polymorphonuclear cells, macrophages, and lymphocytes [54] as follows: (G0) absent; (G1) mild – up to 5 inflammatory cells per x400 field of view (FOV); (G2) moderate—5–10 inflammatory cells/FOV; and (G3) severe—heavy inflammatory infiltrate.

### 2.16 Immunohistochemistry of Wounded Tissues

Sections of 4 µm thickness were mounted on Superfrost adhesive slides (Thermo, Germany), deparaffinized with xylene, and rehydrated. Endogenous peroxidase activity was blocked using a 3 % hydrogen peroxide solution in 70 % methanol for 20 min at room temperature. The sections were then washed three times in phosphate‐buffered saline (PBS) and subjected to heat‐induced antigen retrieval (HIAR) by incubation in a water bath with citrate buffer (pH 6.0) for 20 min after reaching 98 °C. To enhance antigen exposure, the buffer was supplemented with 2 mL of Triton X‐100 detergent (Sigma, Germany) per 200 mL, ensuring the symmetrical arrangement of slides within the cuvette [55].

Following three additional PBS washes, the slides were placed on a humidified plate and incubated with a 1 % normal goat serum solution in 1 % bovine serum albumin (BSA) for 20 min to block nonspecific binding. The primary antibodies used were as follows: Ki‐67 (rabbit polyclonal, 1:1000, Abcam, United Kingdom), CD34 (clone RM300, 1:400, Thermo Fisher Scientific, USA), and α‐SMA (clone 1A4, 1:800, Abcam, United Kingdom), CD3 (rabbit monoclonal (SP7), 1:150, Abcam, United Kingdom), CD45 (rabbit polyclonal, 1:2000, Abcam, United Kingdom), and CD68 (rabbit polyclonal, 1:1000, Abcam, United Kingdom).

Sections were incubated with primary antibodies overnight at 40 °C in a humid chamber. Immunodetection was performed using the Master Polymer Plus Detection System (Master Diagnostica, Spain), followed by visualization with a diaminobenzidine (DAB) chromogen reaction in the presence of hydrogen peroxide and horseradish peroxidase, producing a brown signal at antigen‐binding sites [55]. Counterstaining was performed with Gill's hematoxylin for 30 s. Finally, sections were dehydrated in graded alcohols, cleared in xylene, and mounted under coverslips using a permanent mounting medium.

### 2.17 Statistical Analysis

The mean and the standard error of the mean (SE) were calculated for each treatment group. For statistical comparison of wound diameter and area, ANOVA was followed by post‐hoc pairwise comparisons using the Tukey test. Data analysis was performed by Statistica (v14.1.0.8, TIBCO, Santa Clara, CA) software, p < 0.05 was considered statistically significant.

## 3 Results

### 3.1 SEM of the Nanofiber Wound Dressings

**FIGURE 1:**
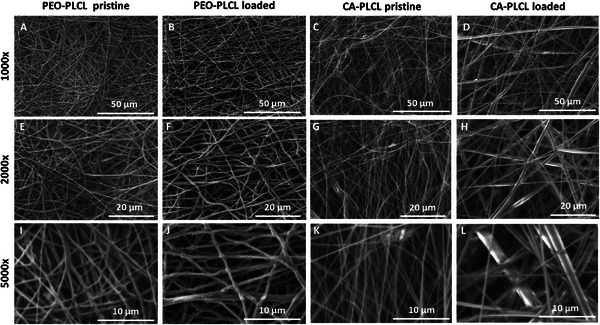
Scanning electron micrographs of pristine and drug‐loaded nanofibers at different magnifications (1000‐2000‐5000x).

**FIGURE 2:**
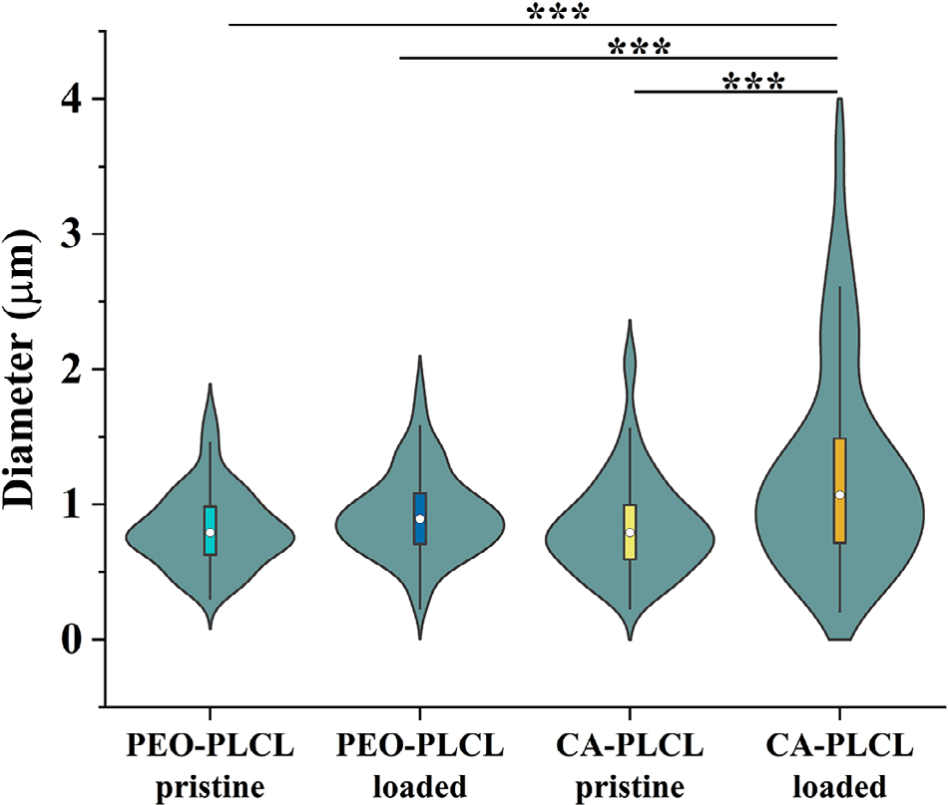
Violin plots show the distribution of nanofiber diameters. The inner box represents the interquartile range (25th to 75th percentile), with a white dot indicating the median.

**FIGURE 3:**
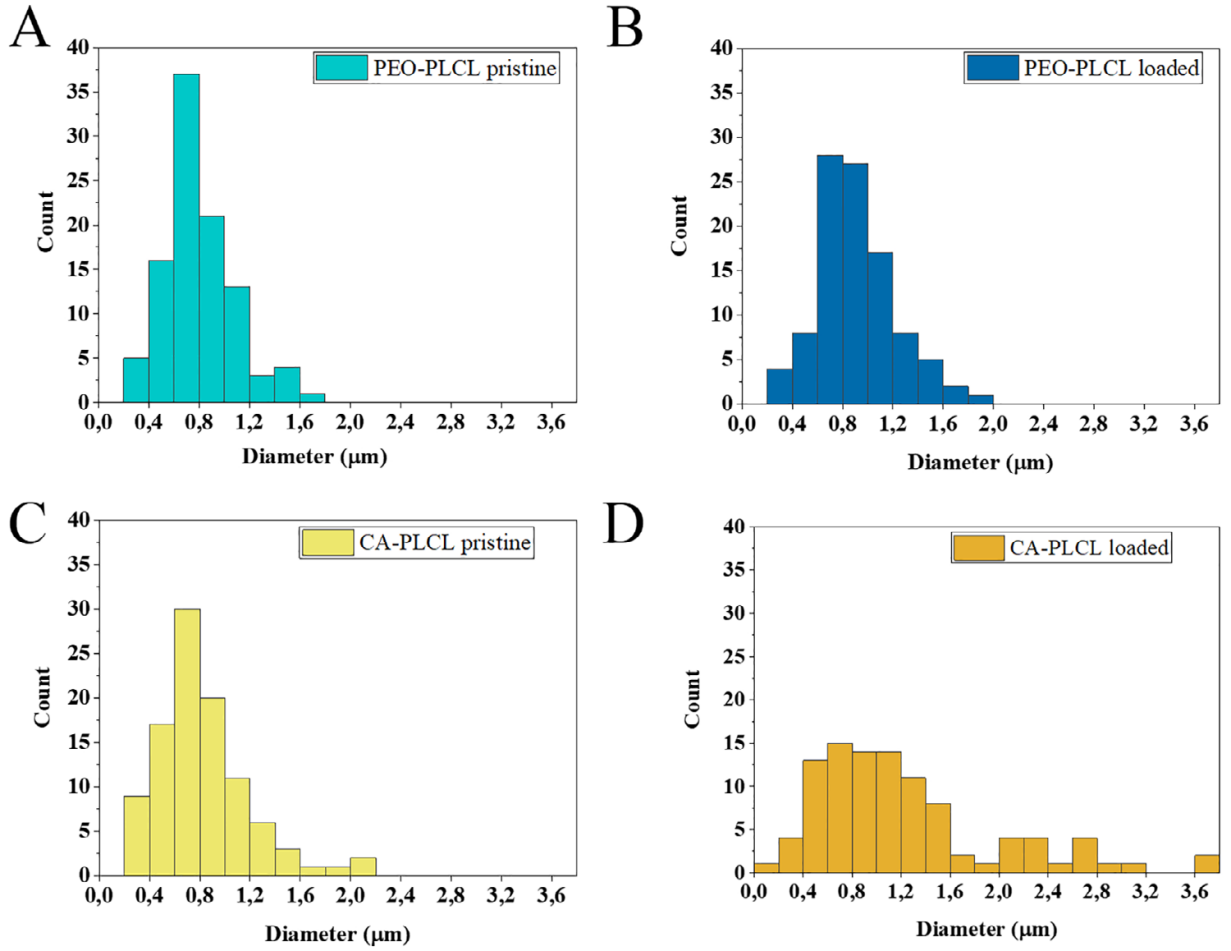
Histograms of the diameter distribution of the different nanofibers. (A) PEO‐PLCL pristine, (B) PEO‐PLCL drug loaded (dexamethasone, hyperbranched polymer, and ascorbic acid), (C) CA‐PLCL pristine, (D) CA‐PLCL drug loaded (dexamethasone, hyperbranched polymer, and ascorbic acid).

To characterize the size and variability in size of the pristine and drug loaded nanofibers, SEM and subsequent image analysis were performed (Figure 1). As seen in Figures 2 and 3, the diameter distribution peaked at 0.6–0.8 µm (36 and 30 %, respectively) in the pristine PEO‐PLCL and pristine CA‐PLCL nanofibers. After drug loading, the diameter distribution became more heterogeneous (around 15 %, from 0.4 to 1.6 µm), especially in CA‐PLCL‐loaded fibers.

The diameters of the pristine nanofibers were smaller (0.818 ± 0.028 and 0.845 ± 0.039 µm for the PEO‐PLCL and CA‐PLCL, respectively), whereas the drug‐loaded samples had larger diameters (1.075 ± 0.055 and 1.235 ± 0.075 µm for the PEO‐PLCL and CA‐PLCL nanofibers, respectively).

### 3.2 Water Uptake Capacity

The water swelling behavior of the matrices at 8 and 24 h is presented in Figure S4, and Table 2. Layer thickness (in mm) of pristine and drug loaded (dexamethasone and ascorbic acid) of hybrid electrospun wound dressings. shows the layer thickness (in mm) of pristine and drug loaded (dexamethasone and ascorbic acid) hybrid electrospun wound dressings. All samples absorbed substantial amounts of water; however, significant variations were observed between polymer types and formulations. The (PEO‐PLCL) pristine matrix exhibited a swelling degree of ∼399 % at 8 h, increasing to ∼663 % at 24 h, reflecting progressive hydration of the polymer network. The (PEO + PLCL based) loaded matrix absorbed ∼424 % at 8 h, but only reached ∼466 % after 24 h, suggesting that drug incorporation limited long‐term water penetration. The most pronounced swelling occurred in the (CA‐PLCL) pristine matrix, with uptake values exceeding 2800 % at 8 h and surpassing 3100 % at 24 h, indicative of a highly porous structure with rapid capillary‐driven water uptake. Drug incorporation substantially reduced swelling in the (CA‐PLCL) system, resulting in values around 1960 % at 8 h and 1940 % at 24 h, demonstrating that active components occupy or restrict internal pore volume. Overall, swelling plateaued between 8 and 24 h, indicating that most water absorption occurred within the first hydration phase (See Figure S4).

**TABLE 2:** Layer thickness (in mm) of pristine and drug‐loaded (dexamethasone and ascorbic acid) of hybrid electrospun wound dressings.

Nanofiber dressings	Layer thickness mean ± SD (mm)	n	Swelling (%)	
			8 h	24 h
PEO‐PLCL pristine	0.038 ± 0.002	3	399. 57	663. 09
PEO‐PLCL loaded	0.062 ± 0.015	3	424. 21	465. 87
CA‐PLCL pristine	0.048 ± 0.010	3	2806. 16	3118. 49
CA‐PLCL loaded	0.045 ± 0.002	3	1964. 62	1943. 87

These results demonstrate that all matrices exhibited substantial water absorption, consistent with the high surface area and porous architecture typical of electrospun scaffolds. However, marked differences emerged between (PEO‐PLCL) and (CA‐PLCL pristine) formulations. The (PEO‐PLCL) pristine matrix showed moderate but progressive swelling over 24 h, reflecting controlled water penetration into the polymer network. Drug loading increased the matrix thickness and slightly enhanced early swelling, but the overall 24 h uptake was lower compared to the pristine version. This reduction suggests that dexamethasone and ascorbic acid may partially occupy intermolecular spaces or influence polymer–water interactions, thereby limiting water access during the later stages of hydration.

Conversely, the (CA‐PLCL) pristine dressing exhibited remarkably high water uptake, exceeding 3000 % after 24 h. Such extreme swelling behavior is likely attributable not solely to the (CA‐PLCL) polymer's chemistry, but rather to the microstructural features produced during electrospinning—namely, high porosity, large void volume, and favorable capillary pathways that promote rapid fluid wicking. Interestingly, the incorporation of active compounds greatly attenuated this effect, reducing swelling by more than one‐third. Drug‐induced densification of the fibers or partial occlusion of inter‐fiber voids may explain this behavior, highlighting the sensitivity of (CA‐PLCL) nanofibers to compositional modifications. From a functional perspective, the high swelling capacity observed in both (PEO‐PLCL) and (CA‐PLCL) systems is advantageous for managing wound exudate. However, excessive swelling may compromise mechanical integrity or cause discomfort in a clinical setting. Drug‐induced modulation of swelling—especially in (CA‐PLCL) matrices—may therefore present a useful design strategy for tailoring dressings to specific wound environments, such as high‐exudate chronic wounds versus low‐exudate superficial injuries. The interplay between polymer (PEO‐PLCL), nanofiber architecture, and drug incorporation must be carefully optimized to achieve an ideal balance between absorbency, stability, and therapeutic performance.

### 3.3 Layer Thickness

The thickness of the electrospun matrices was evaluated to assess the structural consistency of the produced dressings. As shown in Table 3, the (PEO‐PLCL) pristine matrix exhibited an average thickness of 0.038 ± 0.002 mm, while drug loading increased this value to 0.062 ± 0.015 mm. This suggests that the incorporation of dexamethasone and ascorbic acid resulted in denser fiber deposition or an increase in average fiber diameter.

**TABLE 3:** TGA data of nanofibers highlight the main thermal events and total weight loss percentages.

Sample name	T_Onset_ (°C)	T_Endpoint_ (°C)	T_Inflection point_ (°C)	Weight loss (%)
CA‐PLCL pristine	333	399	371	85
CA‐PLCL loaded	330	406	369	86
PEO‐PLCL pristine	371	452	436	95
PEO‐PLCL loaded	386	456	442	93

In contrast, the (CA‐PLCL) matrices demonstrated comparable thickness values between pristine and loaded forms (0.048 ± 0.010 and 0.045 ± 0.002 mm, respectively), indicating that drug incorporation did not significantly alter their macroscopic structure.

Thickness measurements further support the above interpretations. (PEO‐PLCL) loaded matrices showed the greatest thickness, consistent with increased polymer mass and potential fiber diameter enlargement following drug incorporation. In (CA‐PLCL) matrices, however, loading had little influence on thickness, suggesting that the drug's impact manifests primarily in water‐fiber interactions rather than gross structural changes. These observations align with previous reports showing that additives can alter nanofiber morphology, crystallinity, and porosity, thereby affecting fluid absorption performance in wound dressings.

### 3.4 Tensile Strength

Mechanical testing revealed formulation dependent changes in stiffness and extensibility.

In hydrophilic PEO‐PLCL wound dressings, the applied drug loading increased Young's modulus (from 1.99 to 3.18 MPa) and reduced both elongation at maximum load and at rupture (1.68 to 0.79 mm and 5.22 to 1.67 mm, respectively), indicating a stiffer yet more brittle network.

In contrast, pristine CA‐PLCL samples exhibited the highest load bearing capacity and extensibility (F_max_ = 0.60 N, dL at F_max_ = 3.46 mm), whereas loading decreased F_max_ to 0.24 N and halved the elongation at maximum load without substantially altering modulus (∼1.15 MPa).

Together, these data show that drug incorporation predominantly compromises failure strain, especially in CA‐PLCL, while preserving or even increasing initial stiffness, which is critical for handling yet may limit conformability on moving tissue surfaces. The force (Fbreak) and length at break (dL) were unmeasurably low for CA‐PLCL loaded samples.

Across all pristine vs loaded comparisons, dL at break decreases markedly with loading (from 5.22 to 1.67 mm in PEO‐PLCL), supporting the conclusion that drug incorporation promotes earlier failure and reduced deformability in the case of the nanofiber‐based wound dressings of polymer compositions. For PEO‐PLCL, both pristine and loaded samples show relatively high extensibility but limited toughness: dL at break is high for pristine (5.22 mm) and still substantial for loaded (1.67 mm), confirming that they can be elongated visibly before failure. By CA‐PLCL samples, however, ductile separation could be observed; the separation resembled a sliding effect at slow external forces applied. Figure S3A,B shows typical force‐strain diagrams of the tested samples.

Mechanically, this matches a more ductile, fibrillating failure mode: under slow pulling, fibers and bundles reorient and slide, the mat necks and opens, and the structure gradually loses integrity rather than fracturing sharply. Drug loading reduces F_max_ (0.24 N) and dL at break (1.74 mm) without changing modulus substantially (∼1.14 MPa), within CA‐PLCL based dressings, the network architecture is weakened, and less force leads to a displacement.

### 3.5 Contact Angle, Zeta Potential, and Porosity

**FIGURE 4:**
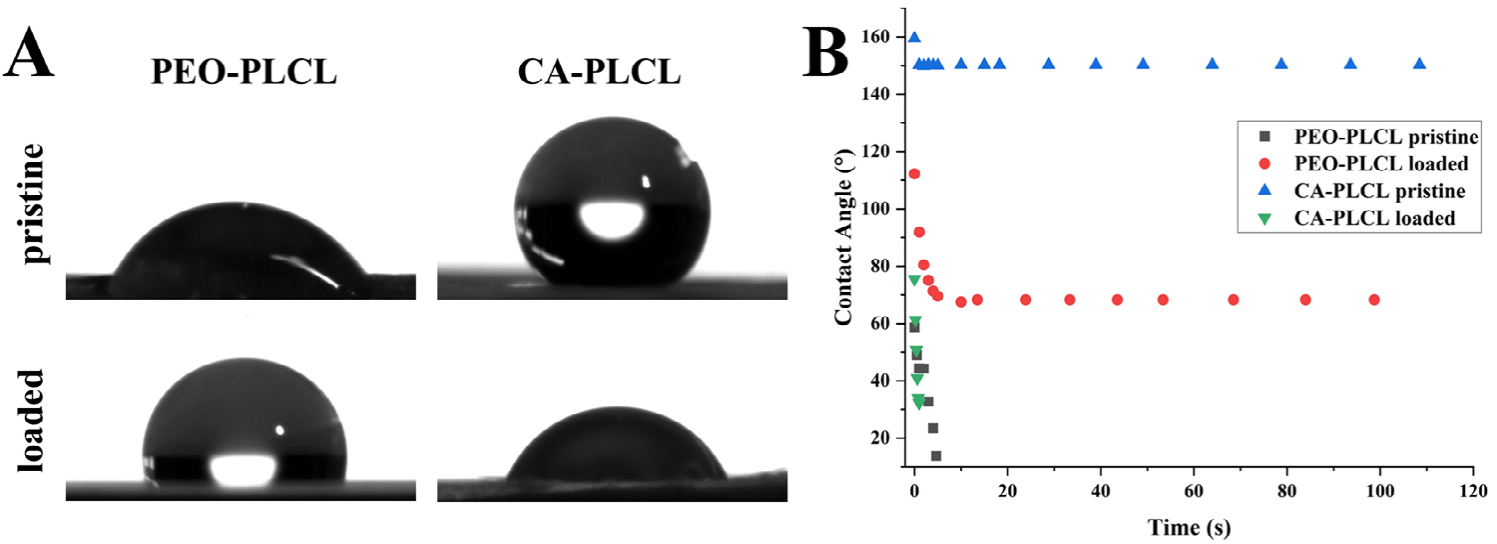
(A) Contact angle image of a water droplet on nanofiber patches at 0 s, (B) Change in contact angles of water droplets from 0 to 120 s.

An effective wound healing patch has certain wettability requirements that allow it to interact with the wound site. This interaction helps in maintaining the contact to allow bioactive molecules to function while preventing interaction with surrounding entities, which might cause harm or delay the wound healing. Furthermore, a good hydrophilic surface preserves hydration of the wound and prevents the buildup of exudates [56, 57]. Expectedly, the CA‐PLCL nanofibers were hydrophobic and showed the highest contact angle Figure 4. However, once these nanofibers were loaded with biomolecules, specifically dexamethasone, their contact angle decreased drastically. The contact angle or the wettability of nanofibrous non‐woven mats depends on multiple factors, including surface charge, materials, porosity, fiber diameter, and roughness, among others [58]. Due to TFA being used to prepare the solution for electrospinning, the low pH presumably protonated the dexamethasone and ascorbic acid, which resulted in a lowering of contact angles in CA‐PLCL loaded fibers. However, such an effect was not noticeable on the PEO‐PLCL fibers, where pristine fibers show a lower contact angle. While the loaded fibers show an increased contact angle initially, which eventually decreases. This might be caused by the presence of dexamethasone, which is generally a hydrophobic drug that was not protonated by formic acid [59], in contrast to the CA‐PLCL loaded solution. However, the overall wettability of nanofibers could still be considered within the range necessary for promoting a suitable wound healing environment [56, 58].

The zeta potential of dispersed nanofibers ranged from −8 mV for PEO‐PLCL pristine fibers to 24 mV for PEO‐PLCL loaded fibers (Figure S2). Drug loading led to an increase in zeta potential for these fibers, as well as for the CA‐PLCL fibers, albeit to a lesser extent. Here, an increase in zeta potential by drug loading from 2 to 15 mV can be observed. For both kinds of fibers, the incorporation of positive charges is indicated. Zeta potentials ranging from 50 to −30 mV were shown to enhance cell proliferation without cytotoxic effects [60], with higher zeta potentials being reported to support cell and protein interactions [61].

The porosity of the fibers was measured using the liquid displacement by a pycnometer, showing a standard behavior for the 2D nanofiber membranes [48, 62]. It was observed that the wetting behavior of the nanofibers in ethanol was different owing to their composition. However, with the application of a vacuum, it was possible to ensure the optimal swelling and uptake of the ethanol. PEO/PLCL pristine fibers show a measured porosity of 78.5 % as compared to 86.6 % after drug loading. While CA‐PLCL showed a porosity of 72.7 % as compared to 82.7 % for loaded fibers. Presumably, an increase in the size of the fibers led to lower packing densities during electrospinning. This can lead to bigger pores and hence an increase in the porosity.

### 3.6 Thermogravimetric Analysis (TGA)

**FIGURE 5:**
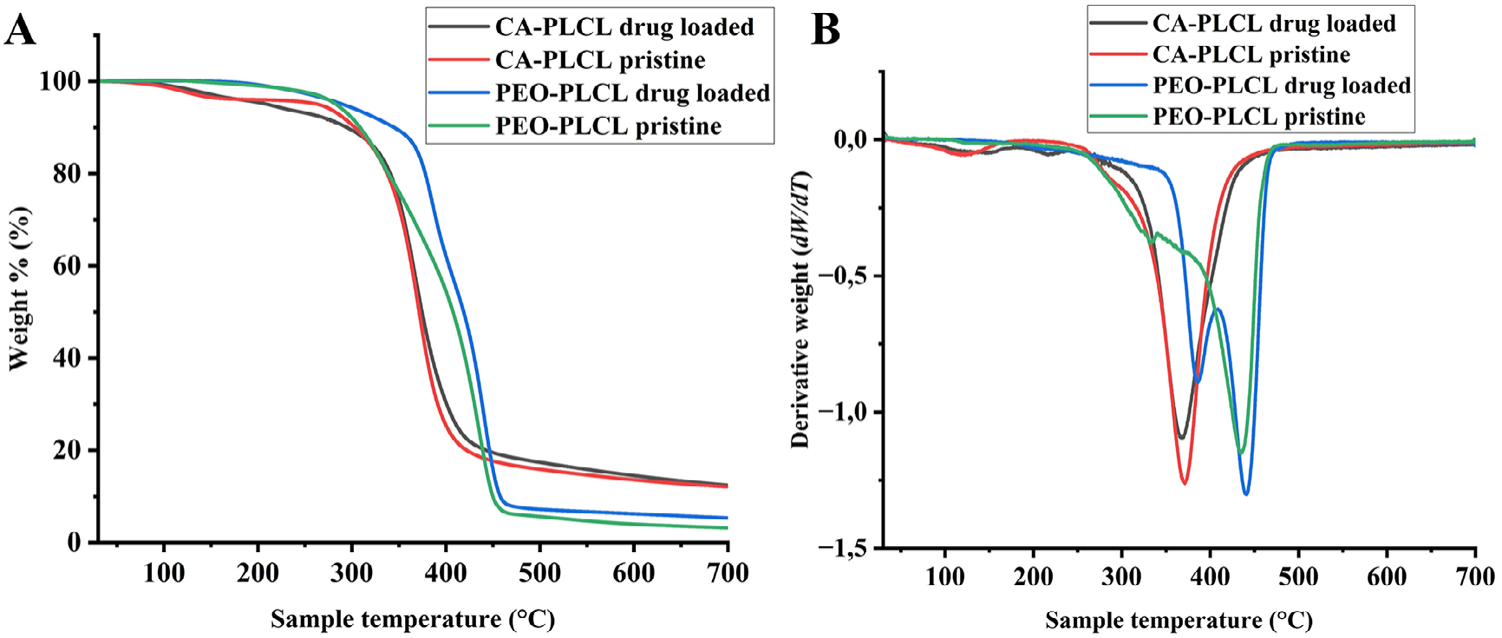
TGA (A) and derivative thermogravimetry (DTG) (B) data from nanofibers show the weight loss behavior of the nanofibers after subjecting them to a temperature program from 30 °C to 700 °C at 10 °C min^−1^.

Thermal analysis of nanofibers using TGA showed that both types of nanofibers, including the pristine fibers, showed a multistep degradation behavior. As seen in Figure 5, CA‐PLCL pristine and loaded fibers showed a gradual loss of weight starting around 100 °C up to 160 °C, which corresponds to the degradation of hydrogen bonds among CA and PLCL polymers. The major weight loss for these samples related to the decomposition of polymers occurred at 369 °C for drug loaded CA‐PLCL nanofibers and 371 °C for pristine nanofibers, as compared in Table 3 and Figure 5B, which was used to calculate the inflection point of nanofibers [63]. Interestingly, the weight loss events recorded for PEO‐PLCL nanofibers were distanced due to the wide variance in the thermal properties of the polymers blended (PEO / PLCL) for the generation of nanofibers. A weight loss event related to the decomposition of PEO can be noticed at 284 °C and 294 °C for pristine fibers and drug loaded nanofibers, respectively [34]. The onset temperature was recorded at 371 °C and 386 °C for pristine and loaded nanofibers, respectively. The inflection points for PEO‐PLCL nanofibers showed that the composition of PEO and PLCL, along with other biomolecules, decomposed fastest from 436 °C to 442 °C. The end of degradation temperature for CA‐PLCL nanofibers was measured at 399 °C and 406 °C for pristine and loaded nanofibers, respectively. They showed a weight loss of 85 %–86 %. Whereas the end of degradation temperature for the PEO‐PLCL nanofibers was recorded between 452 °C and 456 °C. These fibers were degraded almost completely with a measured weight loss of 93 %–95 %. The loading of nanofibers did not have a significant impact on their overall weight loss behavior, and polymers, being the main components, had a greater influence.

### 3.7 Attenuated Total Reflection‐ Fourier‐Transform Infrared Spectroscopy (ATR‐FTIR)

**FIGURE 6:**
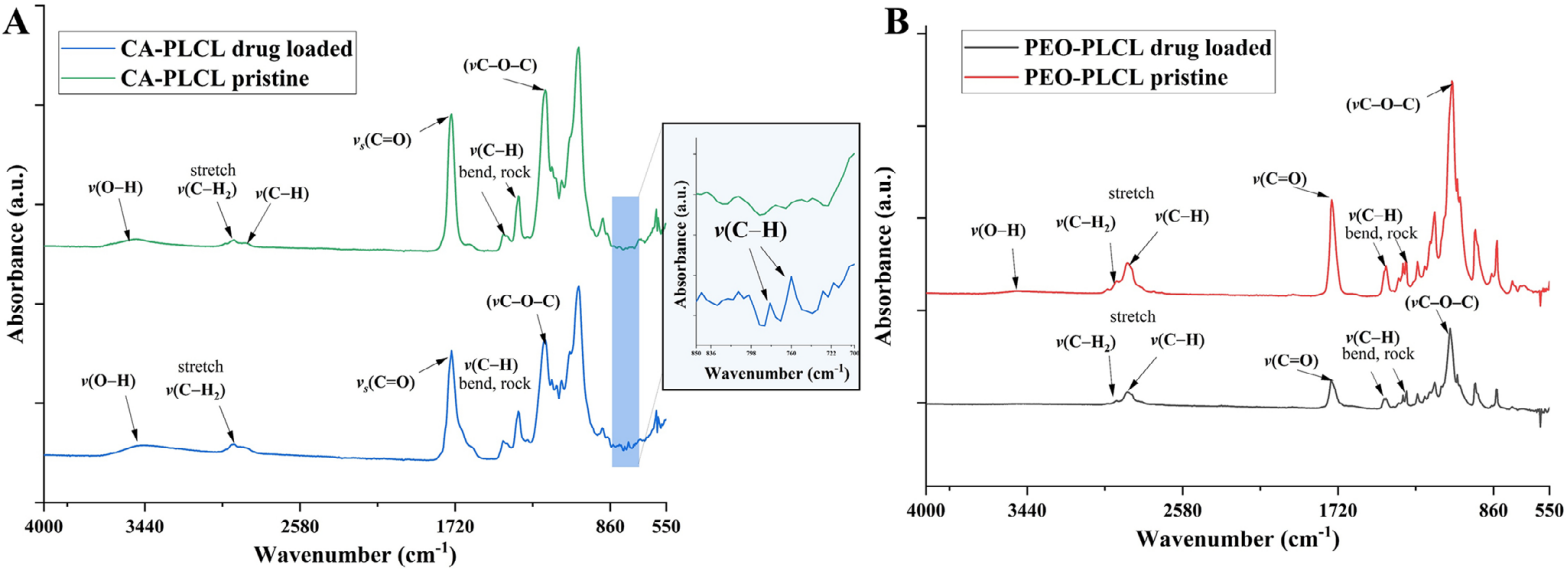
Characteristic ATR‐FTIR absorbance peaks were observed from (A) CA‐PLCL nanofibers and (B) PEO‐PLCL nanofibers.

The interaction of various biomolecules and polymers used for the fabrication of nanofibers was analyzed using ATR‐FTIR. As seen in Figure 6, the various absorbance peaks related to the vibration frequency of prominent bonds have been recorded from 4000 to 550 cm^−1^. CA‐PLCL nanofibers show an absorbance peak related to the vibration of νO─H around 3500 cm^−1^. The samples indicate absorption bands associated with methylene (∼2960 cm^−1^) and methyl (∼2850 cm^−1^) groups. Both pristine and loaded nanofibers demonstrated a strong single νC═O vibration absorption peak at 1720 cm^−1^. Further, absorbance peaks associated with bending and rocking vibrations of νC─H at 1460 and 1370 cm^−1^, respectively, are observed. There were νC─H stretching absorbance peaks, assigned to dexamethasone, present in the spectra of loaded nanofibers [64, 65]. The absorbance spectra of PEO‐PLCL pristine nanofibers and loaded nanofibers are almost identical aside from insignificant differences in the absorbance intensity and the presence of the weak νO─H absorbance band around 3500 cm^−1^ for the pristine sample. Characteristic methylene (∼2960 cm^−1^) and methyl (∼2850 cm^−1^) groups, a single νC═O vibration absorption peak at 1720 cm^−1^, and bending and rocking vibrations of νC─H at 1460 and 1370 cm^−1^ were recorded. Additionally, the νC─O─C stretch typical of carboxylic acids and esters was observed in both samples.

### 3.8 In Vitro Cytotoxicity (MTT Assay)

It was not possible to investigate the cytotoxicity of the nanofibers in direct contact with the cells because the electrospun wound dressings were floating on the surface of the culture medium. Therefore, they had to be frozen, ground, and dissolved as described in the methodological part. Based on preliminary results, the higher concentrations (>1 mg mL^−1^) of the nanofibers (both CA‐PLCL and PEO‐PLCL) reduced the viability of the cells, while the lower concentrations (1, 0.5, 0.25 mg mL^−1^) were not cytotoxic to the cells (neither on fibroblasts nor on the keratinocytes) (Figure S5). Additionally, the active components embedded in the nanofibers, dexamethasone and ascorbic acid, were independently tested (Figure S6). Dexamethasone and ascorbic acid were not toxic to the cells at the concentration used to functionalize nanofibers (0.01–5.0 µm). Dexamethasone had a generally positive effect on metabolic rate, and ascorbic acid was only toxic under higher concentrations (5 m). These results suggest that the observed cytotoxicity at higher nanofiber concentrations could be due to the presence of a high concentration of bioactive compound (ascorbic acid) and the combined formulation with nanofibers.

### 3.9 Ultrasound and Optical Imaging of the in Vivo Wound Healing Process

**FIGURE 7:**
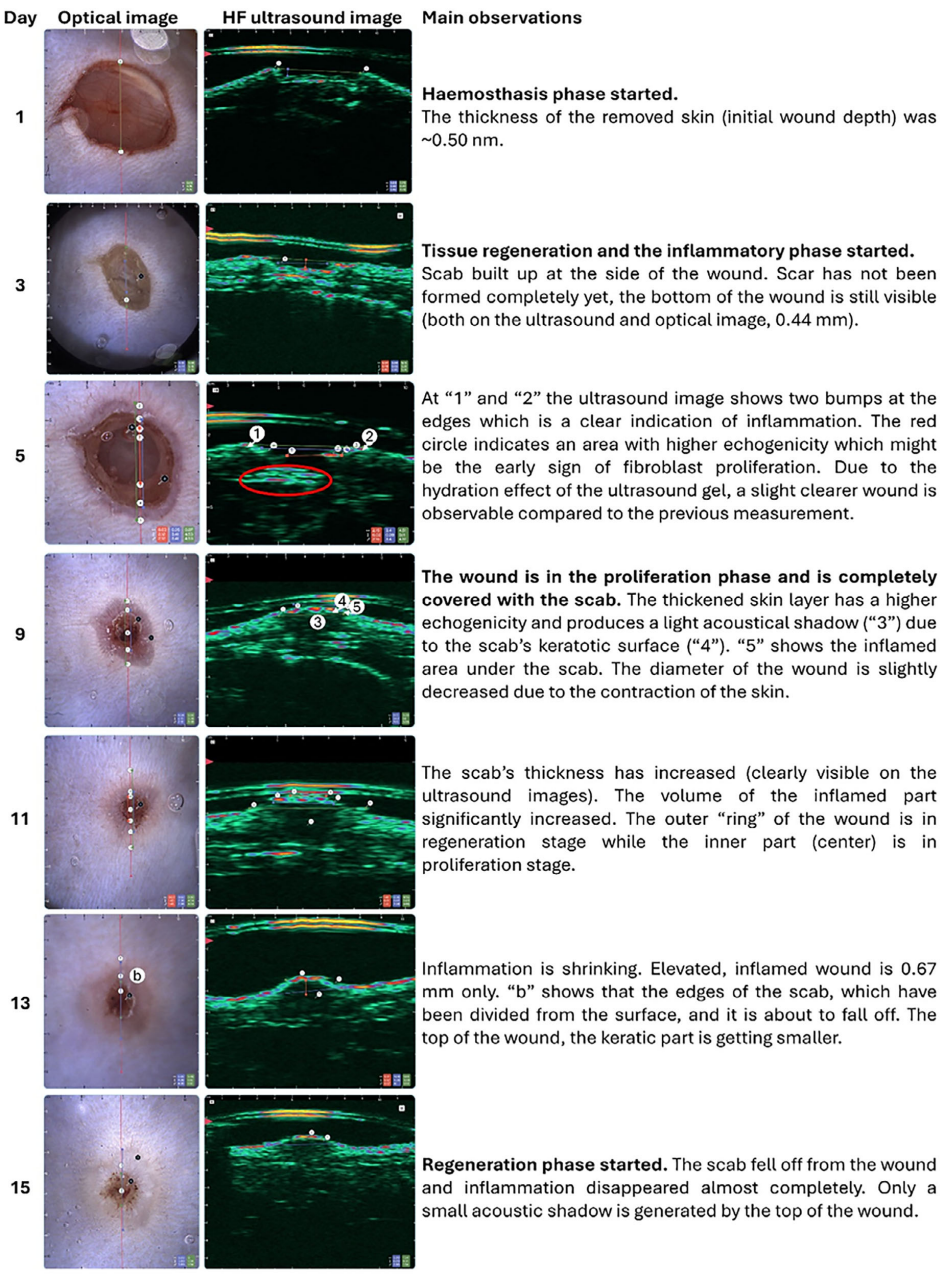
Process of wound healing in the dorsal skin surface of an SKH1 control (untreated) mouse monitored by optical (left) and high‐frequency ultrasound (right) imaging.

**FIGURE 8:**
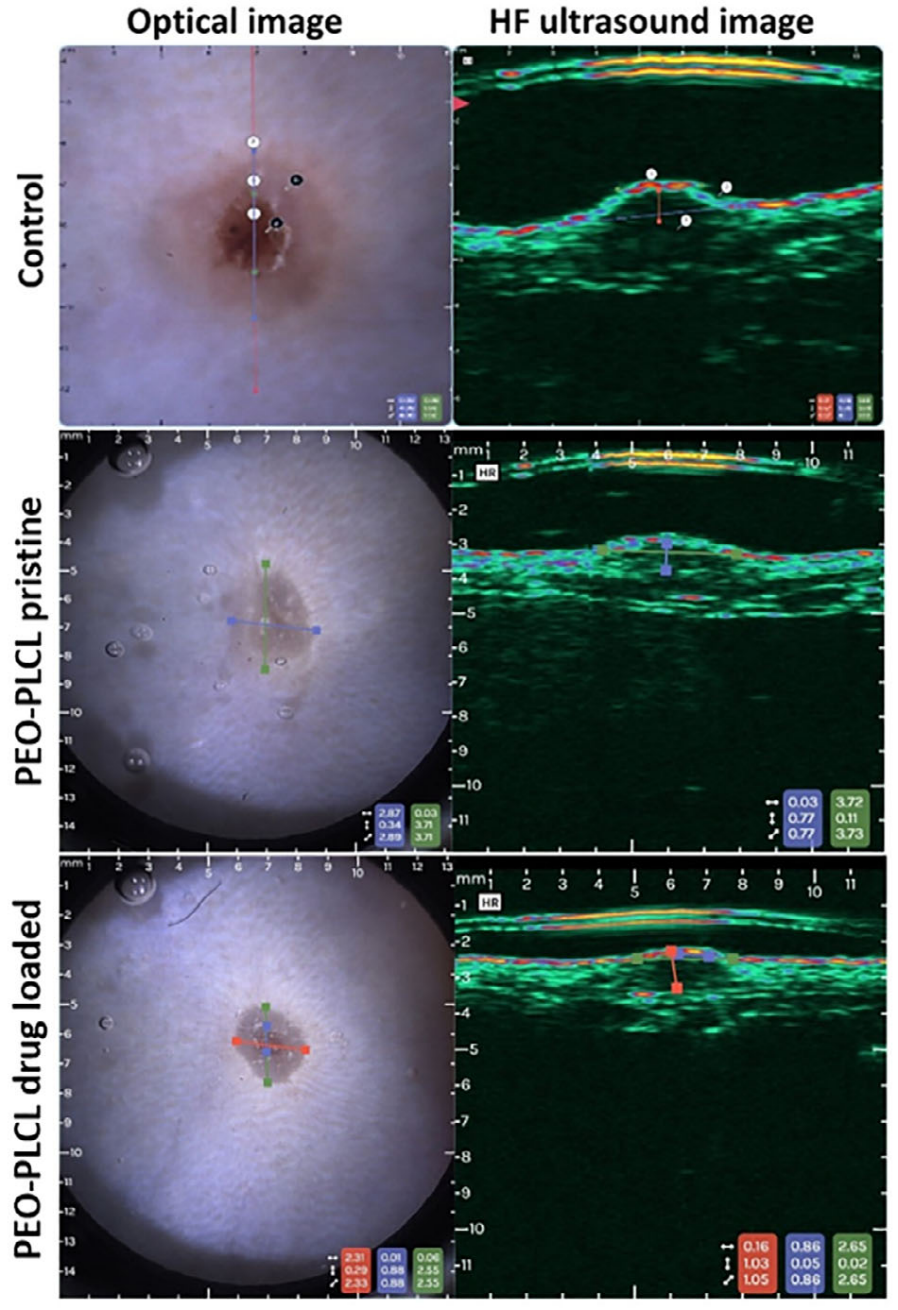
Status of wound closure on day 13 after wounding in SKH1 mice (control, treated with PEO‐PLCL pristine and PEO‐PLCL drug‐loaded wound dressings) by optical (left) and high frequency ultrasound (right) imaging.

Optically assisted high‐frequency ultrasound images were taken from one control animal at different time points. The size of the wound was measured on the digital images (Table S2). Figure 7 demonstrates the physiological and pathological processes during natural wound healing in the control mouse from 1 to 15 days. While Figure 8 compares the status of the wound in one representative animal from the control, PEO‐PLCL pristine, and PEO‐PLCL drug‐loaded groups on day 13, when the remodeling and shrinking processes are dominant. However, no significant difference can be detected between the drug‐loaded and pristine nanofibers. Both are effective in the acceleration of the healing process. Similarly, to the image analysis of camera pictures (Figure S7), both the unloaded and the drug‐containing nanofibers were equally beneficial, probably due to the antimicrobial and adhesive effects published earlier by other authors of the pristine nanofibers themselves [66, 67]. These findings suggest that some modifications of concentration and optimization of the drug release kinetics could be necessary in the dressings to further enhance wound healing outcomes.

Integrating dermoscopy‐assisted high‐frequency ultrasound (derma‐HFUS) with optical imaging in our study represents a state‐of‐the‐art approach for non‐invasive wound monitoring. Unlike traditional methods relying solely on surface photography or histology, this dual‐modality system provides real‐time insights into superficial and subdermal tissue changes. Recent literature demonstrates that high‐frequency ultrasound can sensitively detect early alterations in tissue structure, such as scar formation and granulation tissue dynamics (e.g., Benavides et al. [50]; Losi et al. [51]), while optical imaging delivers high‐resolution surface details [50]. Our findings, which capture wound contraction and tissue remodeling over the 15‐day period, are in line with these advancements and suggest that the combined imaging strategy can enhance the accuracy of wound assessment and potentially facilitate the translation of such techniques into clinical practice.

### 3.10 Morphological Changes During the Wound Healing Process

**FIGURE 9:**
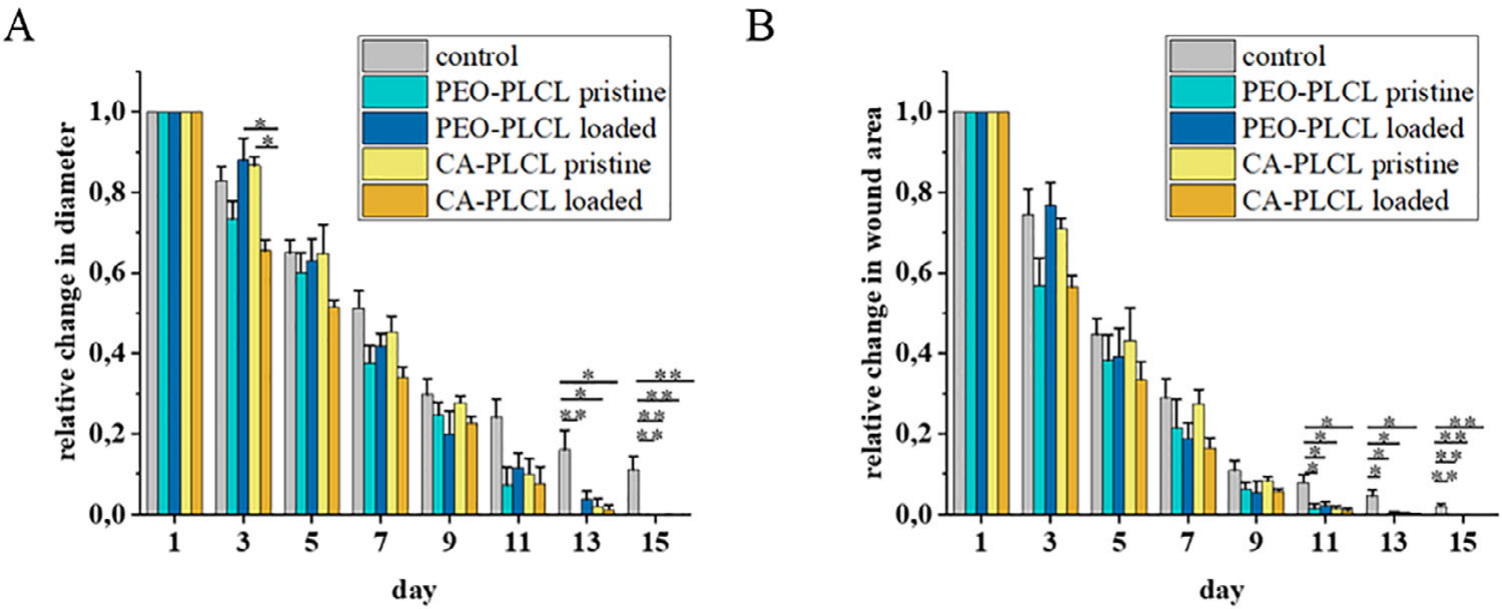
Changes in wound diameters (A) and areas (B) during the wound healing process in SKH1 mice (controls, treated with CA‐PLCL pristine, CA‐PLCL drug‐loaded, PEO‐PLCL pristine, and PEO‐PLCL drug‐loaded nanofiber‐based wound dressings). *n* = 5/group, ^*^: *p* < 0.05, ^**^: *p* < 0.01, ^***^: *p* < 0.001.

The morphological changes and the shrinkage of the wounds were also monitored by camera imaging, followed by image analysis. The relative diameters (Figure 9A) and wounded areas (Figure 9B) were calculated.

Remarkable changes can be seen in the wound size in the second phase of the healing process. During the early hemostasis and inflammation, the control and wound dressing treated groups were similar, while in the phase of cell proliferation, granulation, and remodeling, all nanofiber‐covered groups showed accelerated wound closure. However, there was no statistically significant difference between the drug‐containing and pristine nanofibers. Based on these results, some further modifications could be needed in the composition of our bandages. e.g., the concentrations of the drugs loaded are not sufficient, or probably due to too strong binding and interaction between the drugs and the surface of the nanofibers, the release is not appropriate. These factors should be studied in detail and optimized to achieve more effective dressings.

The quantitative analysis of wound morphology using digital image processing tools, such as ImageJ, complements the advanced imaging modalities used in our work and is comparable to current state‐of‐the‐art methods. Modern wound assessment studies increasingly employ automated segmentation and morphometric analyses to reduce subjectivity and improve reproducibility (see, for example, Losi et al. [51]; Benavides et al. [50]). While some recent approaches incorporate machine learning for even finer resolution in wound edge detection, our methodology offers a robust yet accessible means to monitor wound closure. This balanced approach not only supports the utility of our imaging data but also highlights the potential for combining traditional image analysis with advanced imaging modalities for comprehensive wound evaluation.

### 3.11 Histology and Regeneration Biomarkers of Wound Tissues at 15 Days After Wounding

**FIGURE 10:**
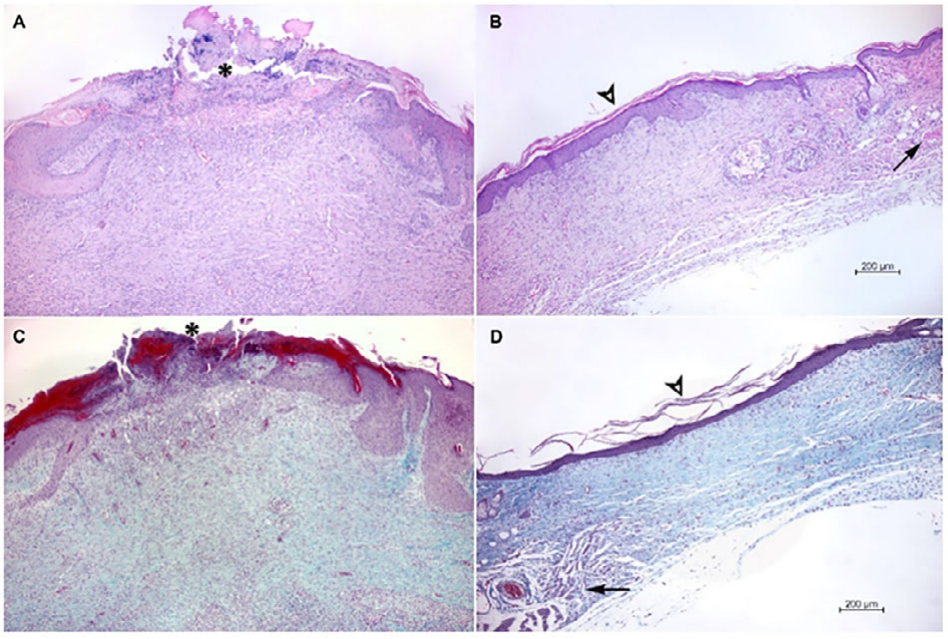
Histological view of the lesions after 15 days. Representative images. Control: (A) and (C) (left); nanofiber‐treated wounds (on right): PEO‐PLCL loaded (B) and CA‐PLCL pristine (D). Both images of the control group on the left show an incompletely epithelialized wound covered with fibrinous masses (asterisk). On the right, the wounds treated with the wound dressings are completely epithelialized (arrowhead). There is a disruption of the *panniculus carnosus*  (arrow). Low magnification (×50), hematoxylin‐eosin (A,B), and Goldner's trichrome (C,D) staining.

Following a 15‐day treatment period, significant histological variations were detected between the control group and the wounds treated with wound dressings. In the control samples (Figure 10A,C), the wounds demonstrated incomplete epithelialization and were characterized by the presence of fibrinous masses. The wound bed demonstrated marked inflammation, substantial granulation tissue, and underlying fibrosis. The presence of epidermal hyperplasia at the wound margins is indicative of ongoing reparative processes.

Conversely, all wounds treated with wound dressings – irrespective of the type – demonstrated complete epithelialization (Figure 10B,D). The newly formed scar tissue exhibited a reduced thickness and a diminished blood vessel density in comparison to the control group. The *panniculus carnosus* exhibited an abrupt interruption at the wound site across all treated groups, transitioning into scar tissue. While no skin appendages were preserved amid the scar, some residual appendageal units, incidentally with cystic changes, were observed on the periphery of the scar in proximity to the disruptions of the *panniculus carnosus*. Samples treated with both variants of CA‐PLCL nanofiber dressings (pristine and drug loaded) exhibited minimal neutrophilic infiltration around the scar tissue. In comparison, both PEO‐PLCL‐treated samples exhibited significant neutrophilic infiltration around the remnants of pilosebaceous units in the vicinity of the scar defect. Furthermore, samples that had been treated with drug loaded PEO‐PLCL dressings exhibited the presence of minor residual areas of granulation tissue.

**FIGURE 11:**
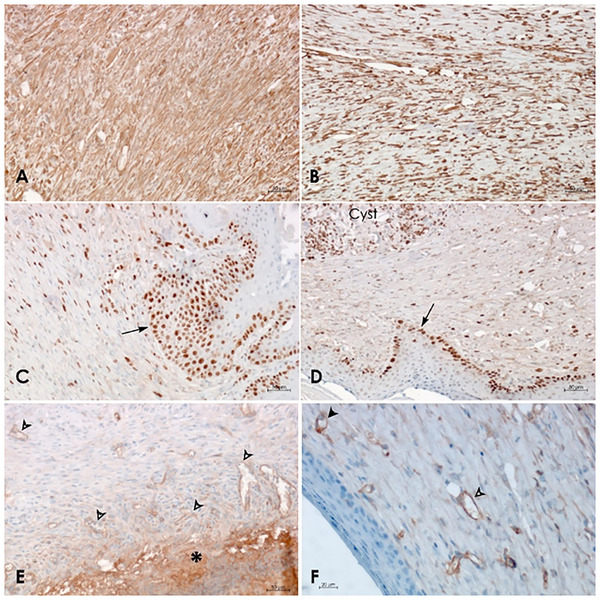
Representative pictures of expression of selected markers after 15 days in control samples (on left: A,C,E) and in wounds treated with the wound dressings (on right: B,D,F). The number of myofibroblasts in the groups with all types of wound dressings was significantly lower (B, CA‐PLCL loaded group) than in the control group (A), as evidenced by the presence of α‐SMA expression. The conspicuous proliferation of basal epidermal cells at the wound edge of the control group (arrow, C) in comparison with the group of drug loaded PEO‐PLCL wound dressing (arrow, D) was confirmed by the nuclear Ki‐67 expression. Note the proliferating myofibroblasts in the scar and nested leukocytes (macrophages) in the cyst, which probably originated from the remains of skin appendages. CD34 staining: a significant number of the capillary‐like vessels (arrowheads) in granulation tissue under the fibrinous scab (asterisk) of the wound in the control sample (E) contrasts with the number of vessels in the CA‐PLCL loaded group (F).

Immunohistochemical analysis further highlighted differences in the expression of selected markers between the groups. The expression of α‐SMA revealed a higher number of myofibroblasts in the control group (Figure 11A) in comparison to the groups treated with the investigated wound dressings (Figure 11B). Ki‐67 staining revealed a marked increase in basal epidermal cell proliferation at the wound edge in the control group (Figure 11C), while the groups treated with experimental wound dressings (Figure 11D) exhibited a decrease in proliferative activity. CD34 staining revealed a substantial presence of capillary‐like vessels in the granulation tissue beneath the fibrinous scab in the control wounds (Figure 11E). This finding is in contrast to the reduced vascular density observed in all experimental groups (Figure 11F).

**FIGURE 12:**
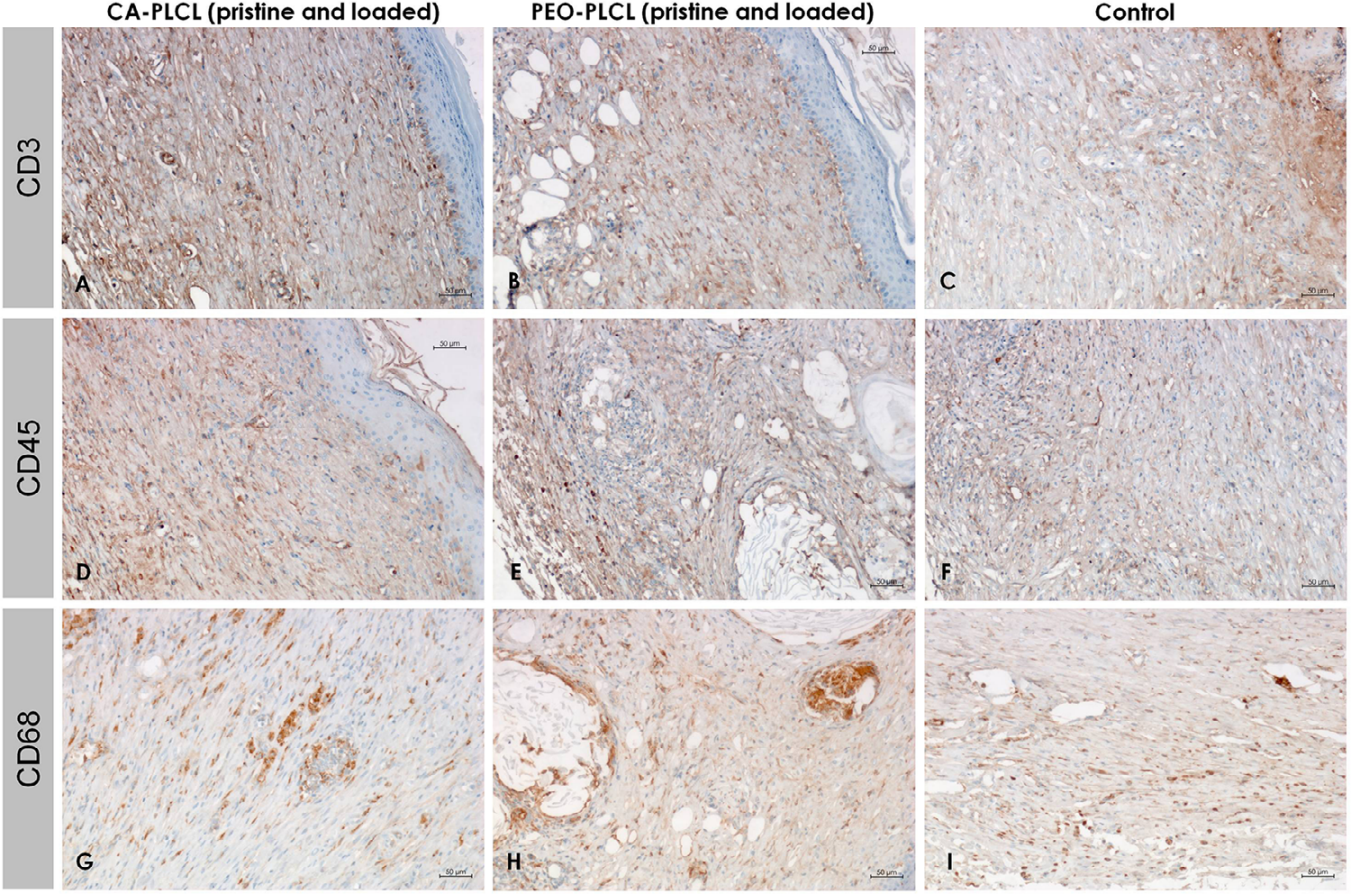
Representative images of CD3, CD45, and CD68‐positive cells distribution in wounds treated with the CA‐PLCL pristine (A) and loaded (D,G) dressings samples (on left: A,D,G), in wounds treated with the PEO‐PLCL pristine (E,H) and loaded (B) dressings (in the middle: B,E,H), and in control samples (on right: C,F,I). The inflammatory level of CD3, CD45, and CD68 in the wound scar of the groups with all types of dressings was sufficiently lower (A,B,D,E,G,H) than in the control group (C,F,I). In addition, no statistically significant differences were observed between the experimental groups. Immunoperoxidase method, x200.

To assess the inflammation rates and compare the investigated groups with the control, the quantity of CD3, CD45, and CD68‐positive cells was evaluated by immunohistochemical analysis. CD3 staining revealed a low level of T‐cell infiltration in the dermis of the groups treated with the wound dressings PEO‐PLCL (pristine and loaded), alongside CA‐PLCL (pristine and loaded), without any statistical difference. According to the semiquantitative evaluation, CD3‐positive cells distribution throughout the dermal layers indicated a mild score (1–5 cells per high‐powered FOV). Overall, the occurrence of CD3‐positive cells was significantly lower in all experimental samples (Figure 12A,B) compared to the control group (Figure 12C). Similarly, the staining for CD45, a pan‐leukocyte marker, exhibited lower cellular infiltration in the scar tissue of both the PEO‐PLCL and CA‐PLCL groups in comparison with the control group (Figure 12D,F). However, dense CD45‐positive neutrophilic infiltration was observed around the remnants of pilosebaceous units in PEO‐PLCL‐treated samples, as was mentioned above (Figure 12E). In contrast, the experimental dressings showed only scattered CD45‐positive cells, confirming attenuated leukocyte infiltration. The evaluation of CD68, a protein associated with macrophages, revealed the presence of CD68‐positive cells within various regions of the dermis in all experimental groups. The cells were found to be diffusely distributed but present in low numbers. The number of CD68‐positive macrophages was found to be significantly lower in all of the groups that had been treated with experimental dressings (PEO‐PLCL and CA‐PLCL, both pristine and loaded) in comparison to the control wounds (Figure 12I). In the control samples, located beneath the unhealed wound surface, a large number of inflammatory cells (macrophages, neutrophils, and lymphocytes) were detected, reflecting an ongoing inflammatory phase (Figure 12C,F,I). It is noteworthy that no statistically significant differences were observed between the PEO‐PLCL and CA‐PLCL groups, as well as between the pristine and drug loaded modifications, indicating that all tested types of dressings effectively suppressed inflammatory cell infiltration to a similar extent.

## 4 Discussion

Based on the results of the in vivo wound healing promoted by nanofiber dressings and the morphological and histological analysis of the scar tissues, furthermore considering our unpublished preliminary drug‐release data on the electrospun dressings, it can be concluded that the nanofibers (both PEO‐PLCL and CA‐PLCL), regardless of whether they contained dexamethasone and ascorbic acid, were effective in the late stages of the healing process. This observation is consistent with prior findings where electrospun nanofibers alone were sufficient to promote wound closure due to their structural mimicry of the extracellular matrix and high surface area, promoting cell adhesion and proliferation [2, 3, 14].

Although both pristine and drug‐loaded electrospun nanofibers accelerated wound healing, several limitations should be noted. First, no significant differences were observed between drug‐loaded and pristine fibers, suggesting that the concentrations of dexamethasone and ascorbic acid used may have been suboptimal, or that their release kinetics were not adequately tuned. The hydrophobicity and binding affinity of the incorporated drugs may also have limited their bioavailability at the wound site. Additionally, cytotoxicity testing was restricted to indirect assays, as the nanofibers could not be assessed in direct cell–material contact, which may not fully reflect in vivo interactions. The in vivo evaluation was performed in a murine model with acute wounds, which does not fully mimic the complexity of chronic or infected wounds in humans. Furthermore, the study duration (15 days) was relatively short and did not capture long‐term scar remodeling or functional recovery of skin appendages. Summarily, several factors could be behind the lack of difference between pristine and drug‐loaded nanofibers: (1) The concentration of the applied active components (dexamethasone and/or ascorbic acid) may not have reached pharmacologically relevant levels to exert a measurable effect [7, 64]. (2) The poor water solubility of dexamethasone may have hindered its release and absorption at the wound site, as observed in other polymer‐drug systems [59, 65]. (3) The binding affinity of the active ingredients to the nanofiber surface may have impeded their dissociation within the wound microenvironment, limiting bioavailability — an issue also seen in other electrospun systems incorporating hydrophobic drugs [64].

This study demonstrates that electrospun nanofiber matrices composed of (PEO‐PLCL) or (CA‐PLCL) polymers exhibit distinct swelling behaviors and structural characteristics that are strongly influenced by drug incorporation. PEO‐PLCL dressings showed moderate and gradual swelling, while CA‐PLCL pristine matrices absorbed exceptionally high volumes of water due to their porous architecture. Drug loading reduced swelling in both types of matrices, with a particularly strong effect in CA‐PLCL formulations, suggesting that active compounds alter internal fiber morphology or pore structure. These findings emphasize the importance of carefully selecting polymer composition and drug incorporation strategies when designing advanced wound dressings. Modulating water uptake through formulation design enables tailoring of dressings for specific wound types, improving exudate and bleeding control, comfort, and therapeutic performance. The results provide a foundation for further optimization of electrospun, drug‐loaded hybrid wound dressings aimed at enhancing wound healing outcomes.

Considering all of these factors, further optimization is needed. In line with current research trends, our next steps include the evaluation of steroidal and non‐steroidal anti‐inflammatory agents with higher aqueous solubility, and the use of solubility enhancers such as β‐cyclodextrins, which have shown promise in improving drug dispersion and bioavailability in nanofiber matrices [12, 40]. Moreover, core‐shell nanofiber architectures are widely reported as effective in achieving controlled, stage‐specific release of therapeutic agents and should be investigated as an advanced design alternative [12, 30]. Additionally, the incorporation of hemostatic components like gelatin, collagen, or chitosan, and growth factors such as vascular endothelial growth factor (VEGF), fibroblast growth factor (FGF), or platelet‐derived growth factor (PDGF), could enhance angiogenesis and granulation tissue formation during the early healing stages, as shown in other studies [6, 10, 20, 30]. For example, Wang et al. demonstrated that mussel‐inspired core‐shell nanofibers with embedded growth factors exhibited superior wound healing efficacy by enabling sustained release and maintaining bioactivity [30]. Furthermore, a detailed physicochemical analysis, such as differential scanning calorimetry (DSC), X‐ray diffraction (XRD), or Raman spectroscopy, could be performed to investigate the crystallinity and dispersion state of the loaded active factors known to influence release kinetics and therapeutic activity [12, 63]. Self‐healing hydrogels in combination with biodegradable polymers and nanoparticles have been used to promote wound healing in diabetic wounds [68]. However, the biodegradability of the nanoparticle and their resorbability have not been fully understood. Similarly, multifunctional PVA nanofibers functionalized using polyhexamethylene biguanidine and silver nanoparticles as antibacterial agents proposed for treating burn wounds have shown promising results [69]. However, the use of silver nanoparticles can have an adverse effect on the overall cytotoxicity of the material itself. Currently, the wound healing approach involves multifunctional nanofibers based on bioactive polymers and functionalized agents such as gelatin, lumbrokinase, and plant source polyphenols such as quercetin, tannic acid, curcumin, and resveratrol [70, 71, 72, 73, 74, 75]. Furthermore, developing nanofibers with 3D architecture is another strategy to overcome the sustained issue of cell proliferation, bioresorbability, toxicity, and biocompatibility due to the use of synthetic drugs while promoting a holistic wound healing approach [76]. Overall, our findings reinforce the utility of electrospun nanofibers as an advanced wound dressing platform and highlight the necessity of fine‐tuning composition and drug‐release parameters to fully harness their therapeutic potential.

## 5 Conclusion

This study demonstrated the successful fabrication and in vivo evaluation of multifunctional electrospun nanofiber wound dressings composed of PEO‐PLCL and CA‐PLCL polymer matrices, enhanced with dexamethasone, ascorbic acid, and hyperbranched polymers. The nanofibers exhibited suitable morphological characteristics, controlled wettability, and thermal stability, confirming their structural and physicochemical integrity. Preliminary in vitro studies showed a low toxicity and good biocompatibility of these nanofibers. In vivo wound healing experiments in nude mice indicated that both pristine and drug loaded nanofibers significantly accelerated wound closure and promoted better epithelialization and scar formation compared to untreated controls. Histological and immunohistochemical analyses further validated the reduction in inflammation, enhancement of tissue remodeling, and decrease in neovascularization in nanofiber‐treated wounds. By integrating responsive nanofiber scaffolds with multimodal imaging (derma‐HFUS and digital morphometric analysis), this work contributes to the evolving field of bioactive wound dressings and establishes a robust platform for non‐invasive, longitudinal assessment of healing. These findings align with the growing emphasis on precision biomaterials and real‐time monitoring technologies in regenerative medicine and offer a translational pathway toward clinically relevant, next‐generation wound care solutions.

Despite the promising therapeutic outcomes, no significant differences were observed between drug‐loaded and pristine fibers in terms of wound healing efficacy. This suggests that while the base polymer matrices themselves exhibit beneficial antimicrobial and adhesive properties, the release kinetics or bioavailability of the incorporated bioactives may be suboptimal. Future work should therefore focus on optimizing drug loading strategies and release kinetics, potentially by employing core–shell electrospinning, responsive polymer systems, or solubility enhancers such as cyclodextrins. Incorporating bioactive molecules such as growth factors, collagen, or chitosan could further enhance angiogenesis and tissue regeneration. Long‐term studies in clinically relevant chronic and infected wound models will be necessary to confirm translational applicability. Moreover, comprehensive physicochemical analyses (e.g., DSC, XRD, Raman spectroscopy) could provide deeper insights into drug‐polymer interactions that influence therapeutic efficacy. Finally, scaling up electrospinning processes and integrating multimodal imaging approaches for non‐invasive monitoring will be crucial steps toward clinical translation of these multifunctional nanofiber dressings.

## Author Contributions

V.P.N. electrospinning, manuscript writing, physicochemical analysis of nanofibers, B.B. in vivo wounding and monitoring, N.F. HF Ultrasound imaging, T.M. SEM imaging, camera imaging, image analysis, M.Gy. HF Ultrasound imaging analysis, M.F. camera imaging, image analysis, D.K. SEM imaging, image analysis, P.S. animal surgery, anesthesia, animal care, R.Cs‐K. animal breeding, supervision, O.B. histology, immunohistochemistry, M.R. manufacturing the histological slides, performing the IHC, photography of the slides, L.M. and I.A. tensile test and viscosity, R.H. contact angle measurement, zeta potential measurement, F.E. supervision, manuscript writing, and conceptualization, and A.F. conceptualization, supervision, and manuscript writing.

## Funding

Project no. 2020–1.1.5‐GYORSÍTÓSÁV‐2021‐00015 has been implemented with the support provided by the Ministry of Culture and Innovation of Hungary from the National Research, Development and Innovation Fund, financed under the 2020–1.1.5‐GYORSÍTÓSÁV funding scheme.

## Conflicts of Interest

The authors declare no conflicts of interest.

## Supporting information




**Supporting file 1**: adhm70839‐sup‐0001‐SuppMat.docx

**Supporting file 2**: adhm70839‐sup‐0002‐Complete Data.zip

## Data Availability

The data that support the findings of this study are available from the corresponding author upon reasonable request.
