# Supplementary material for: Hybrid Nanofibers for Multimodal Accelerated Wound Healing
Source: Adv Healthc Mater. 2026 Jan 28;15(15):e04029. doi: 10.1002/adhm.202504029 (PMC13088746; doi:10.1002/adhm.202504029)
Supplement: Supplementary file 1 — Supporting file 1: adhm70839‐sup‐0001‐SuppMat.docx [file ADHM-15-0-s002.docx]

**Hybrid nanofibers for multimodal accelerated wound healing**

Viraj P. Nirwan^1🕆^, Bence Bajusz^2^, Norbert Fabók^3^, Marina Rudan Dimlic^4^, Jelena Budimir^4^, Tshepang Mqatywa^2^, Miklós Gyöngy^2,3^, Márton Ferencz^2^, Dorottya Kocsis^2^, Olexandr Bondarenko^5^, Mariia Rolduhina^5^, Milena Lengyel^6^, Istvan Antal^6^, Rebecca Hengsbach^1^, Franciska Erdő^2🕆^ and Amir Fahmi^1^*^🕆^

^1^ Faculty of Technology and Bionics, Rhine-Waal University of Applied Science, Marie-Curie-Straβe 1, 47533 Kleve, Germany

^2^ Faculty of Information Technology and Bionics, Pázmány Péter Catholic University, Práter u. 50a., 1083 Budapest, Hungary

^3^ Dermus, Kanizsai utca 2-10 C/11, 1114 Budapest, Hungary

^4^ MedILS, University of Split, Meštrovićevo šetalište 45, 21000 Split, Croatia

^5^ Dnipro State Medical University, Volodymyra Vernadskoho str., 9, Dnipro, 49044, Ukraine

^6^Department of Pharmaceutics, Faculty of Pharmaceutical Sciences, Semmelweis University, Hőgyes Endre u. 7., Budapest, Hungary

E-Mail: virajpratap.nirwan@hochschule-rhein-waal.de, erdo.franciska@itk.ppke.hu, amir.fahmi@hochschule-rhein-waal.de

^🕆^: Viraj Pratap Nirwan, Amir Fahmi, and Franciska Erdő contributed equally to this article.

**Supplementary Information**

**Figure S1.** Wounding position of the SKH1 mice on the dorsal skin surface, laterally from the midline.

| 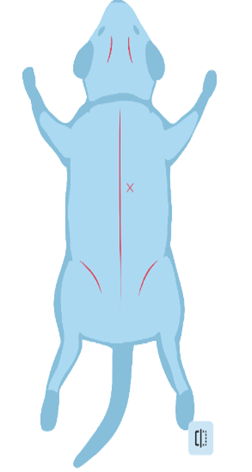 | 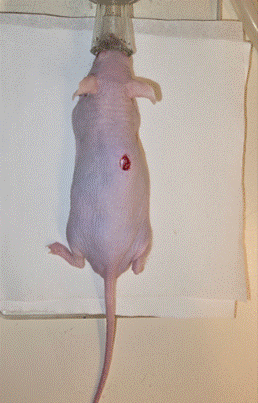 |
| --- | --- |

**Figure S2:** Zeta potential (A), conductivity (B) and electrophoretic mobility (C) of dispersed nanofibers, showing an increase for loaded samples. Error bars are partially in range of symbols. (n=3)
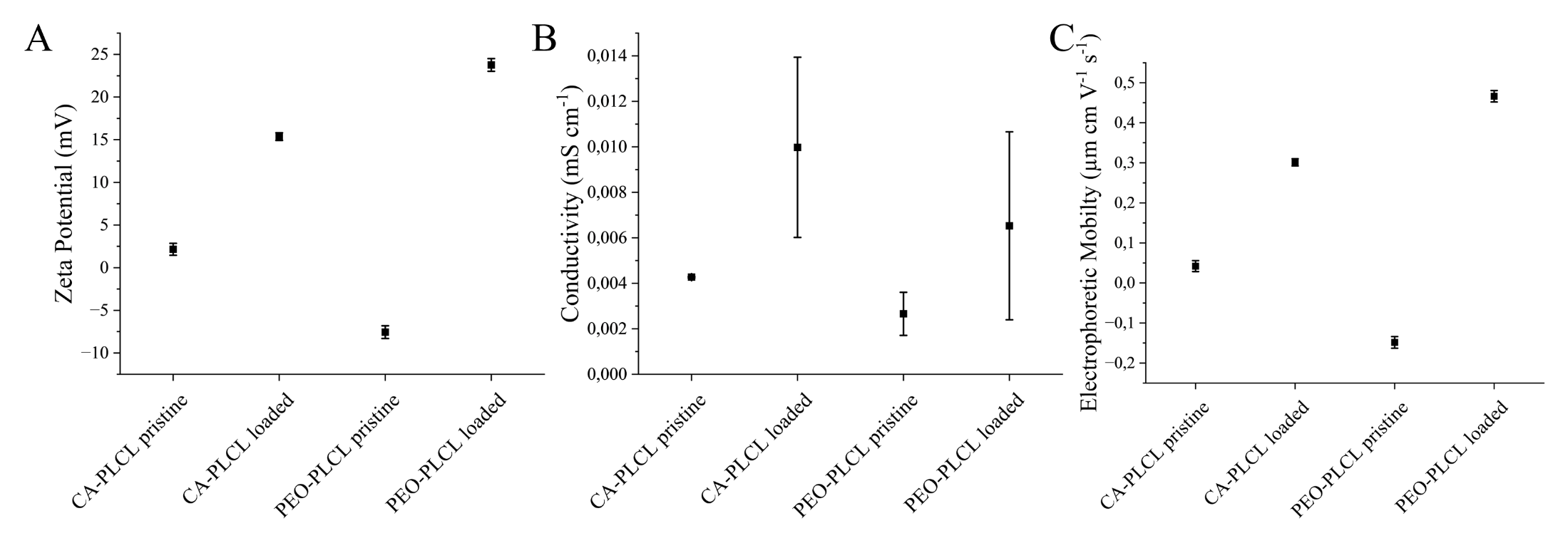


**Figure S3.**  Results of tensile strength measurement of nanofiber wound dressings

**A B**


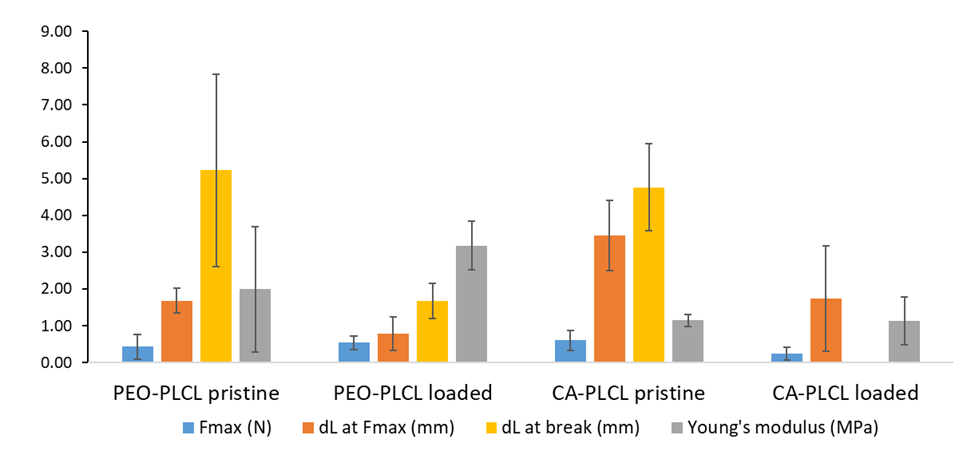

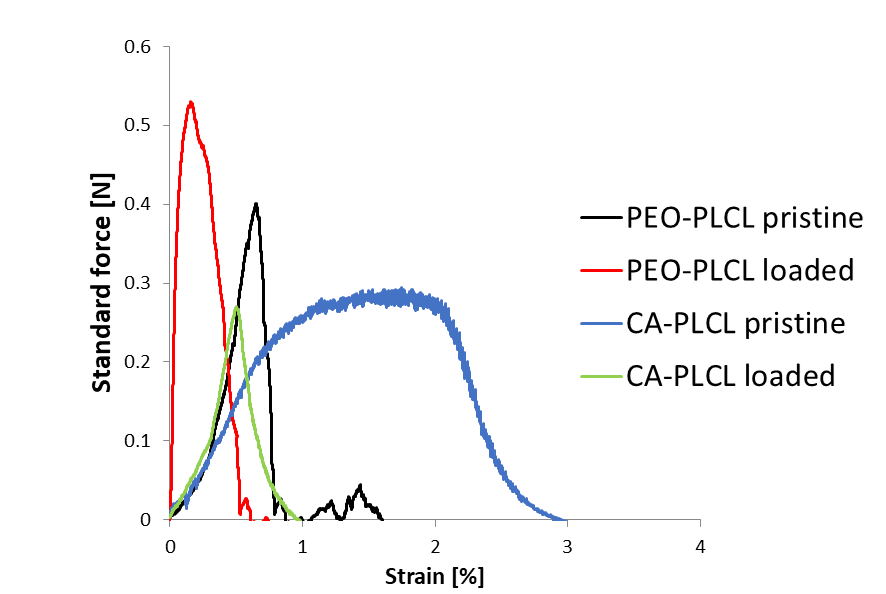


Panel (**A**) Maximum force (Fmax), length at maximum force (dL at Fmax) and length at break (dL) , Panel (**B**) Characteristic force-strain diagram of the tested samples. Means ± SD (n=5).

**Figure S4:** Swelling of electrospun nanofiber-based wound dressings (water uptake in %) (% of the dry weight). (n=3).


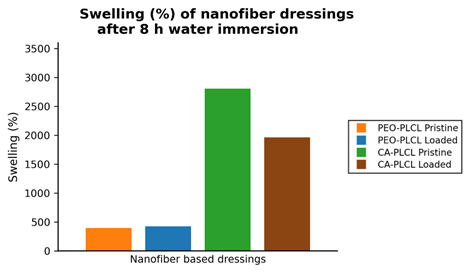

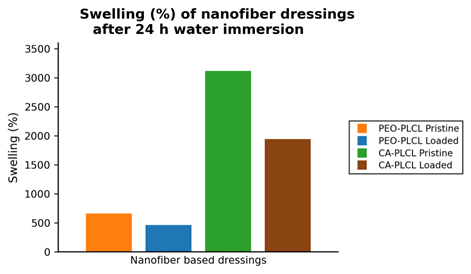


**Figure S5:** Cytotoxicity assessment of the nanofibers in primary dermal fibroblasts and human immortalized keratinocyte (HaCaT) cell lines. Means ± SD. (n=3)


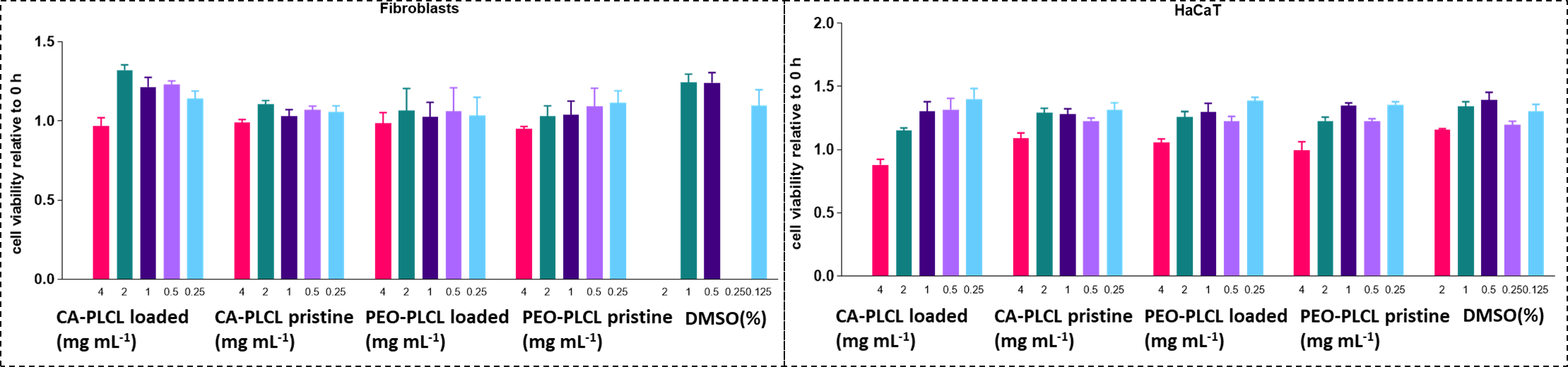


**Figure S6:** Cytotoxicity assessment of the active components dexamethasone and ascorbic acid in primary dermal fibroblasts and human immortalized keratinocyte (HaCaT) cell lines. Means ± SD. (n=3)


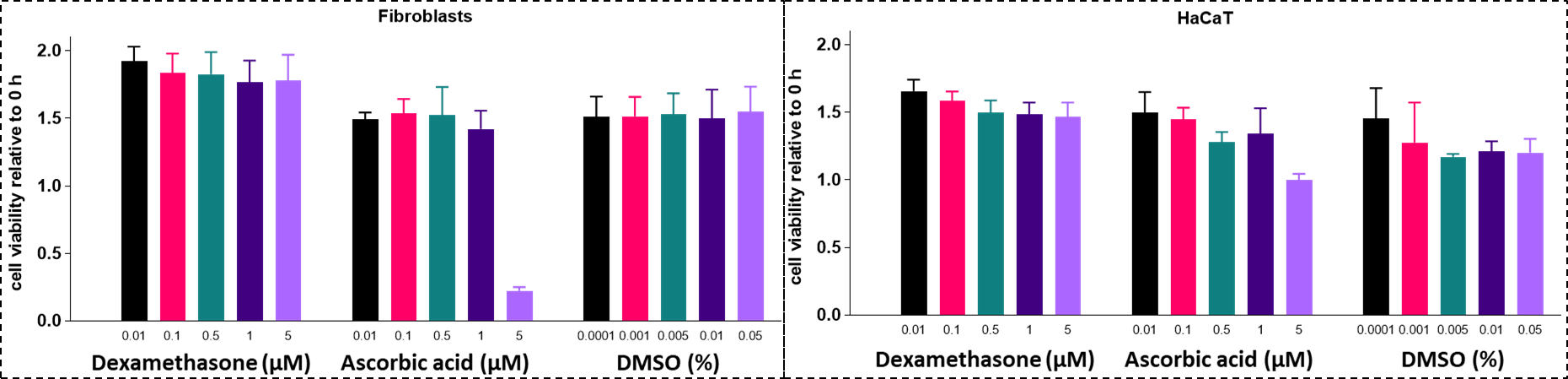


**Figure S7:** Photographic chronicle of wound healing progress in response to various scaffolds and compared to the control.

| CA-PLCL pristine 5 | CA-PLCL pristine 4 | CA-PLCL pristine 3 | CA-PLCL pristine 2 | CA-PLCL pristine 1 | Control 5 | Control 4 | Control 3 | Control 2 | Control 1 |  |
| --- | --- | --- | --- | --- | --- | --- | --- | --- | --- | --- |
| 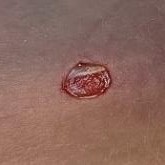 | 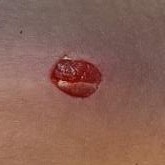 | 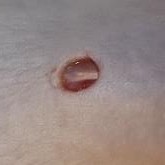 | 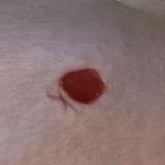 | 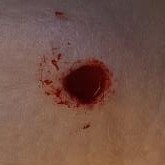 | 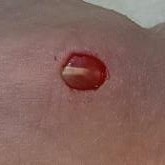 | 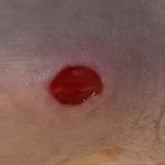 | 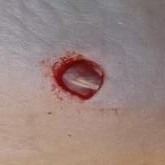 | 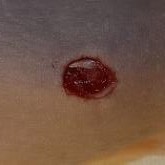 | 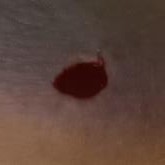 | Day 1 |
| 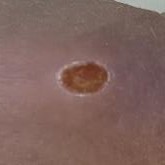 | 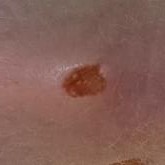 | 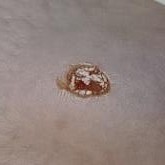 | 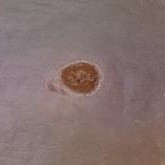 | 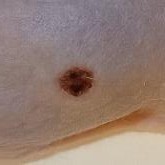 | 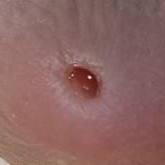 | 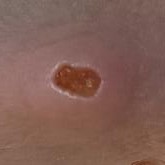 | 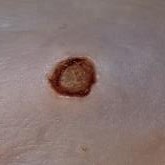 | 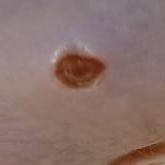 | 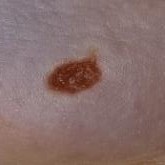 | Day 3 |
| 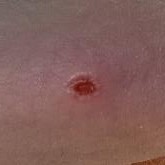 | 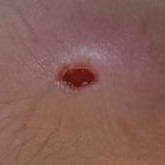 | 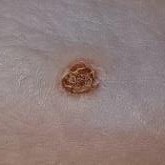 | 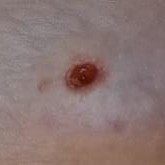 | 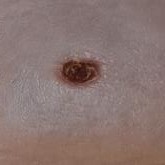 | 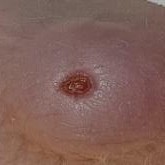 | 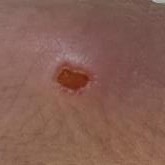 | 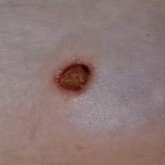 | 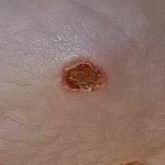 | 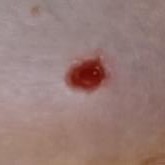 | Day 5 |
| 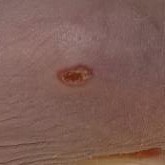 | 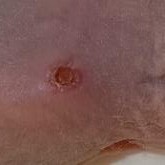 | 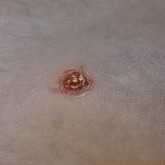 | 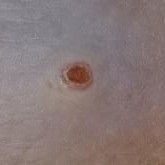 | 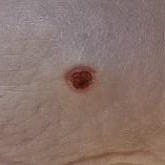 | 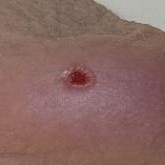 | 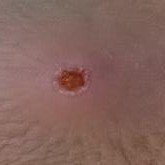 | 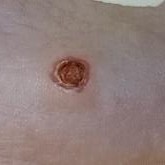 | 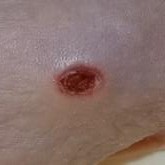 | 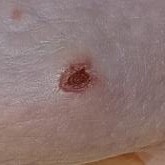 | Day 7 |
| 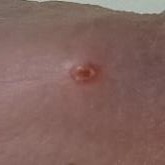 | 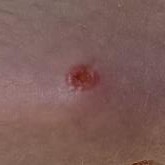 | 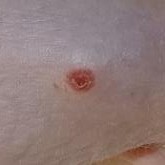 | 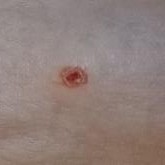 | 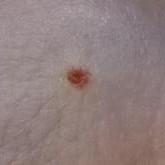 | 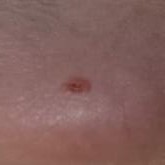 | 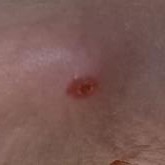 | 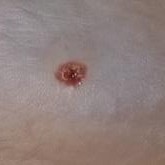 | 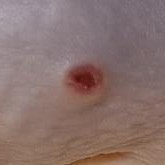 | 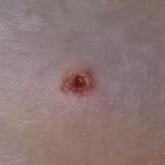 | Day 9 |
| 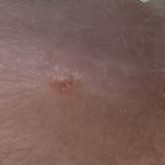 | 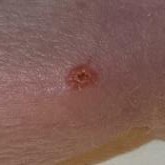 | 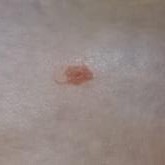 | 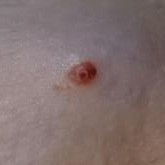 | 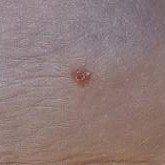 | 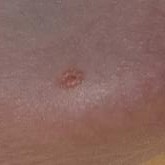 | 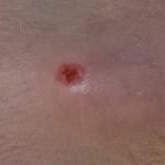 | 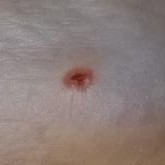 | 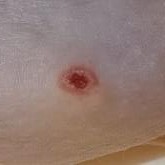 | 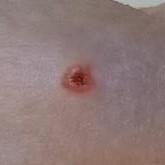 | Day 11 |
| 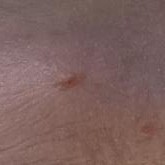 | 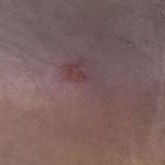 | 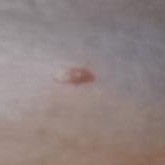 | 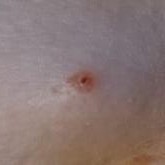 | 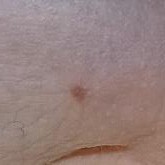 | 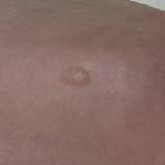 | 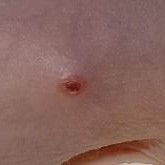 | 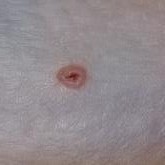 | 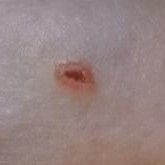 | 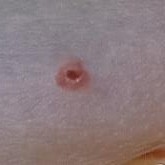 | Day 13 |
| 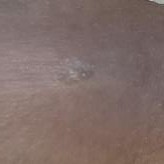 | 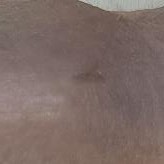 | 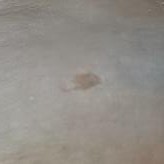 | 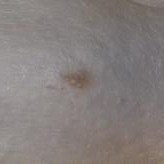 | 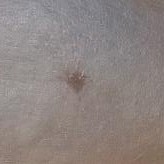 | 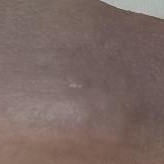 | 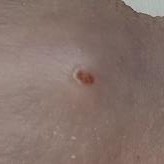 | 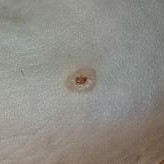 | 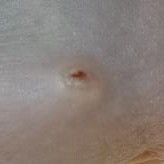 | 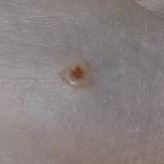 | Day 15 |

| PEO-PLCL pristine 5 | PEO-PLCL pristine 4 | PEO-PLCL pristine 3 | PEO-PLCL pristine 2 | PEO-PLCL pristine 1 | CA-PLCL loaded 5 | CA-PLCL loaded 4 | CA-PLCL loaded 3 | CA-PLCL loaded 2 | CA-PLCL loaded 1 |  |
| --- | --- | --- | --- | --- | --- | --- | --- | --- | --- | --- |
| 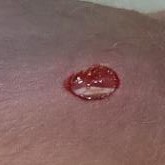 | 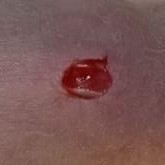 | 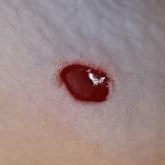 | 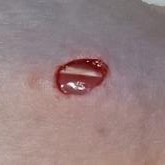 | 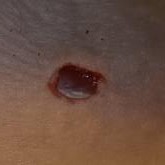 | 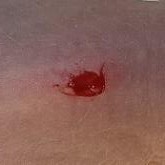 | 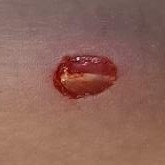 | 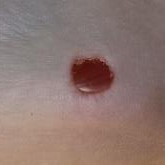 | 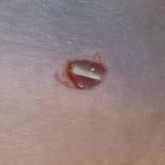 | 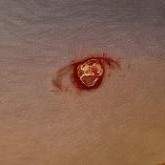 | Day 1 |
| 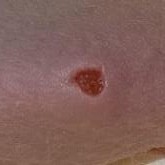 |  |  |  |  |  |  |  |  |  | Day 3 |
|  |  |  |  |  |  |  |  |  |  | Day 5 |
|  |  |  |  |  |  |  |  |  |  | Day 7 |
|  |  |  |  |  |  |  |  |  |  | Day 9 |
|  |  |  |  |  |  |  |  |  |  | Day 11 |
|  |  |  |  |  |  |  |  |  |  | Day 13 |
|  |  |  |  |  |  |  |  |  |  | Day 15 |

| PEO-PLCL loaded 4 | PEO-PLCL loaded 3 | PEO-PLCL loaded 2 | PEO-PLCL loaded 1 |  |
| --- | --- | --- | --- | --- |
|  |  |  |  | Day 1 |
|  |  |  |  | Day 3 |
|  |  |  |  | Day 5 |
|  |  |  |  | Day 7 |
|  |  |  |  | Day 9 |
|  |  |  |  | Day 11 |
|  |  |  |  | Day 13 |
|  |  |  |  | Day 15 |

**Table S1.** Schedule for monitoring the wound closure by camera imaging and optically assisted high-frequency ultrasound imaging.

| Days | 1 | 3 | 5 | 7 | 9 | 11 | 13 | 15 |
| --- | --- | --- | --- | --- | --- | --- | --- | --- |
| Camera image | x | x | x | x | x | x | x | x |
| Ultrasound  image | x | x | x |  | x | x | x | x |

The healing area was measured according to the previously described protocol on a schedule.

**Table S2.** Numeric data of the wound closure process as it was measured by HF Ultrasound imaging.

| **Days / mm** | **1** | **3** | **5** | **7** | **9** | **11** | **13** | **15** | **Comments** |
| --- | --- | --- | --- | --- | --- | --- | --- | --- | --- |
| Ultrasound open wound measurement | 4.75 | 2.84 | 2.10 |  |  |  |  |  | The open wound disappeared by day 7, and scar formation started. |
| Ultrasound total wound measurement | 4.75 | 5.13 | 4.51 |  | 3.38 | 4.56 | 4.08 | 3.99 | There is a slight variation (day 9) in the total wound diameter due to the contraction of the skin around the wound. |
| Ultrasound scar (remodeling phase) measurement |  |  | 3.40 |  | 2.15 | 2.36 | 1.93 | 1.59 | The scab that covers the wound (formed between days 7-8) shows an almost constant decrease in size. |
