# Supplementary material for: Hybrid Nanofibers for Multimodal Accelerated Wound Healing
Source: Adv Healthc Mater. 2026 Jan 28;15(15):e04029. doi: 10.1002/adhm.202504029 (PMC13088746; doi:10.1002/adhm.202504029)
Supplement: Supplementary file 2 — Supporting file 2: adhm70839‐sup‐0002‐Complete Data.zip [file ADHM-15-0-s001.zip › Complete Data/Cell cuture studies/EXP_Nanofibers_Cell lines_MTT summary 2025 may.pdf]

Experiment

Nanofibers (loaded and pristine) application to cell lines

Collaboration with professor Franciska Erdő (Pázmány Péter Catholic University)

Experiment planned and performed at MedILS, UNIST

by Marina Rudan Dimlić and Anja Golemac Lipovac

Experimental set up:

- 12.05.2025
  - **seeding** cells in 48-well plate
    - used cell lines were **human dermal fibroblasts** (28FBP p=6) and **immortalized keratinocytes** (HaCaT)
  - cells were grown in the incubator at 37°C (5% CO_2) for 2 days (to reach around 80% confluency)

- 15.05.2025
  - making **scratch** (using a tips) and **applying nanofibers** to the cells
  - incubation with nanofibers for **4H** and **24H** at 37°C (5% CO_2)
  - after 4H incubation removing nanofibers from wells and performing **MTT assay**

- 16.05.2025
  - after 24H incubation removing nanofibers from wells and performing **MTT assay**

Samples (nanofibers)

1. hydrophobic loaded
2. hydrophobic pristine
3. hydrophilic loaded
4. hydrophilic pristine

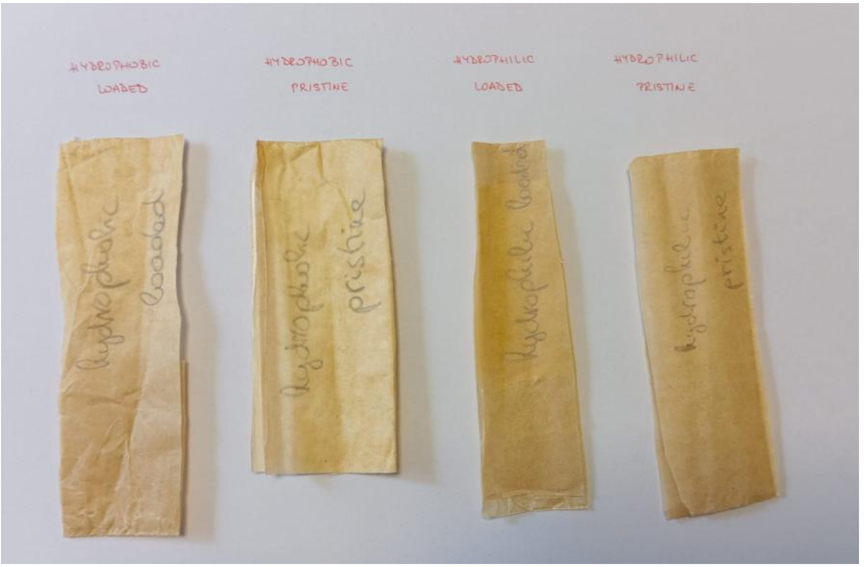

We cut each sample into 24 equal parts of a size corresponding to the size of a well in a 48-well plate

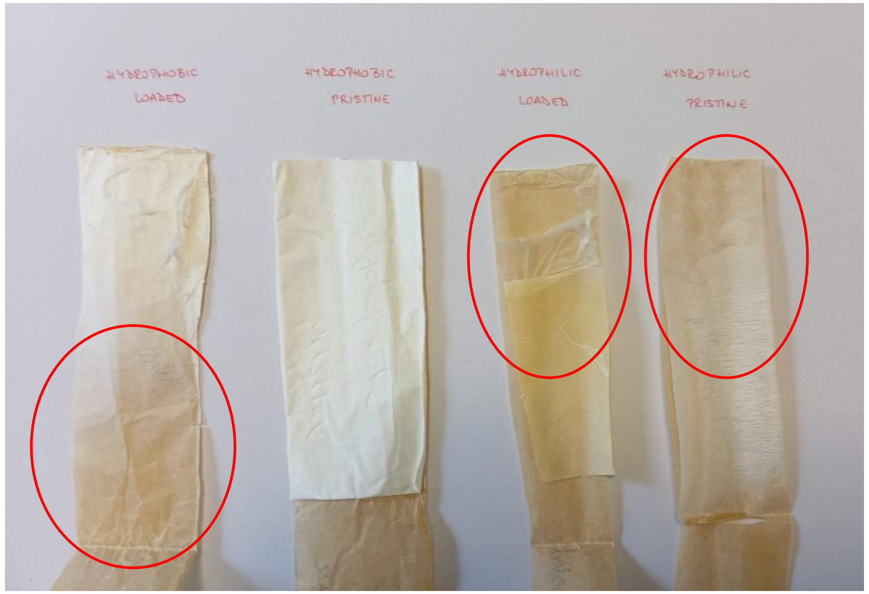

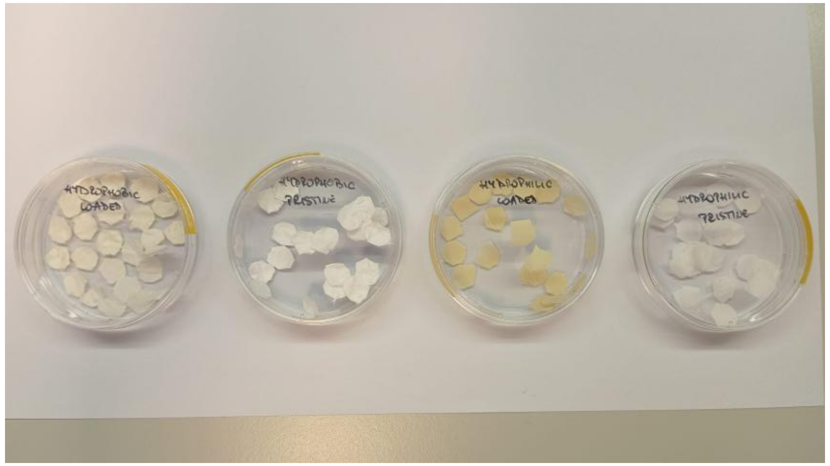

Note for marked samples

Some samples appeared to be blended ("merged") into the paper they were placed in

Nanofibers application on cells to achieve direct contact

- some nanofibers **didn't attach** to the cells (shown on the picture)
- nanofibers that attach to the cells caused **losing those cells when removing nanofibers from wells** (shown on the picture on next slide)
- **Note**: in excel files we marked with + wells in which nanofibers were attached

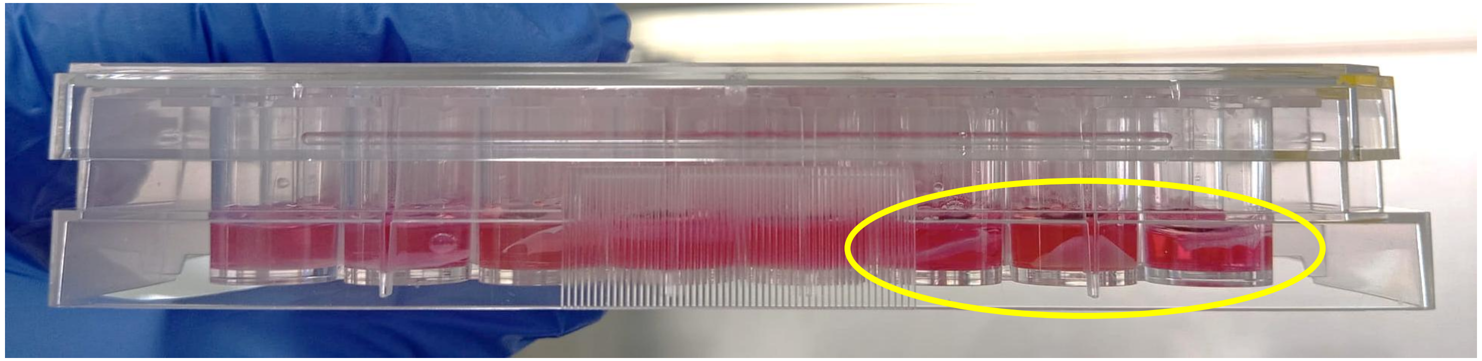

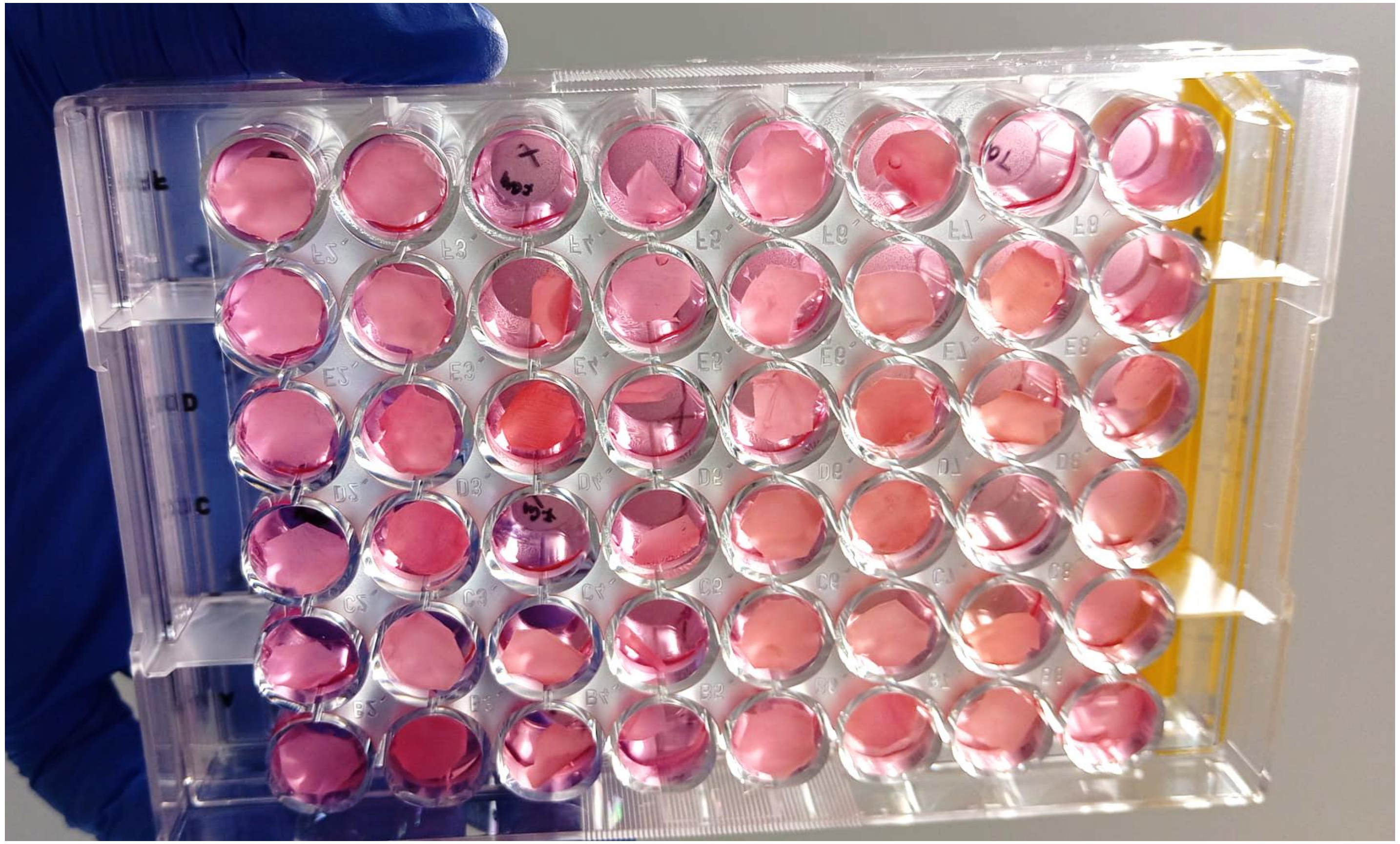

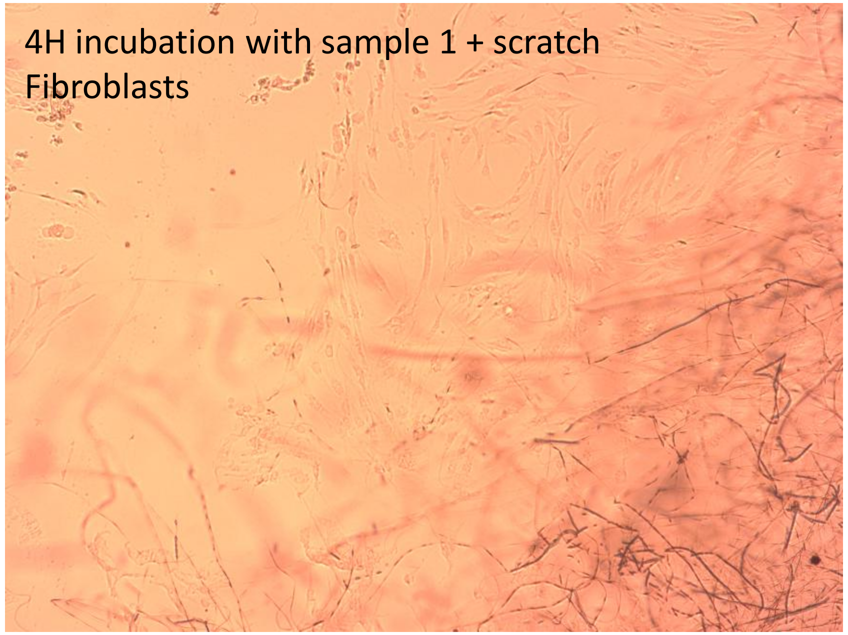
4H incubation with sample 1 + scratch
Fibroblasts

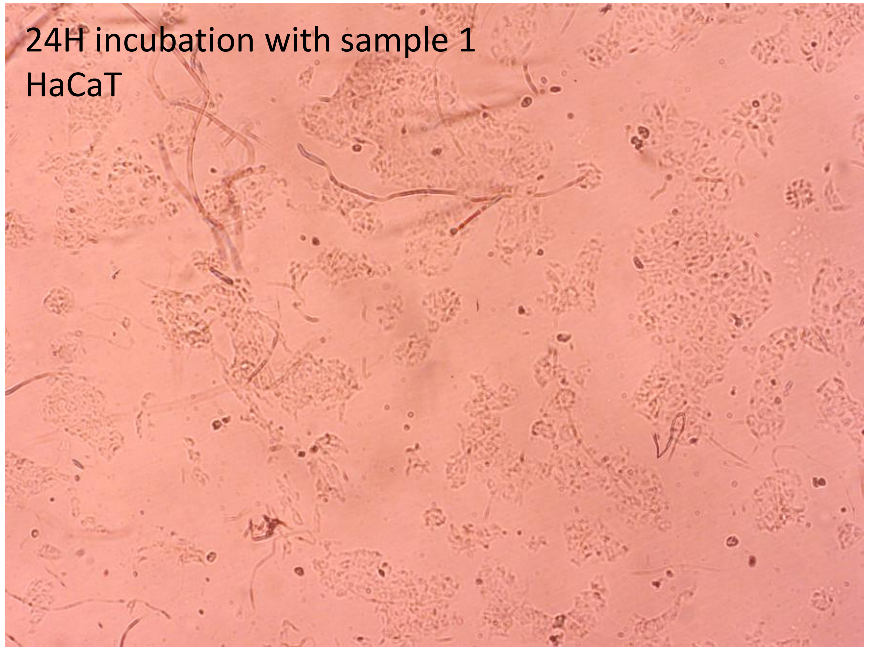
24H incubation with sample 1
HaCaT

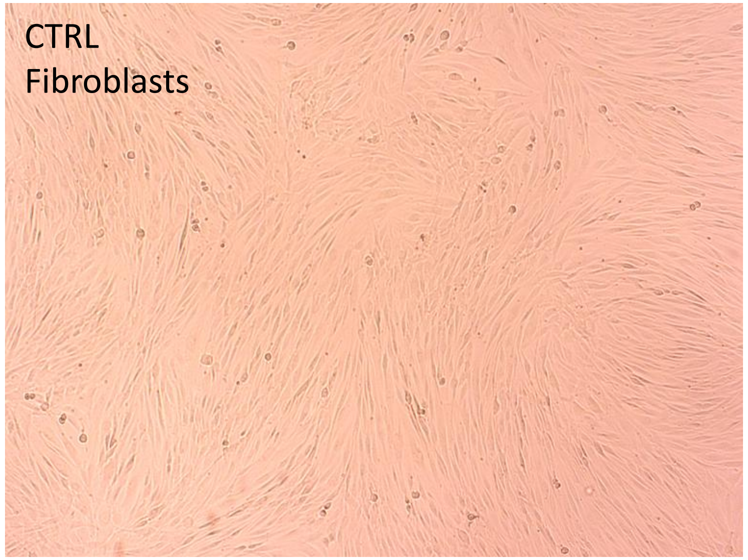
CTRL
Fibroblasts

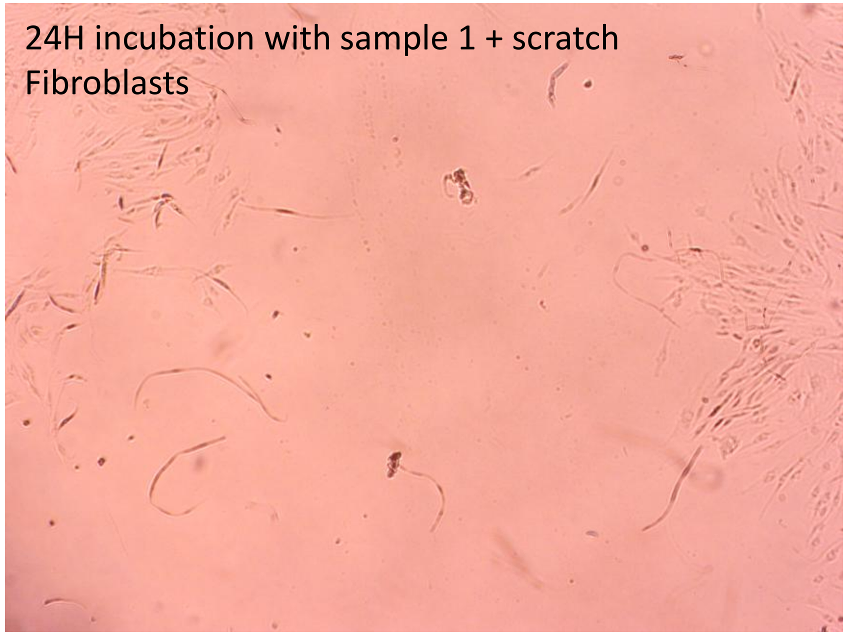
24H incubation with sample 1 + scratch
Fibroblasts

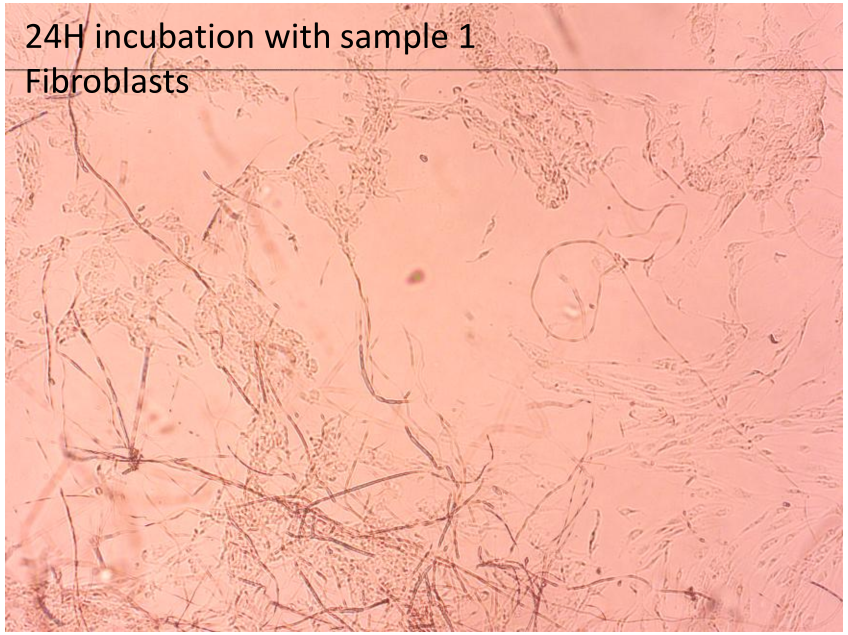
24H incubation with sample 1
Fibroblasts

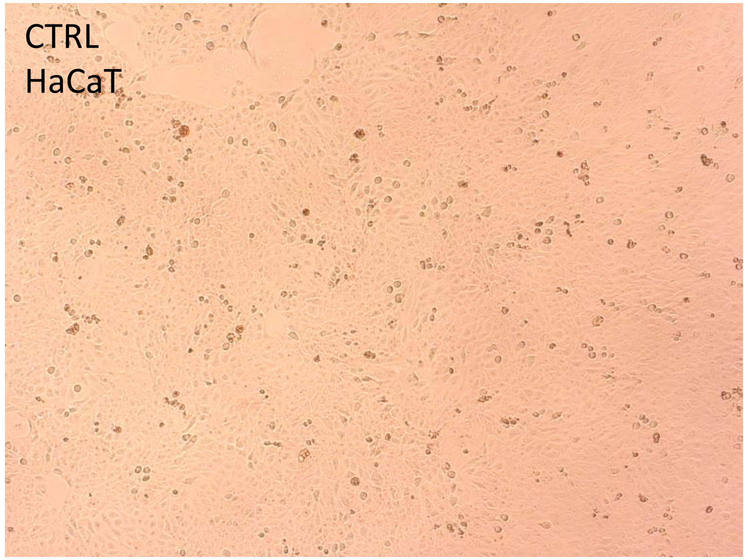
CTRL
HaCaT

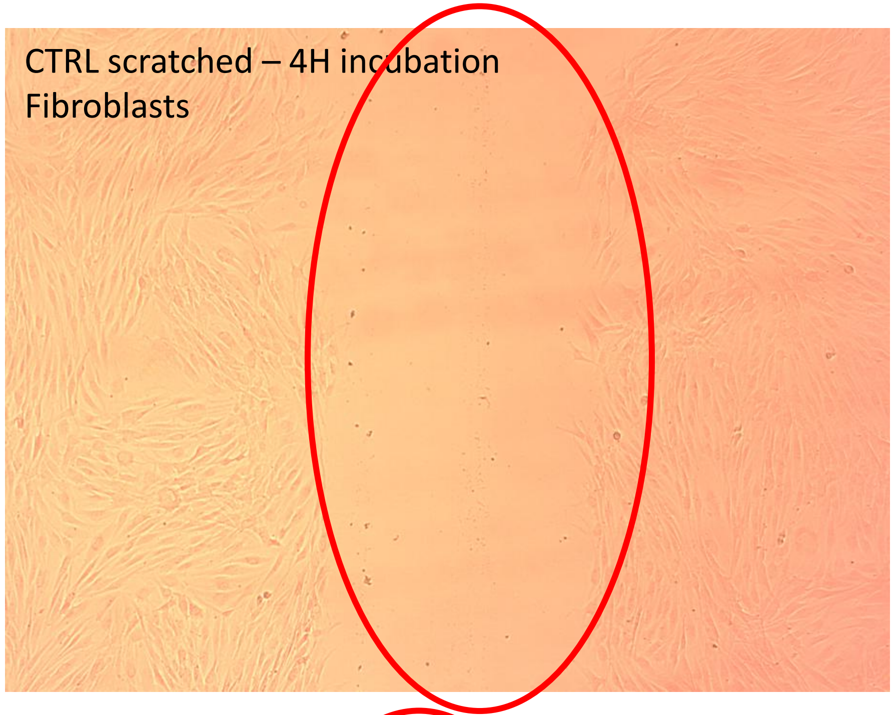
CTRL scratched – 4H incubation
Fibroblasts

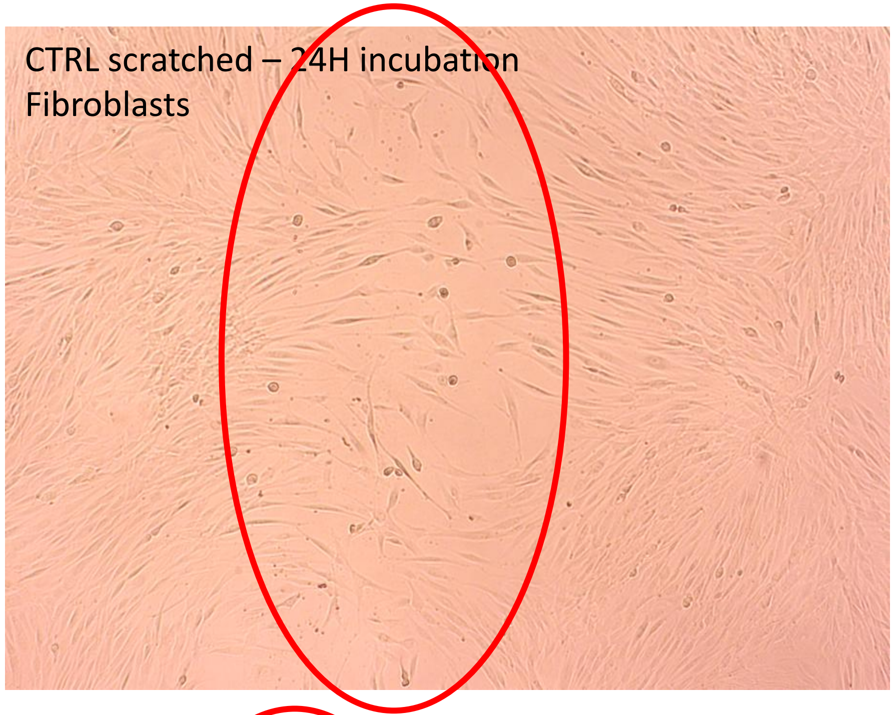
CTRL scratched – 24H incubation
Fibroblasts

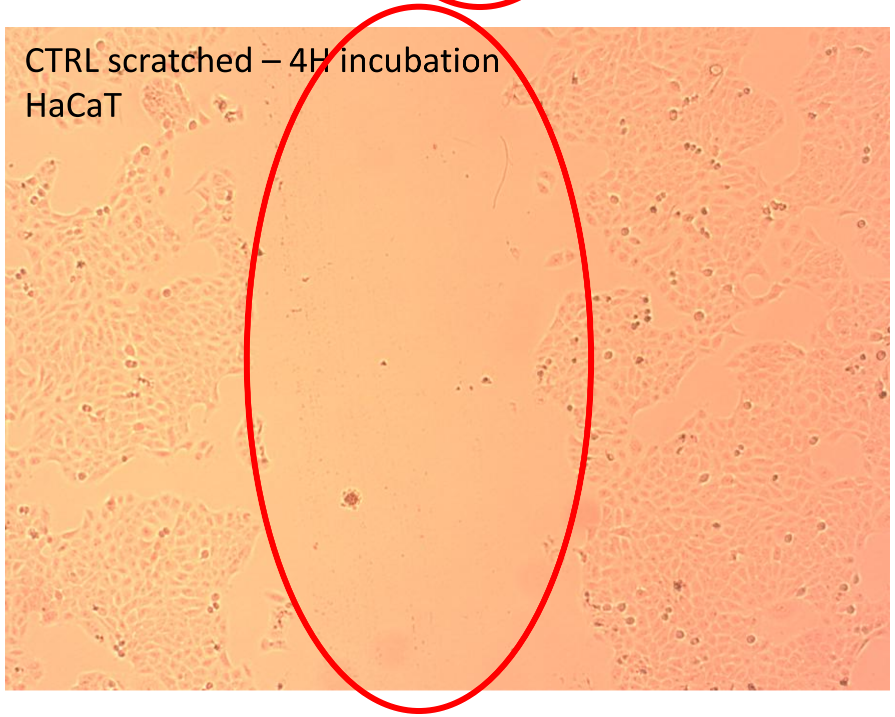
CTRL scratched – 4H incubation
HaCaT

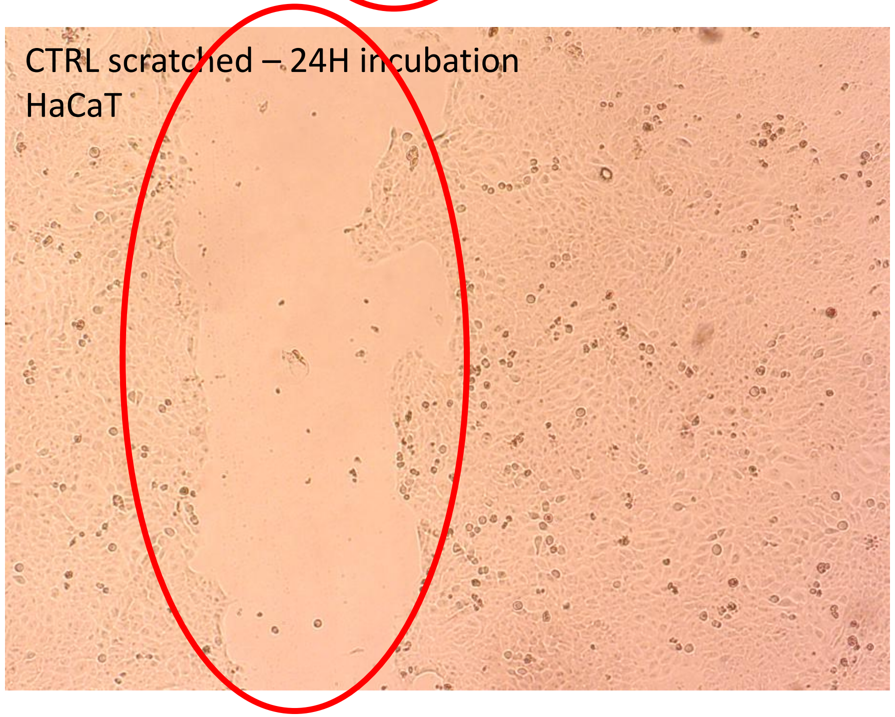
CTRL scratched – 24H incubation
HaCaT

MTT assay

- The MTT assay is a colorimetric assay used to assess cell metabolic activity as an indicator of cell viability, proliferation, and cytotoxicity
- It is based on the ability of mitochondrial enzymes in living cells to reduce the yellow tetrazolium salt (MTT) to an insoluble purple formazan product
- The amount of formazan produced is directly proportional to the number of metabolically active cells, and it can be quantified by measuring absorbance with a spectrophotometer (595 nm)

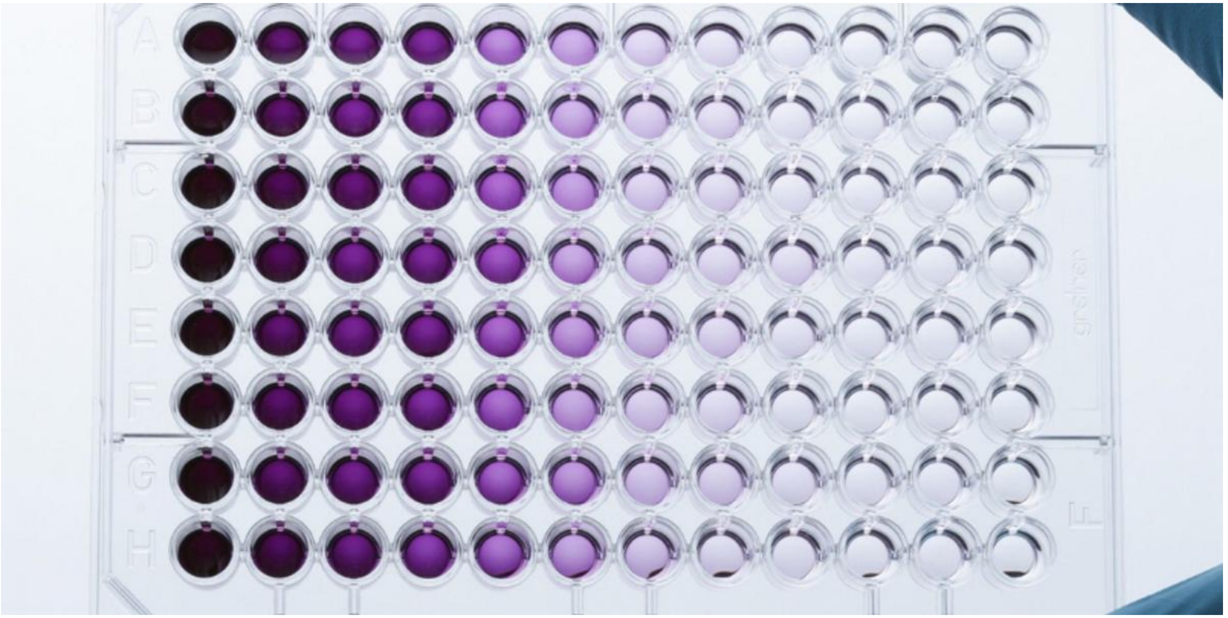

Live cells Dead cells

Conclusions

- since some nanofibers didn’t attach to the cells or they damaged cells when attached to it results obtained in MTT assay are not reliable

Options in order to predict cells damage in direct contact with nanofibers

1. **well inserts**
    - fibroblasts or HaCaT cells would be seeded in the insert, while nanofibers (either loaded or pristine) would be placed beneath it, in the space between the insert and the well. In this setup, only the membrane separates the cells from the nanofibers
2. **dissolving nanofibers** in DMSO or water (depends on the solubility of the component loaded into nanofibers) to “extract” components and then treat cells with these “extracts”
3. **testing pure components** that are loaded into nanofibers
