# Supplementary material for: Hybrid Nanofibers for Multimodal Accelerated Wound Healing
Source: Adv Healthc Mater. 2026 Jan 28;15(15):e04029. doi: 10.1002/adhm.202504029 (PMC13088746; doi:10.1002/adhm.202504029)
Supplement: Supplementary file 2 — Supporting file 2: adhm70839‐sup‐0002‐Complete Data.zip [file ADHM-15-0-s001.zip › Complete Data/HF-US imaging + photos/Dermus_Preclinical_Mouse_Wound_Healing_20241216_compressed (1).docx]

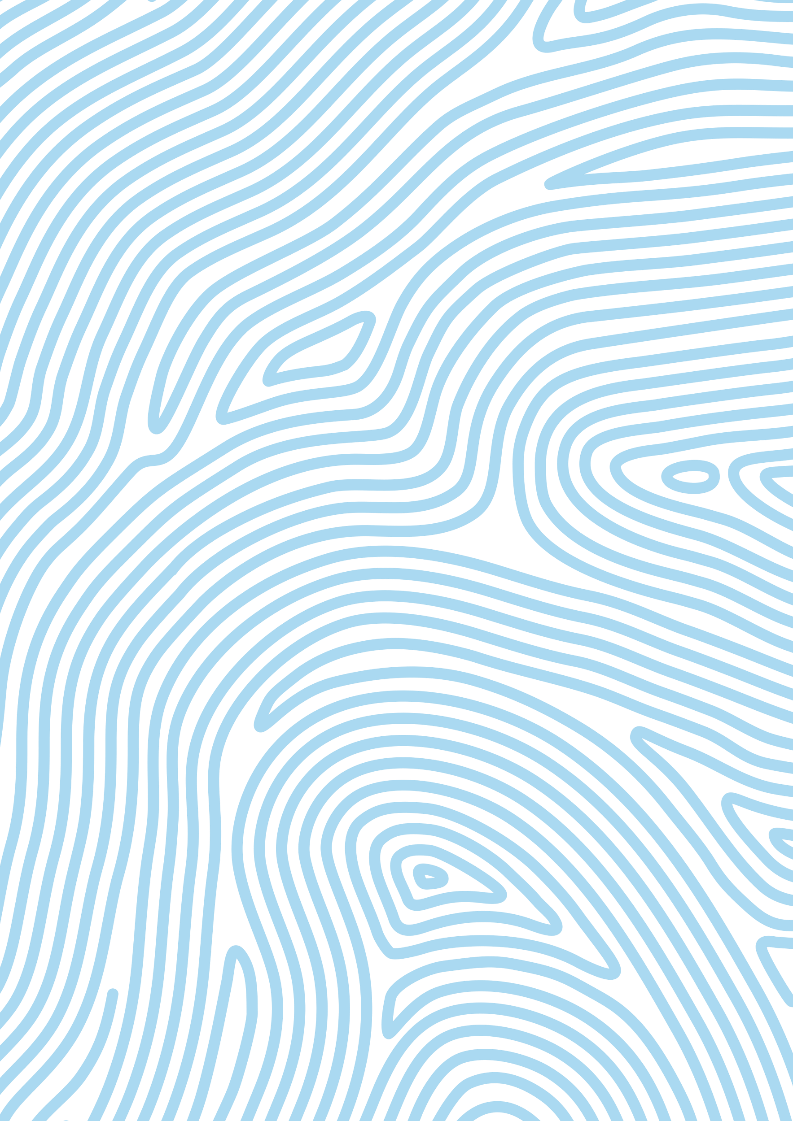


Mouse Wound Healing

Internal study

[www.dermusvision.com](http://www.dermusvision.com)

Introduction

In this internal study, we focused on the optical and ultrasound monitoring of a mouse wound healing model. We aimed to compare day to day changes during a 15-day period using our dermoscopy-guided high-frequency ultrasound (DG-HFUS) device (Dermus SkinScanner-U). The wound was created by a 5.00mm puncture biopsy tool.

The conclusion of this study is that our DG-HFUS device was effective in monitoring the healing process both on the surface and the depth of the mouse skin.

| 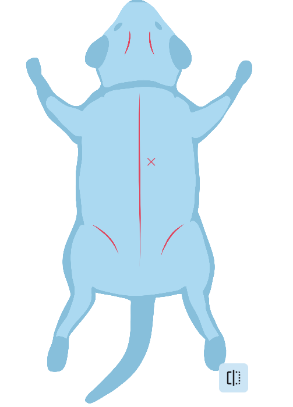 | 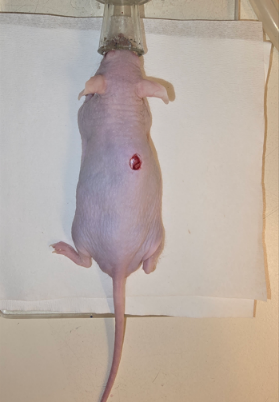 |
| --- | --- |

Day 1 (18 NOV 2024)

| 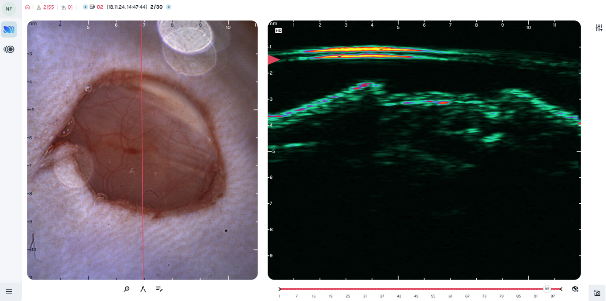 |
| --- |
| 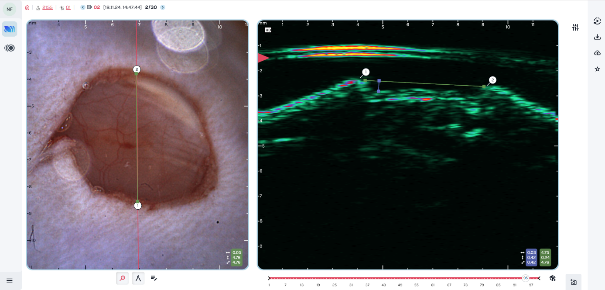 |

- Initial measurement was taken right after the puncture biopsy (5.00mm needle)
- Total diameter of the wound shows perfect correlation between the optical and ultrasound measurements
- Haemostasis phase has started
- Thickness of the removed skin was around 0.50mm (initial wound depth).

Day 3 (20 NOV 2024)

| 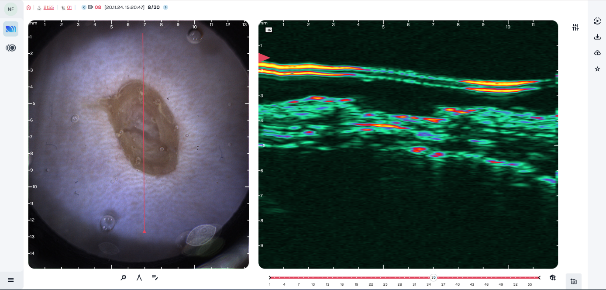 |
| --- |
| 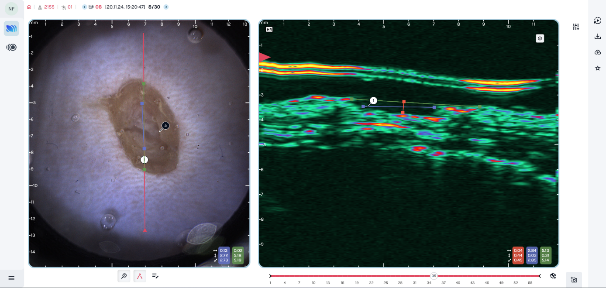 |

- Total wound diameter is still very close to the original (5.13mm)
- Tissue regeneration started. Scab builds up at the side of the wound
- Inflammatory phase
- Scar has not been formed completely yet, the bottom of the wound is still visible (both on the ultrasound and optical image, 0.44mm).

Day 5 (22 NOV 2024)

| 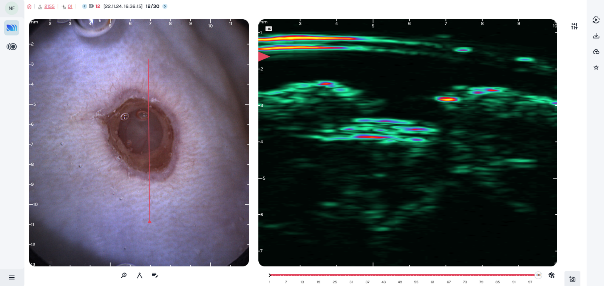 |
| --- |
| 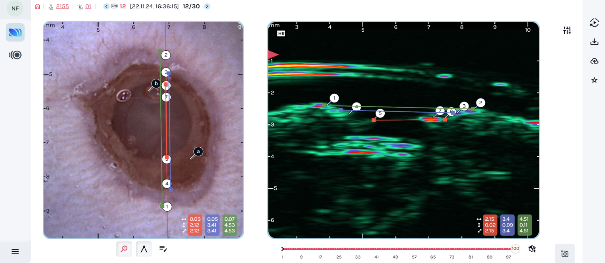 |

- “1” & “2” mark the original wound size
- “3” & “4” point to the outer borders of the building scab
- “5” & “6” flag the open wound
- “7” & “b” show a bubble which causes a slight hyper-echogenic reflection
- “a” indicates the building scab
- At “1” & “2” the ultrasound image shows two bumps at the edges which is a clear indication of inflammation
- The red circle indicates an area with higher echogenicity which might be the early sign of fibroblast proliferation
- Due to the hydration effect of the ultrasound gel a slightly clearer wound is observable compared to the previous measurement.

Day 7 (24 NOV 2024)

- There was no measurement.

Day 9 (26 NOV 2024)

| 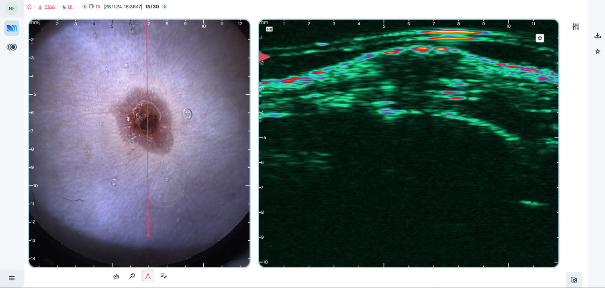 |
| --- |
| 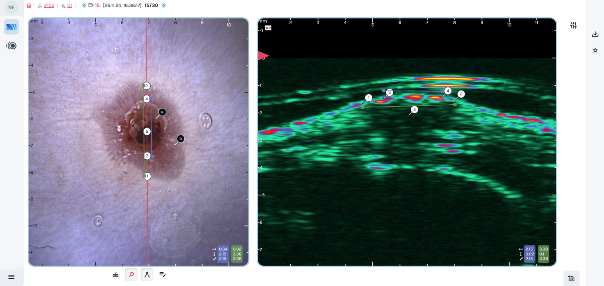 |

- The wound is completely covered with the scab
- The thickened skin layer has a higher echogenicity and produces a light acoustic shadow due to the scab’s keratotic surface (“3” and “4”)
- The diameter of the wound is slightly decreased due to the contraction of the skin
- “5” shows the inflamed area under the scab
- The wound is in the proliferation phase now
- Based on the echogenicity difference between the areas marked with “1” and “2” and the areas marked with "3” and “4” the different healing phases could be clearly distinguished on both images.

Day 11 (28 NOV 2024)

| 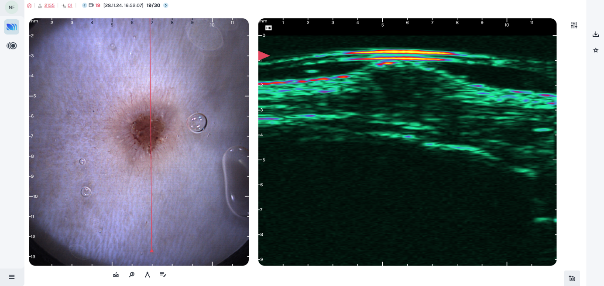 |
| --- |
| 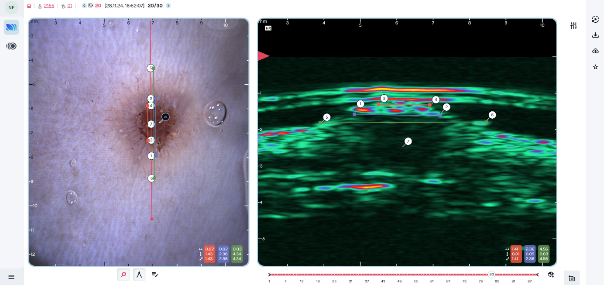 |

- The skin is releasing its stiffness around the wound and it is releasing back itself to the original size (biopsy day diameter )
- The scab’s thickness has increased (clearly visible on the ultrasound images)
- The volume of the inflamed part significantly increased (“7”)
- The outer “ring” of the wound is in regeneration stage while the inner part (center) is in proliferation stage
- We can observe three concentric circles:
  - Between ”3” & “4” is the thickest part of the wound with a relatively large scab
  - Between ”1” & “2” is still a relatively thick, but much less keratotic part (lower echogenicity)
  - Between “6” & “5” is the original biopsy’s border, already in regeneration phase.

Day 13 (30 NOV 2024)

| 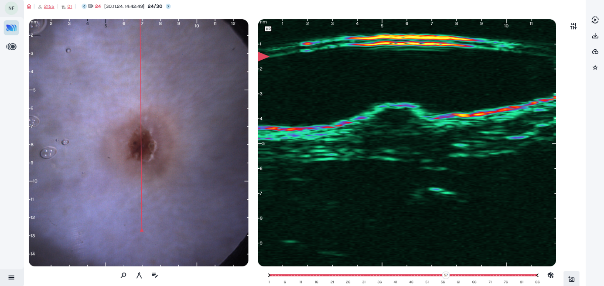 |
| --- |
| 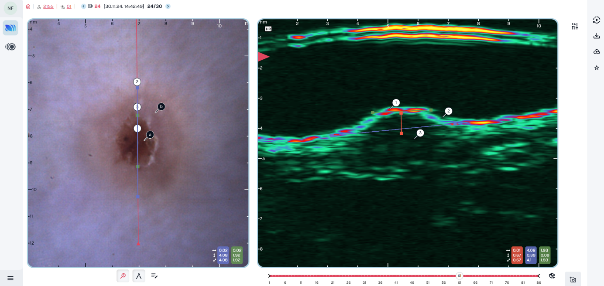 |

- Inflammation is shrinking
- Elevated, inflamed wound is 0.67mm only
- ”b” shows that the edges of the scab have been divided from the surface and it is about to fall off
- The top of the wound, the keratotic part is getting smaller.

Day 15 (02 DEC 2024)

| 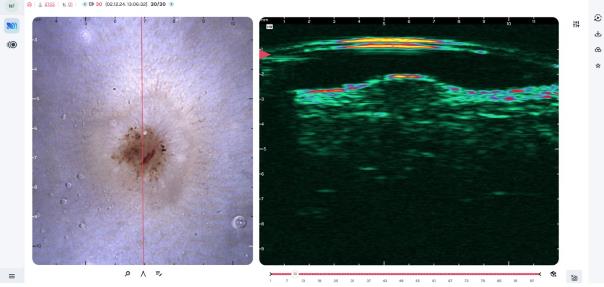 |
| --- |
| 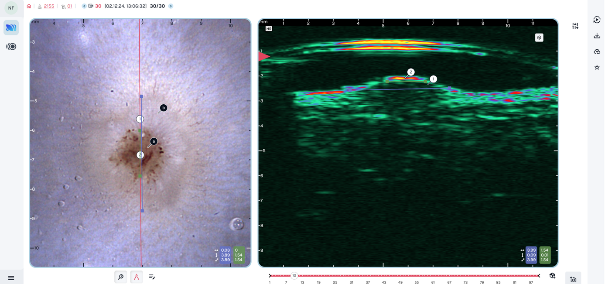 |

- Last day of measurement
- The scab fell off
- Regeneration phase
- Inflammation disappeared almost completely
- Only a small acoustic shadow is generated by the top of the wound
- Original biopsy borderline is still visible.

Summary

| 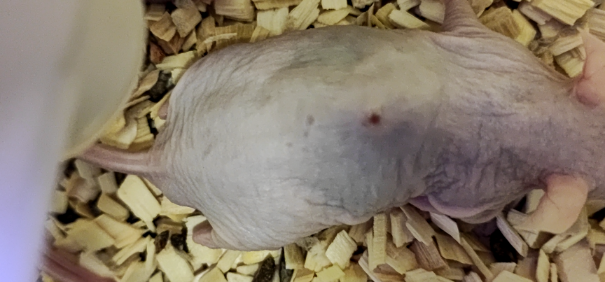 |
| --- |

| Days / mm | 1 | 3 | 5 | 7 | 9 | 11 | 13 | 15 | Comments |
| --- | --- | --- | --- | --- | --- | --- | --- | --- | --- |
| Ultrasound open wound measurement | 4.75 | 2.84 | 2.10 |  |  |  |  |  | The open wound disappeared by day 7 and scar formation started |
| Ultrasound total wound measurement | 4.75 | 5.13 | 4.51 |  | 3.38 | 4.56 | 4.08 | 3.99 | There is a slight variation (day 9 ) in the total wound diameter due to contraction of the skin around the wound |
| Ultrasound scar (remodeling phase) measurement |  |  | 3.40 |  | 2.15 | 2.36 | 1.93 | 1.59 | The scab which covers the wound (formed between day 7-8) shows an almost constant decrease in size |
