# Supplementary material for: Hybrid Nanofibers for Multimodal Accelerated Wound Healing
Source: Adv Healthc Mater. 2026 Jan 28;15(15):e04029. doi: 10.1002/adhm.202504029 (PMC13088746; doi:10.1002/adhm.202504029)
Supplement: Supplementary file 2 — Supporting file 2: adhm70839‐sup‐0002‐Complete Data.zip [file ADHM-15-0-s001.zip › Complete Data/Tensile strength - viscosity/summary (1).docx]

**Rheological characterization**

Rheological measurements were performed by Kinexus Pro Rheometer (Malvern Instruments Ltd, UK) registering the data with rSpace for Kinexus Pro 1.3 software. Samples were characterized using a 50 mm-cone and plate geometry where the gap for sample placement was 0.03 mm. Rotational measurements of formulations were determined at 25 ℃ controlled with an accuracy of ±0.1 ℃ by Peltier system of the instrument. In all measurements (3 parallels) a cylindrical cover made of stainless steel was placed over the samples, to create a closed, saturated volume around the sample and to prevent evaporation of the samples.

The viscosity curves were registered between 0.1-100 1/s shear rate with a setting of 10 samples per decade.

The samples were dissolved in R-acetone and distilled water (2:1) to reach a 5%w/w solution with in an ultrasonic bath (Branson.

The viscosity measurements indicate that the hydrophilic PEO samples (both pristine and loaded) were soluble in the solvent mixture and the viscosity measurement showed the increase of the viscosity for the API-containing samples. As the hydrophobic samples were only dispersible in the solvent mixture, the viscosity curves were not smooth. However, the tendency is clearly visible: the API containing samples (CA-PLCL loaded) have a lower viscosity than the pristine samples.


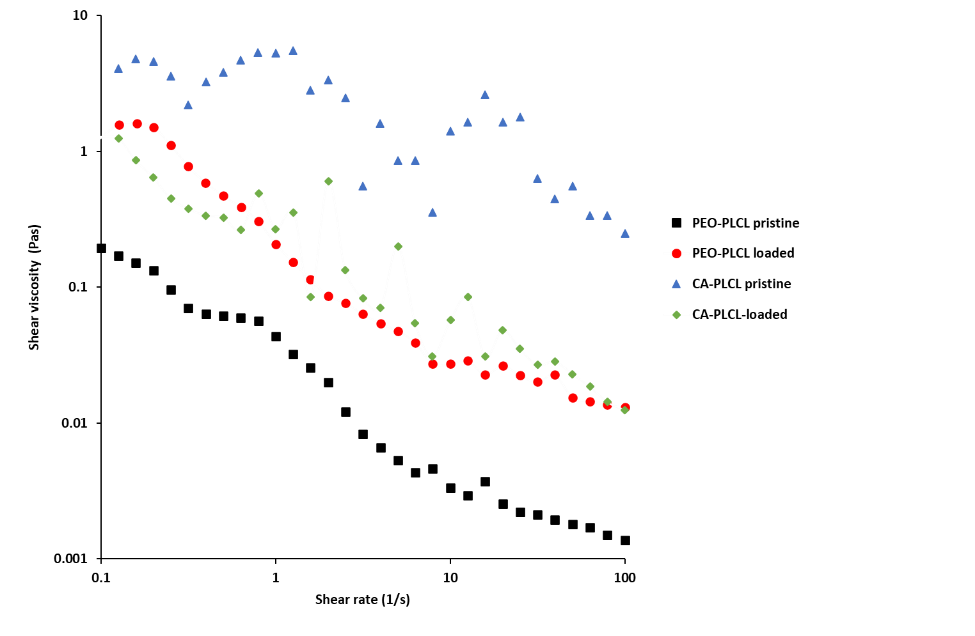


**Mechanical characterisation: tensile strength**

Tensile strength measurements were performed by Zwicki-Line Z005 table top universal testing machine (ZwickRoell GmbH & Co. KG, Ulm, Germany) equipped with a 5 kN load cell. The samples (length x width: 20 × 10 mm) were kept at room temperature in closed containers for 24 hours prior to the measurements, then fixed in a pincer grip and tested at a crosshead speed of 0.1 mm/min. The initial distance between the grips was set 10 mm. The stress-strain curves were recorded using TestXpert II software (ZwickRoell GmbH & Co. KG) until sample rupture. Young’s modulus was calculated from the slope of the linear elastic region in the stress-strain curve, where stress (σ) is proportional to strain (ε) per Hooke's law:

$$E=\frac{\sigma}{\varepsilon}$$

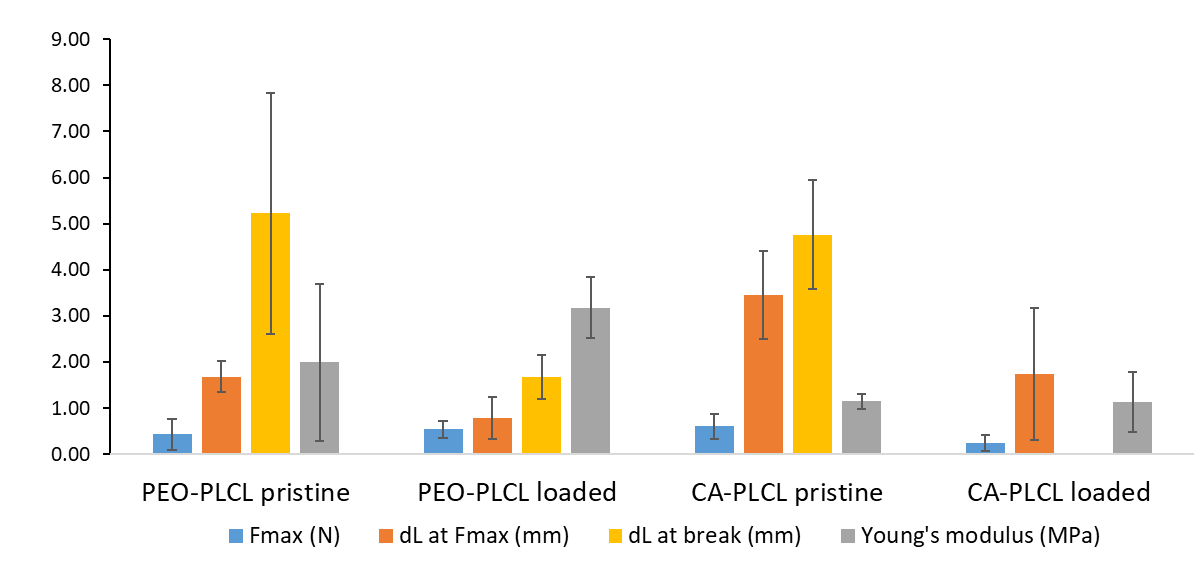


Figure Results of tensile strength measurement of nanofiber wound dressings (n=5)

Mechanical testing revealed formulation‑dependent changes in stiffness and extensibility.

In hydrophilic PEO‑PLCL wound dressings, the applied drug loading increased Young’s modulus (from 1.99 to 3.18 MPa) and reduced both elongation at maximum load and at rupture (1.68 to 0.79 mm and 5.22 to 1.67 mm, respectively), indicating a stiffer yet more brittle network.

In contrast, pristine CA‑PLCL samples exhibited the highest load‑bearing capacity and extensibility (Fmax = 0.60 N, dL at Fmax = 3.46 mm), whereas loading decreased Fmax to 0.24 N and halved the elongation at maximum load without substantially altering modulus (~1.15 MPa).

Together, these data show that drug incorporation predominantly compromises failure strain—especially in CA‑PLCL—while preserving or even increasing initial stiffness, which is critical for handling yet may limit conformability on moving tissue surfaces. The force (F_break_) and length at break (dL) was unmeasurably low for CA-PLCL loaded samples.

Across all pristine vs loaded comparisons, dL at break decreases markedly with loading (from 5.22 to 1.67 mm in PEO‑PLCL), supporting the conclusion that drug incorporation promotes earlier failure and reduced deformability in the case of the nanofiber-based wound dressings of polymer compositions.

For PEO‑PLCL, both pristine and loaded samples show relatively high extensibility but limited toughness: dL at break is high for pristine (5.22 mm) and still substantial for loaded (1.67 mm), confirming that they can be elongated visibly before failure. By CA‑PLCL samples however, ductile separation could be observed, the separation was resembling a sliding effect at slow external forces applied. Figure …shows typical force-dtrain diagrams of the tested samples.


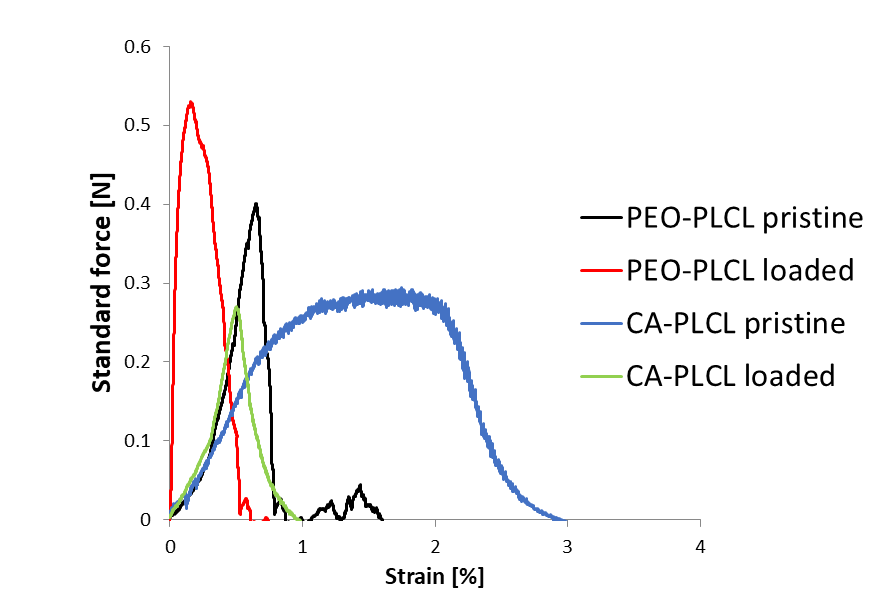


Figure Characteristic force-strain diagram of the tested samples.

Mechanically, this matches a more ductile, fibrillating failure mode: under slow pulling, fibres and bundles reorient and slide, the mat necks and opens, and the structure gradually loses integrity rather than fracturing sharply. Drug loading reduces Fmax (0.24 N) and dL at break (1.74 mm) without changing modulus substantially (~1.14 MPa), with CA‑PLCL-based dressings the network architecture is weakened, less force lead to a displacement.
