# Supplementary figures and images for: Hybrid Nanofibers for Multimodal Accelerated Wound Healing
Source: Adv Healthc Mater. 2026 Jan 28;15(15):e04029. doi: 10.1002/adhm.202504029 (PMC13088746; doi:10.1002/adhm.202504029)

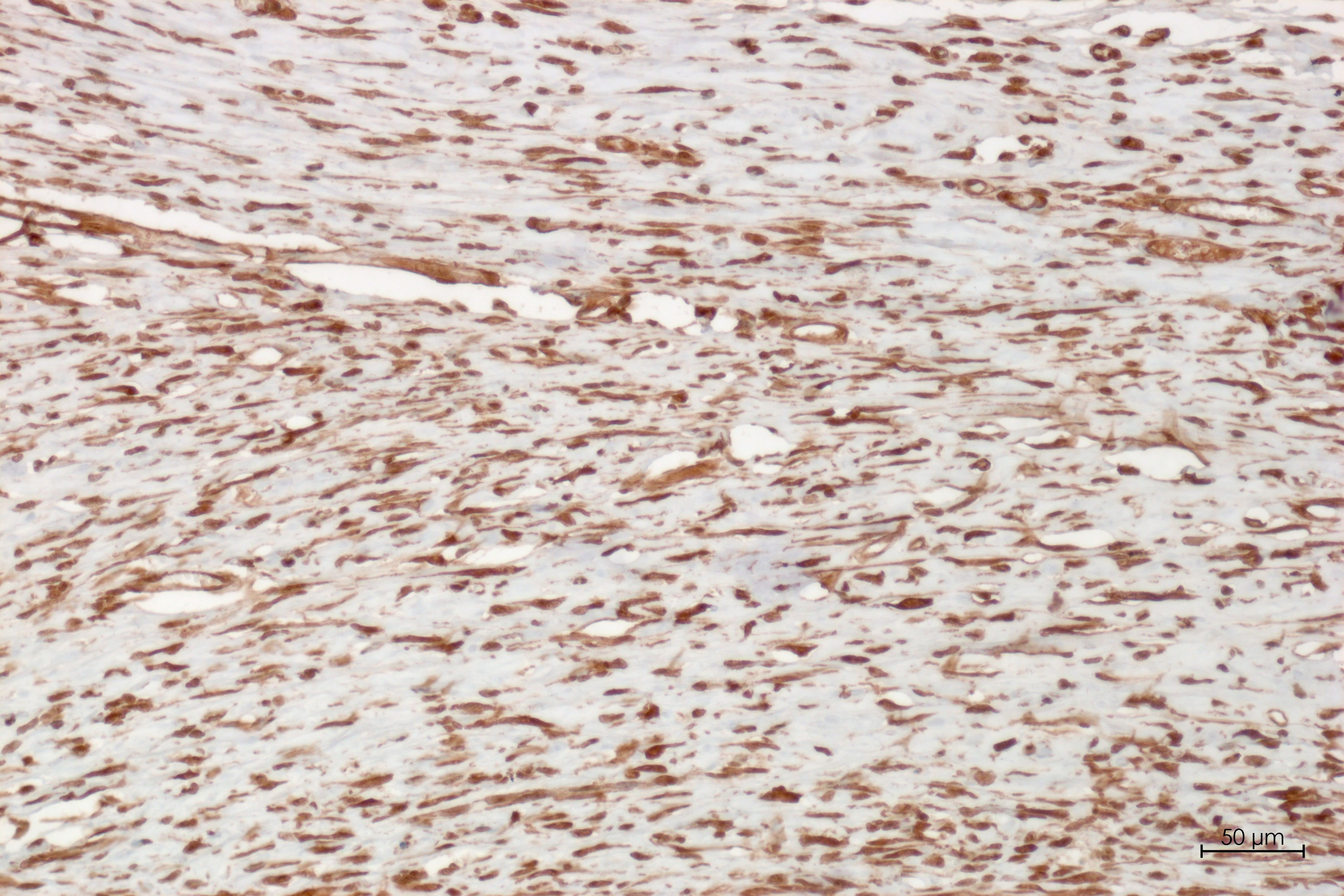

Supplement: Supplementary file 2 — Supporting file 2: adhm70839‐sup‐0002‐Complete Data.zip [file ADHM-15-0-s001.zip › Complete Data/Histology/alpha SMA/aSMA-CA-PLCL loaded.jpg]

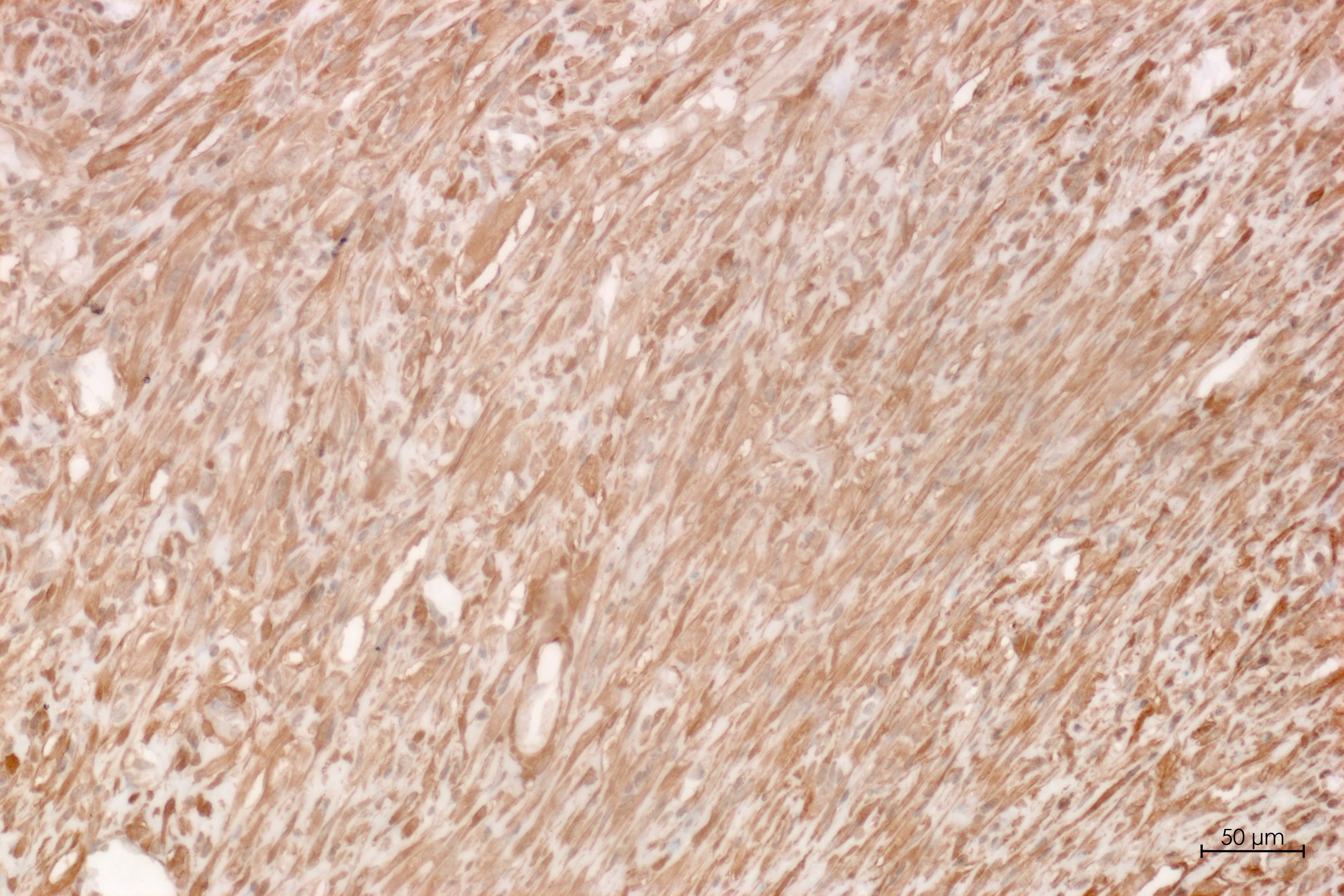

Supplement: Supplementary file 2 — Supporting file 2: adhm70839‐sup‐0002‐Complete Data.zip [file ADHM-15-0-s001.zip › Complete Data/Histology/alpha SMA/aSMA-control.jpg]

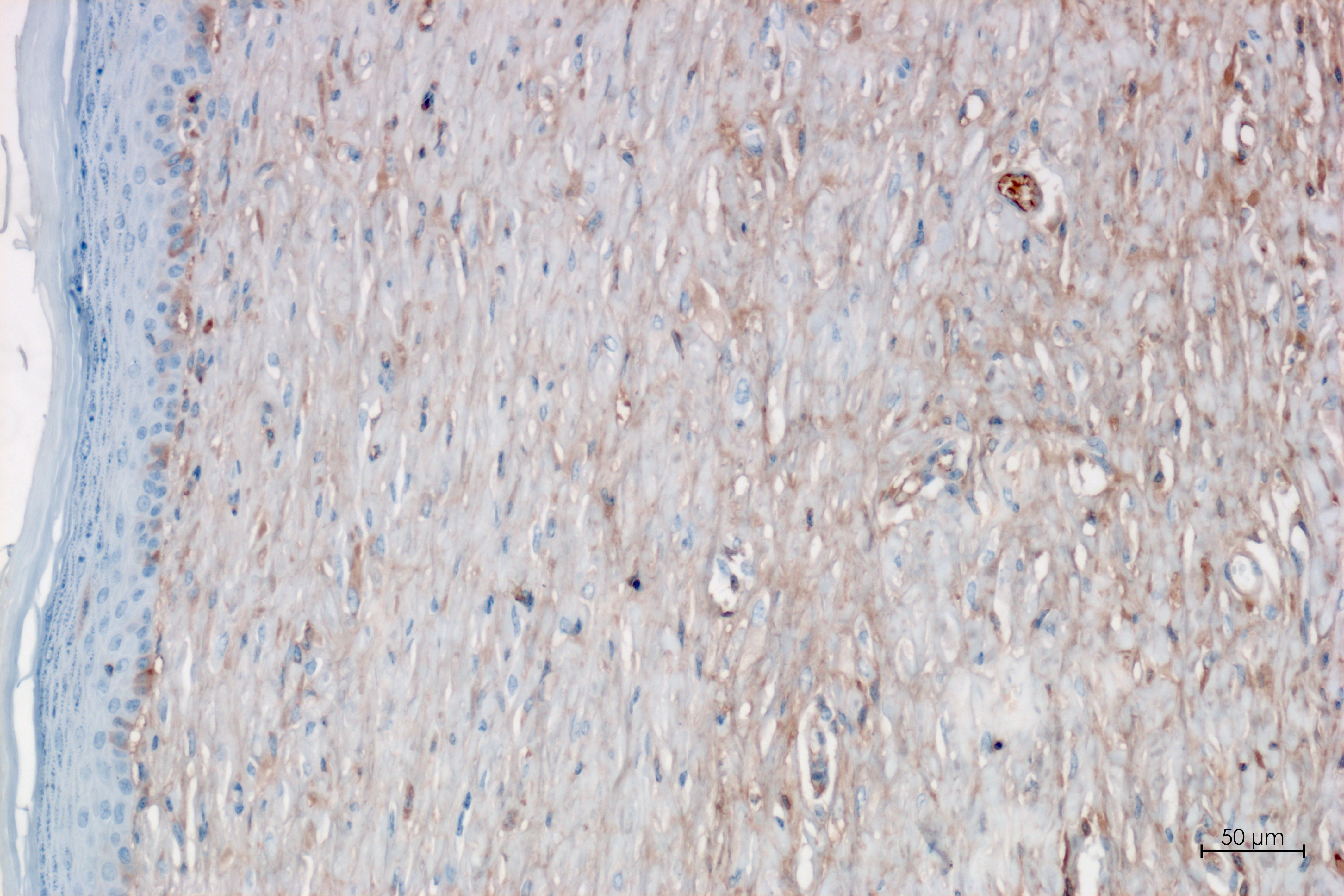

Supplement: Supplementary file 2 — Supporting file 2: adhm70839‐sup‐0002‐Complete Data.zip [file ADHM-15-0-s001.zip › Complete Data/Histology/CD3/CD3-CA-PLCL loaded.jpg]

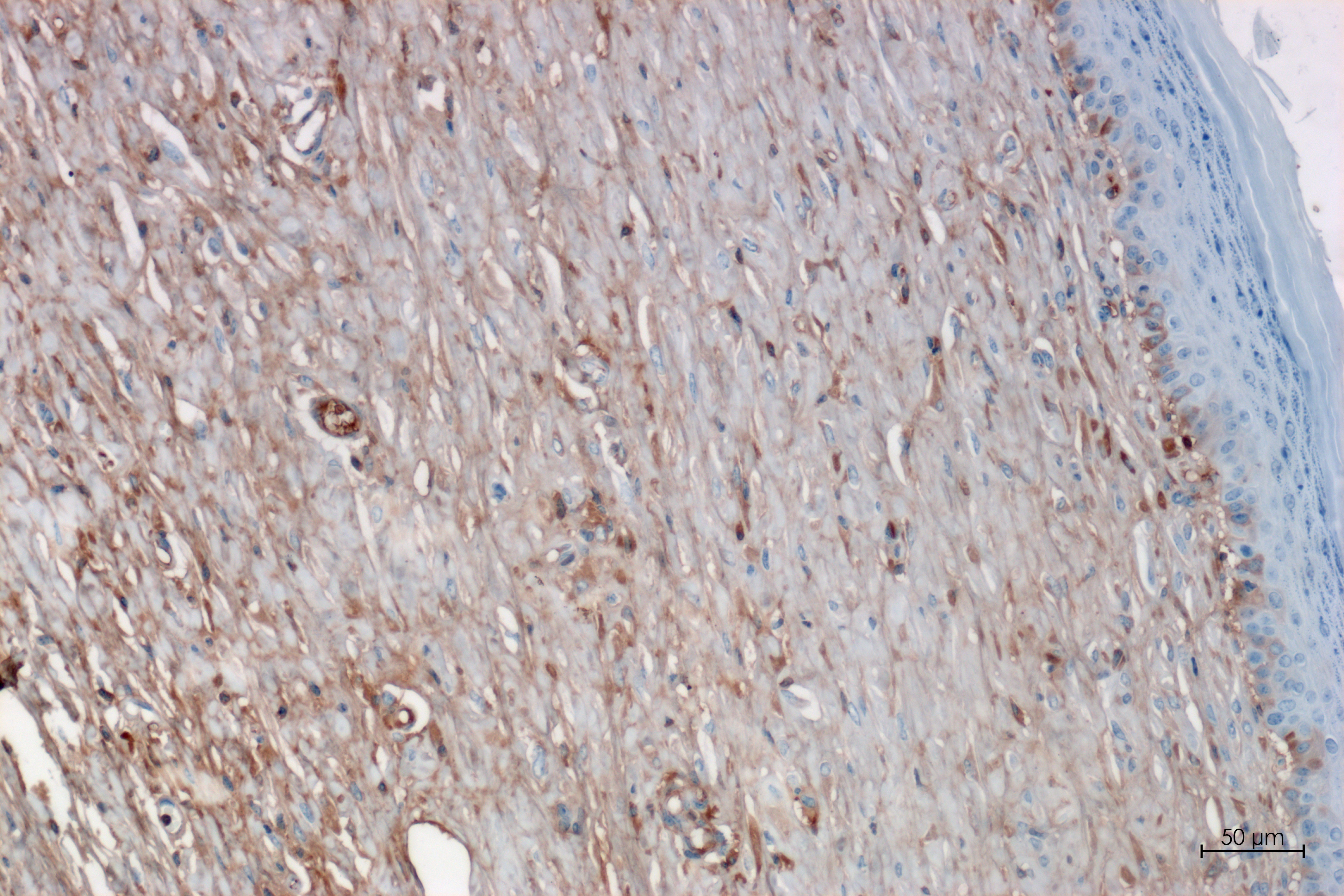

Supplement: Supplementary file 2 — Supporting file 2: adhm70839‐sup‐0002‐Complete Data.zip [file ADHM-15-0-s001.zip › Complete Data/Histology/CD3/CD3-CA-PLCL pristine.jpg]

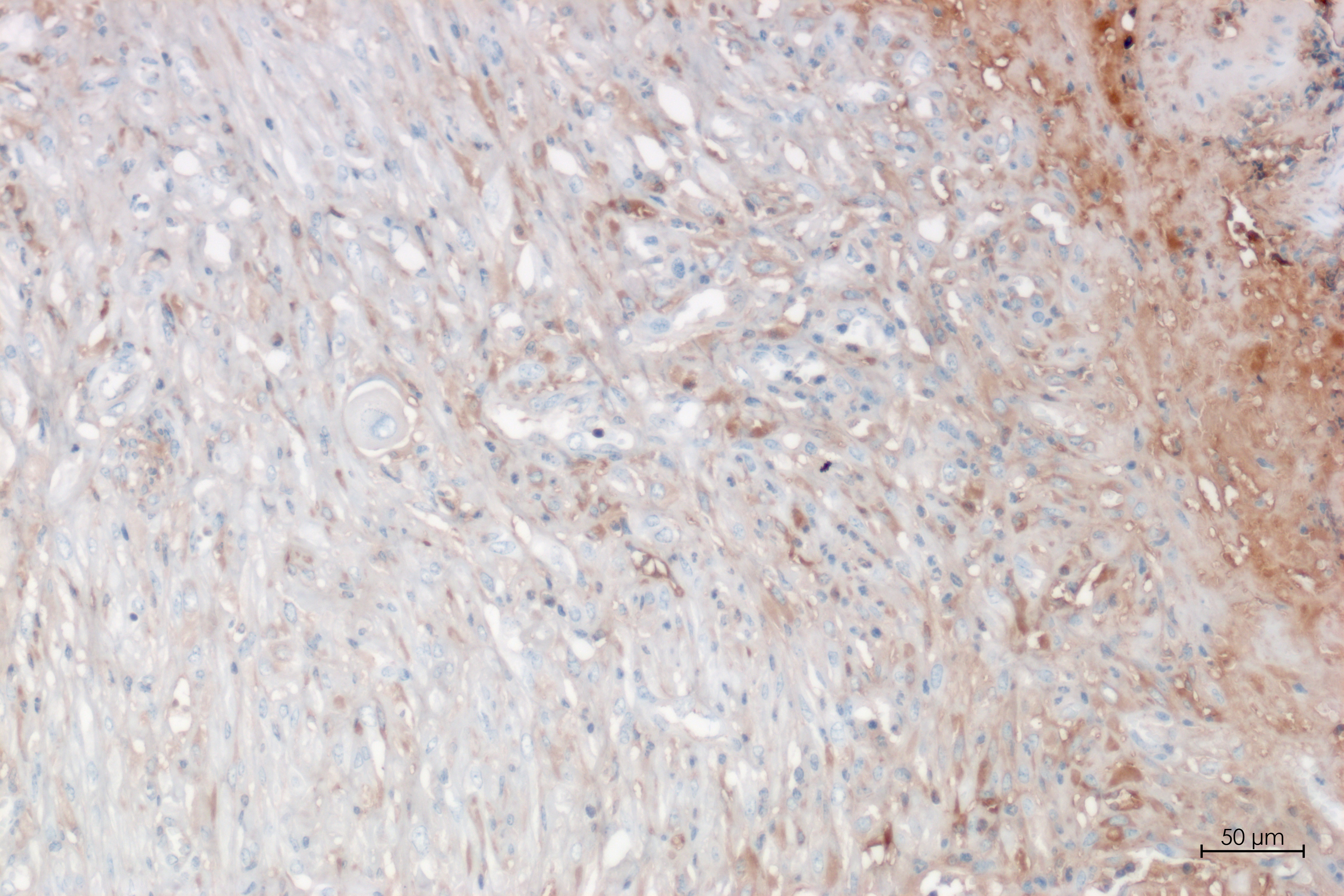

Supplement: Supplementary file 2 — Supporting file 2: adhm70839‐sup‐0002‐Complete Data.zip [file ADHM-15-0-s001.zip › Complete Data/Histology/CD3/CD3-control.jpg]

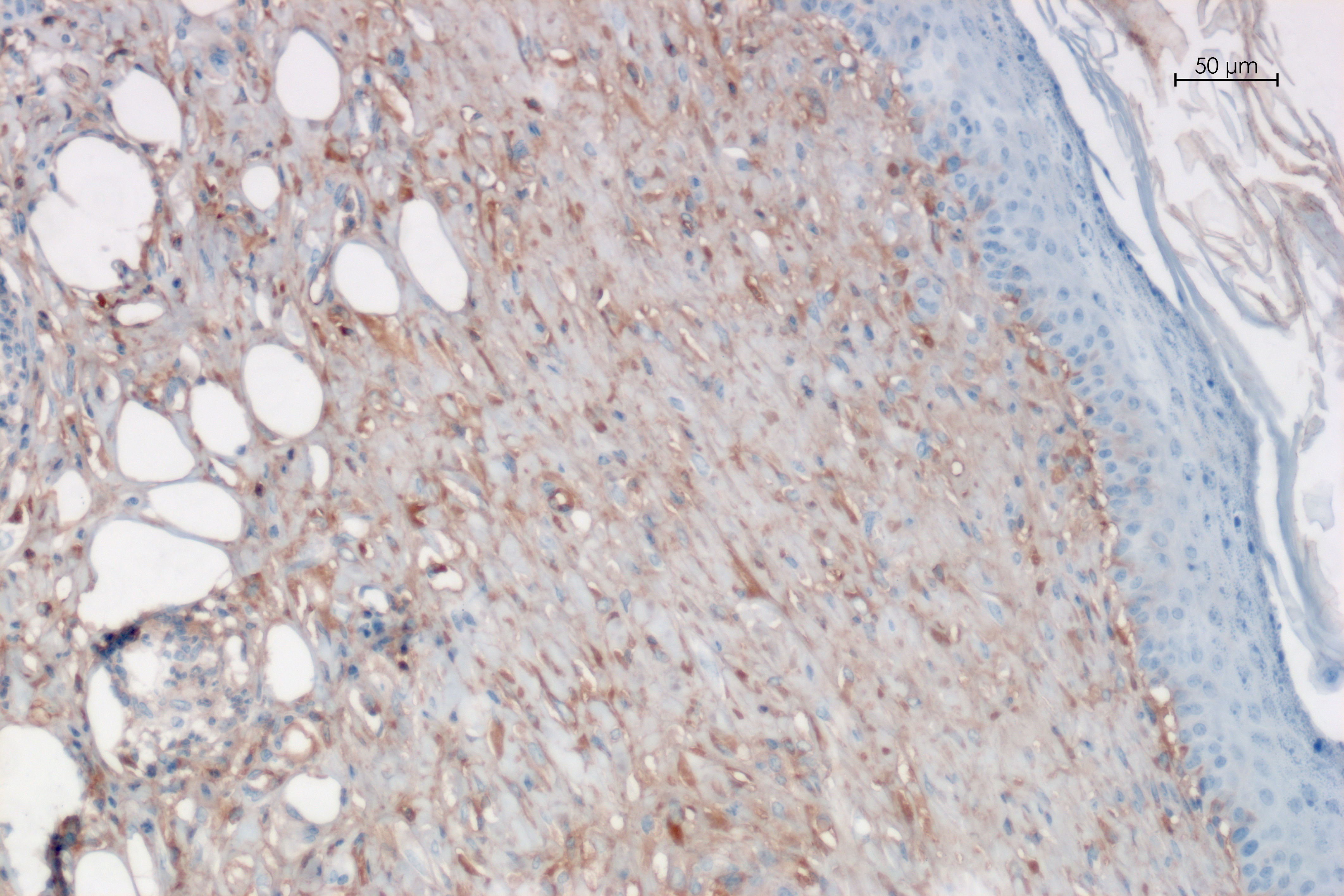

Supplement: Supplementary file 2 — Supporting file 2: adhm70839‐sup‐0002‐Complete Data.zip [file ADHM-15-0-s001.zip › Complete Data/Histology/CD3/CD3-PEO-PLCL loaded.jpg]

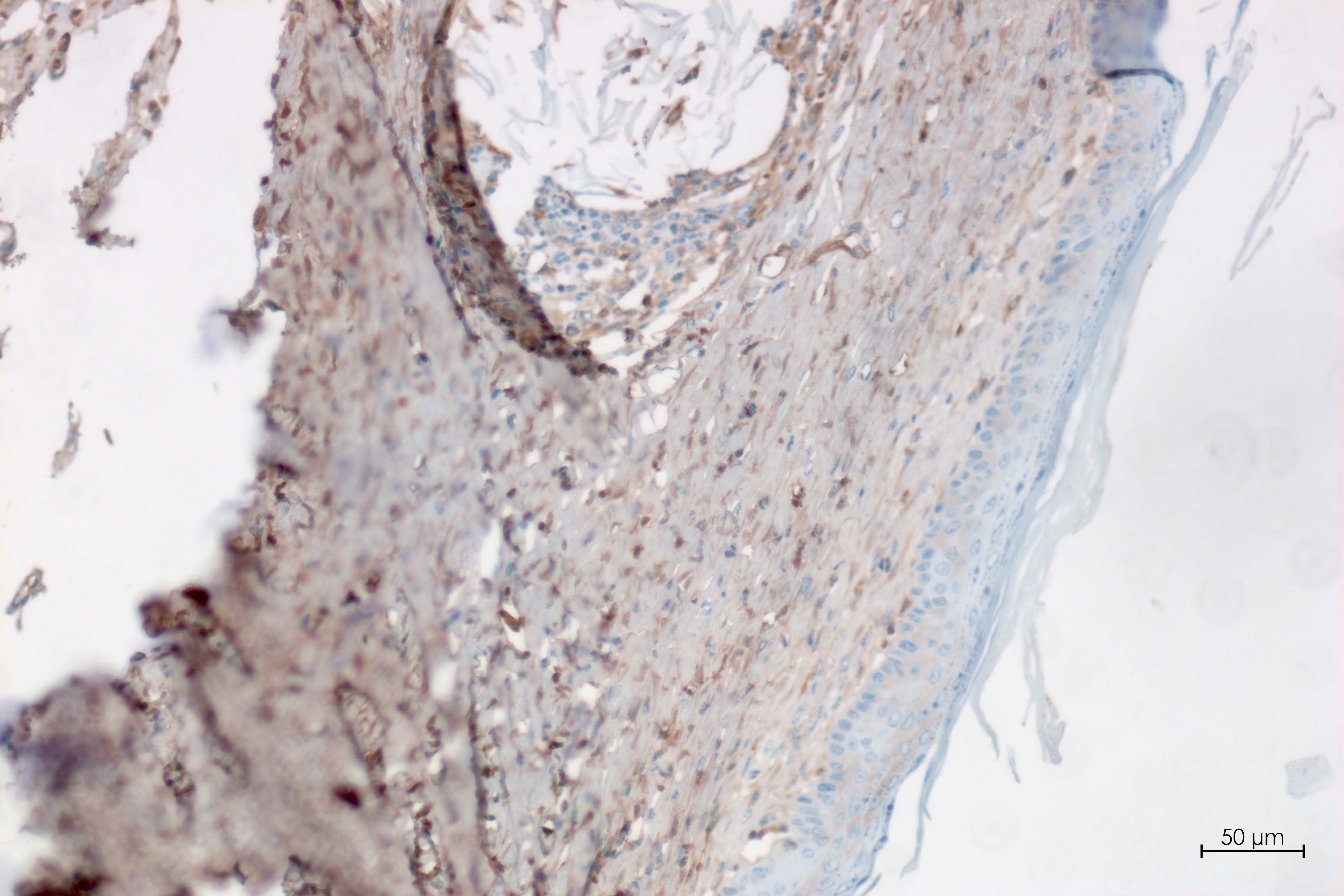

Supplement: Supplementary file 2 — Supporting file 2: adhm70839‐sup‐0002‐Complete Data.zip [file ADHM-15-0-s001.zip › Complete Data/Histology/CD3/CD3-PEO-PLCL pristine.jpg]

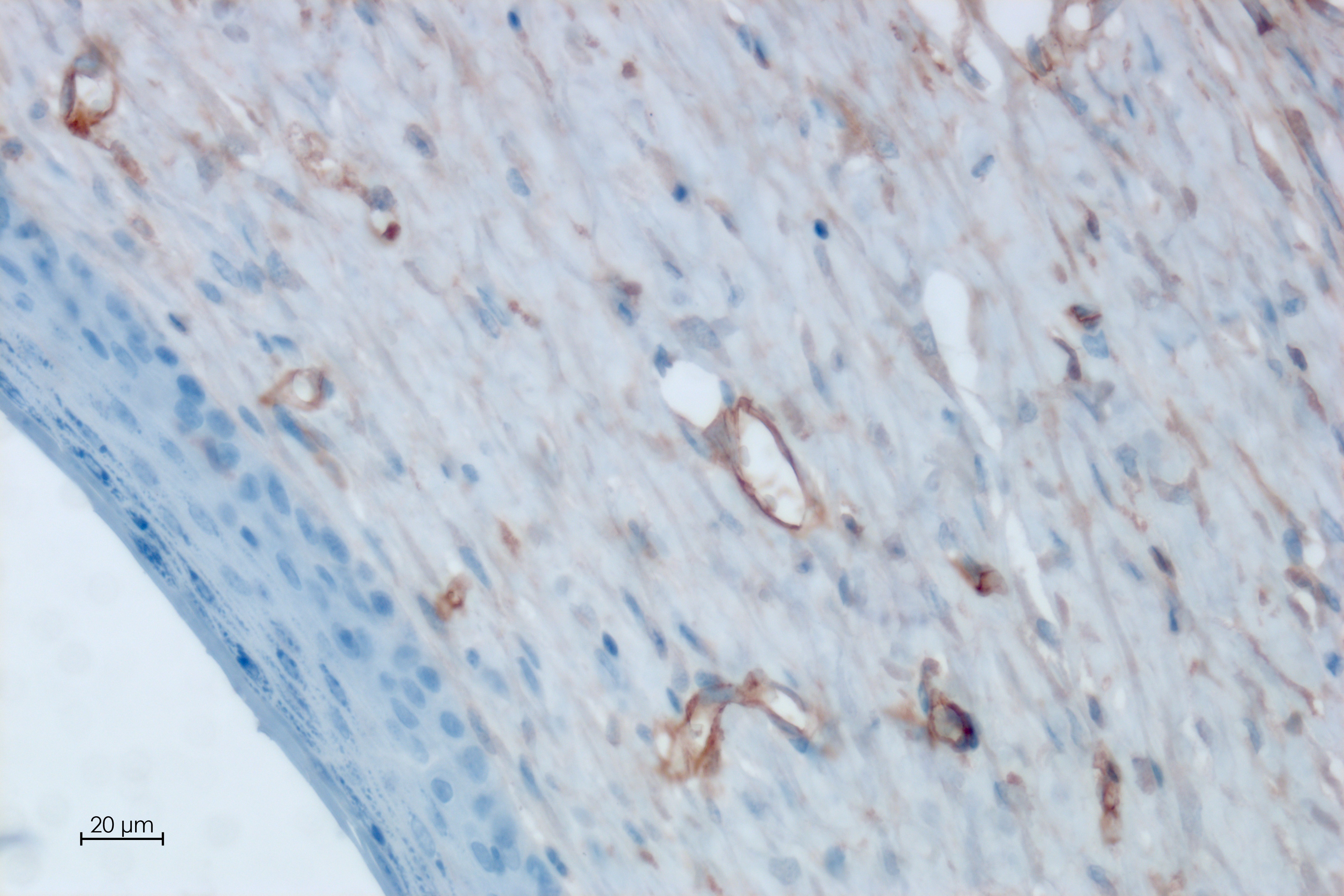

Supplement: Supplementary file 2 — Supporting file 2: adhm70839‐sup‐0002‐Complete Data.zip [file ADHM-15-0-s001.zip › Complete Data/Histology/CD34/CD34-CA-PLCL loaded.jpg]

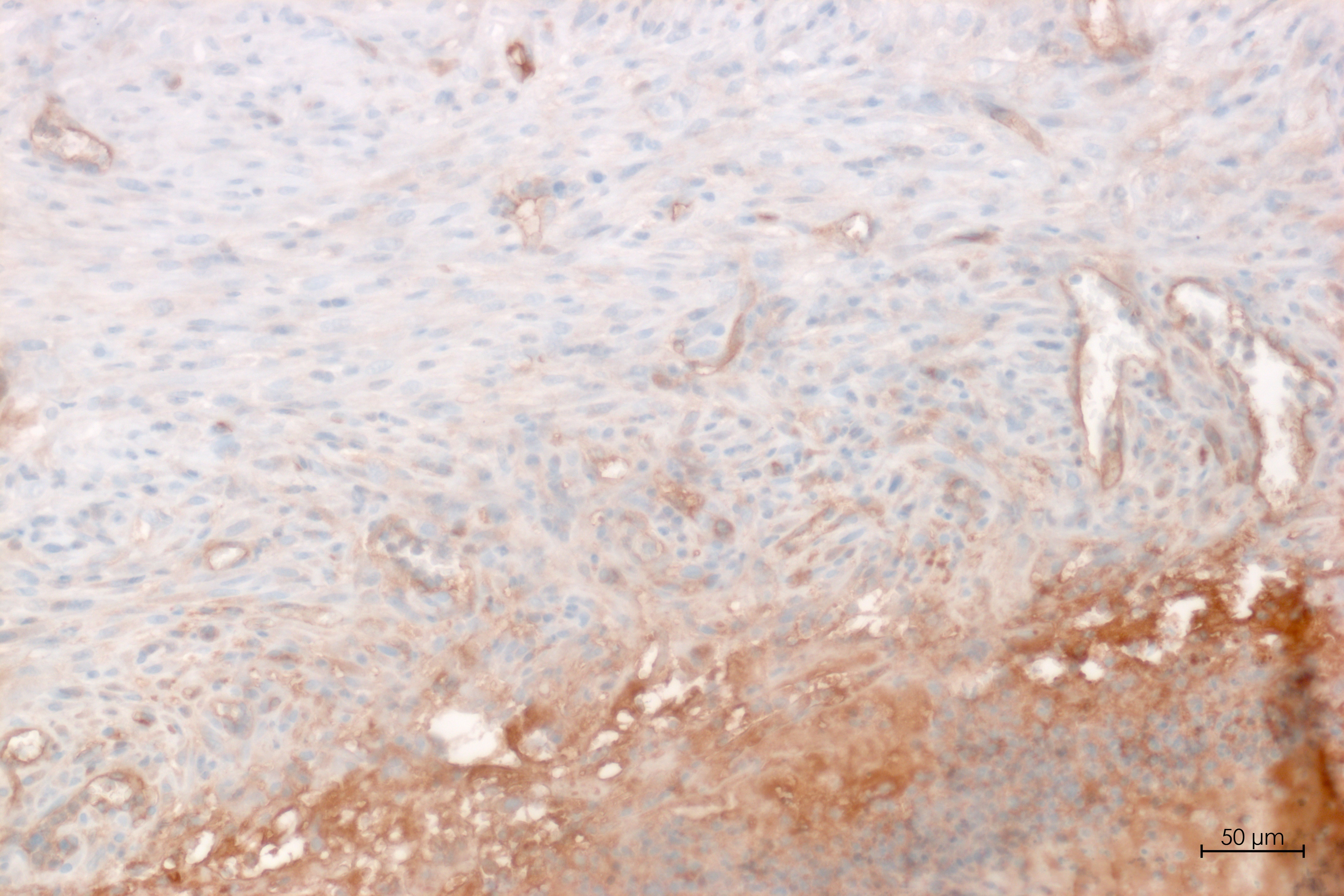

Supplement: Supplementary file 2 — Supporting file 2: adhm70839‐sup‐0002‐Complete Data.zip [file ADHM-15-0-s001.zip › Complete Data/Histology/CD34/CD34-control.jpg]

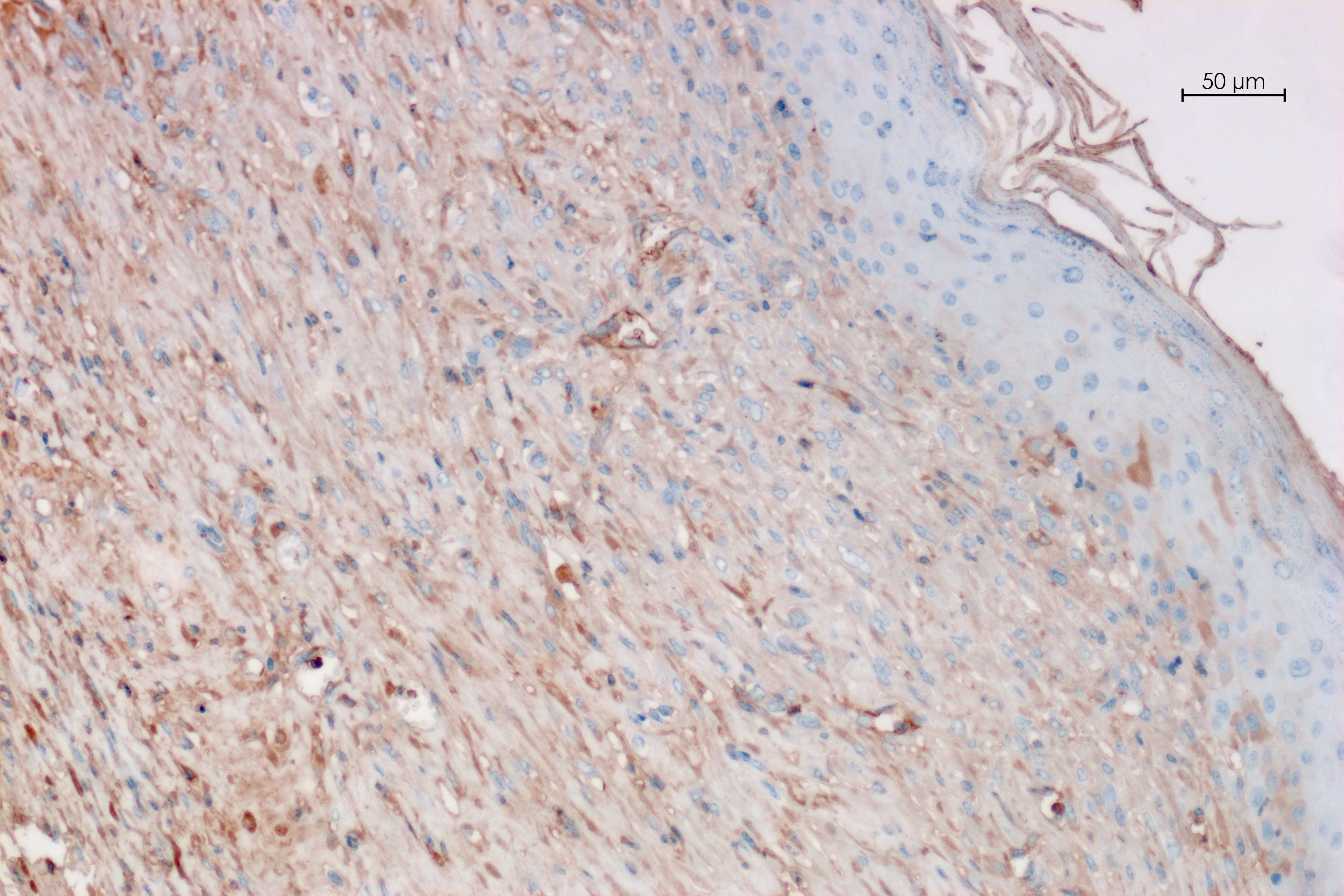

Supplement: Supplementary file 2 — Supporting file 2: adhm70839‐sup‐0002‐Complete Data.zip [file ADHM-15-0-s001.zip › Complete Data/Histology/CD45/CD45-CA-PLCL loaded.jpg]

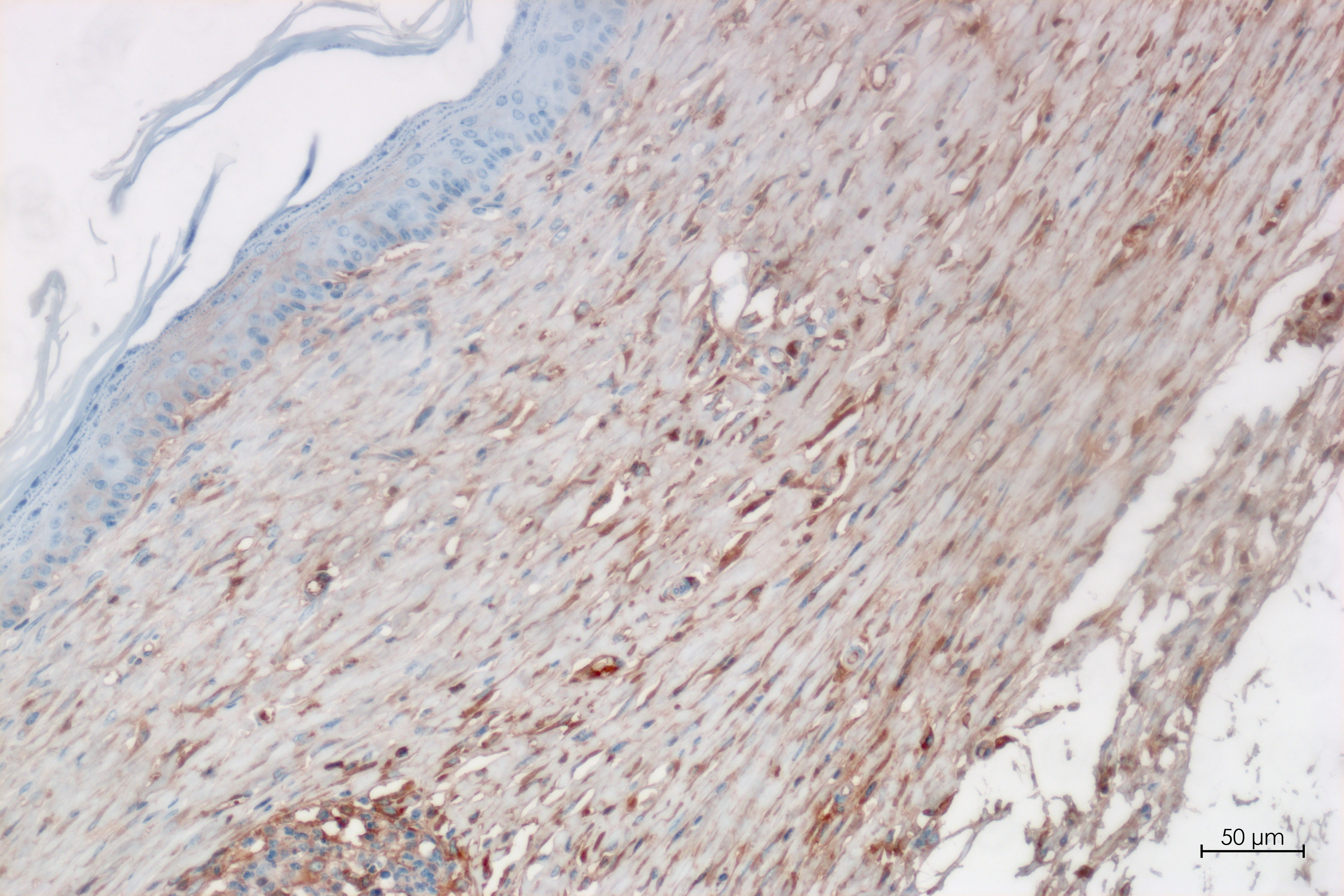

Supplement: Supplementary file 2 — Supporting file 2: adhm70839‐sup‐0002‐Complete Data.zip [file ADHM-15-0-s001.zip › Complete Data/Histology/CD45/CD45-CA-PLCL pristine.jpg]

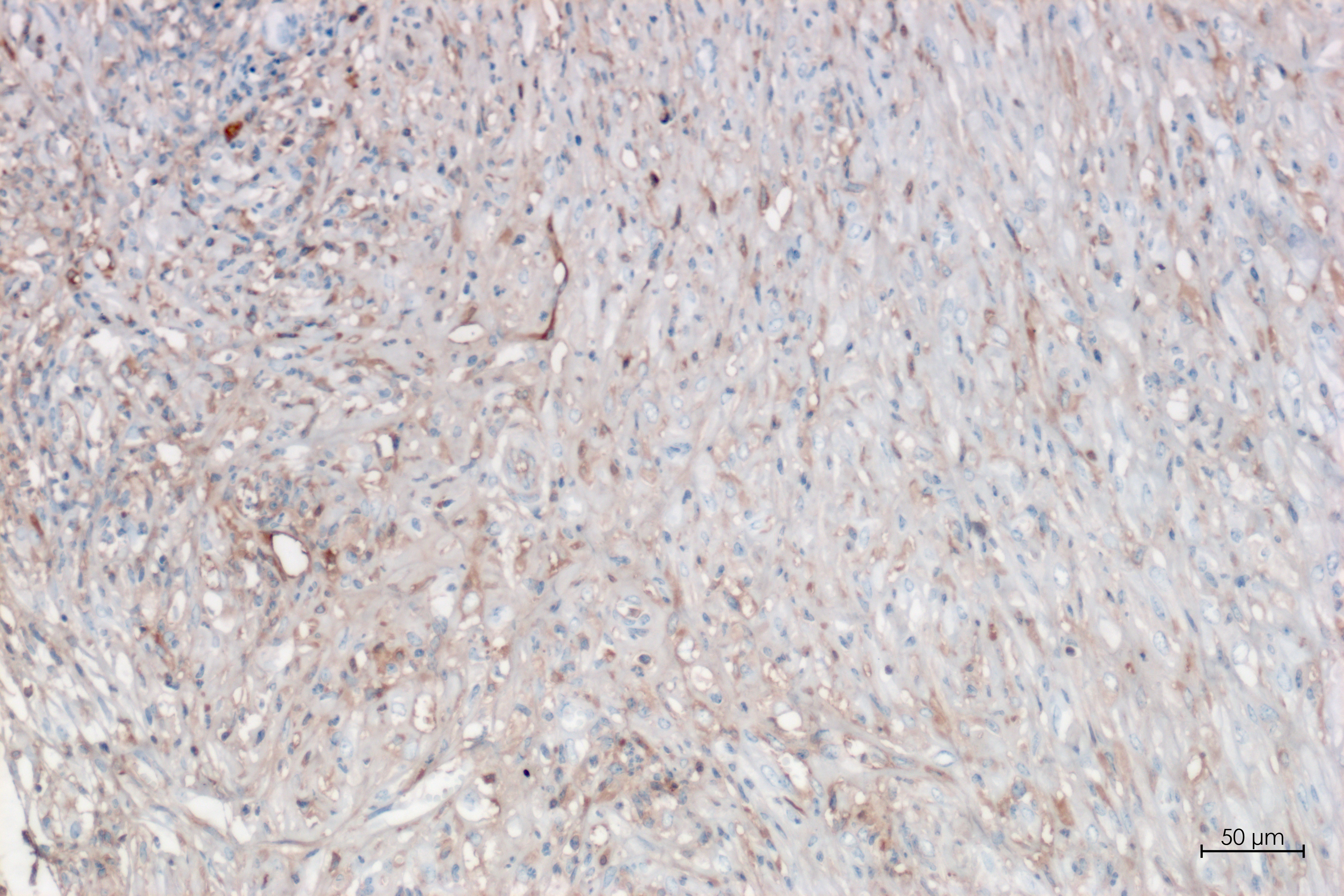

Supplement: Supplementary file 2 — Supporting file 2: adhm70839‐sup‐0002‐Complete Data.zip [file ADHM-15-0-s001.zip › Complete Data/Histology/CD45/CD45-control.jpg]

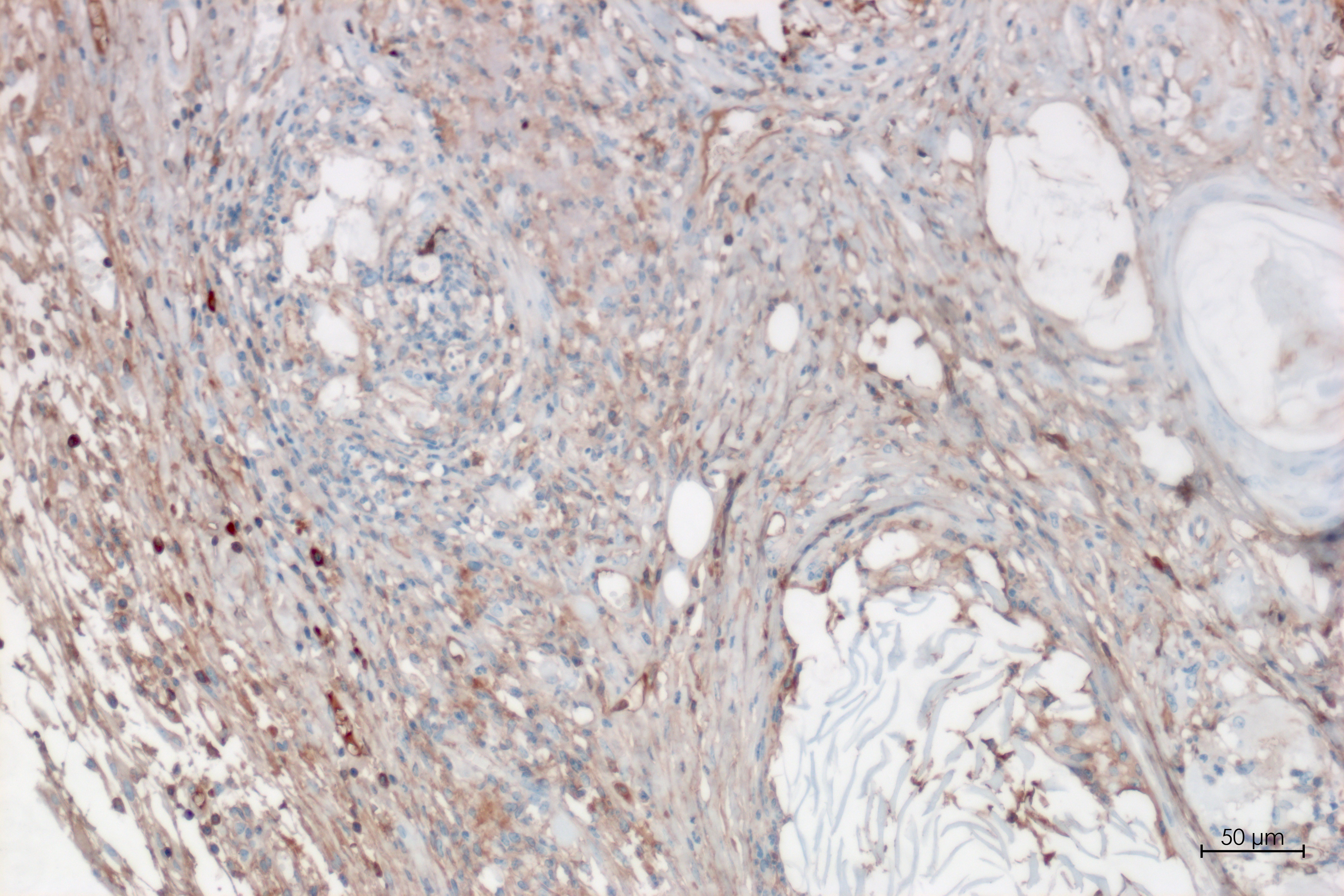

Supplement: Supplementary file 2 — Supporting file 2: adhm70839‐sup‐0002‐Complete Data.zip [file ADHM-15-0-s001.zip › Complete Data/Histology/CD45/CD45-PEO-PCL pristine.jpg]

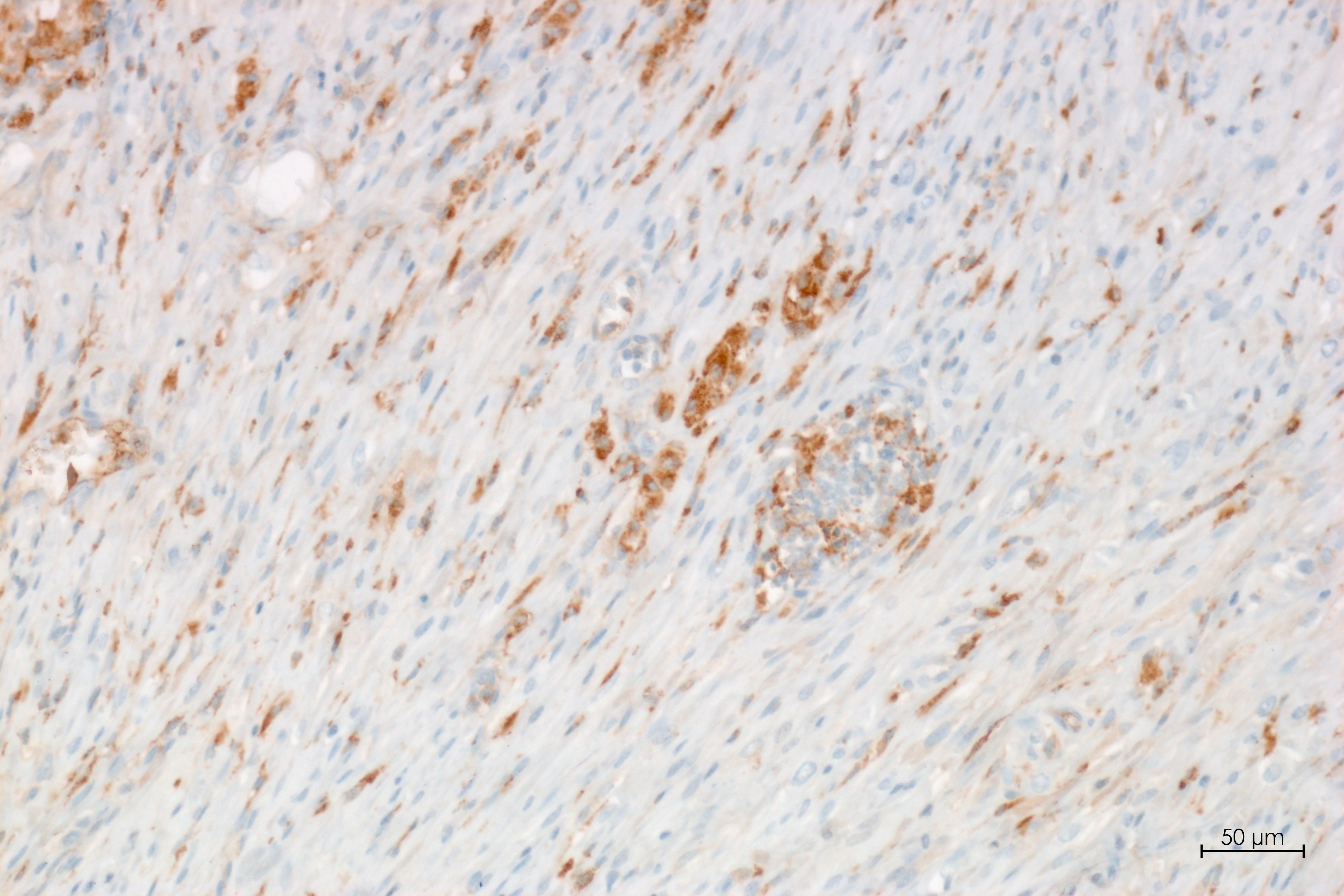

Supplement: Supplementary file 2 — Supporting file 2: adhm70839‐sup‐0002‐Complete Data.zip [file ADHM-15-0-s001.zip › Complete Data/Histology/CD68/CD68-CA-PLCL loaded.jpg]

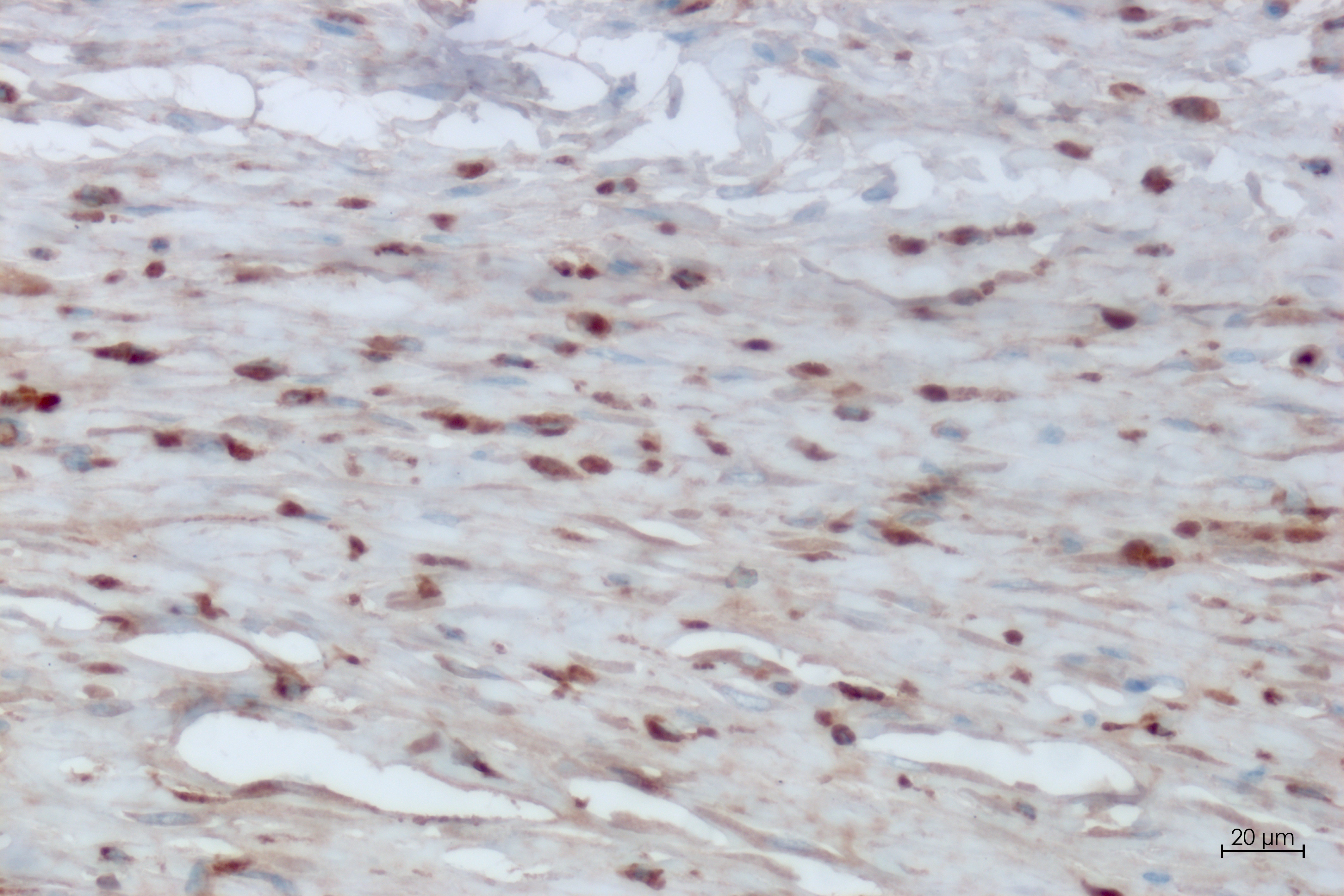

Supplement: Supplementary file 2 — Supporting file 2: adhm70839‐sup‐0002‐Complete Data.zip [file ADHM-15-0-s001.zip › Complete Data/Histology/CD68/CD68-CA-PLCL pristine.jpg]

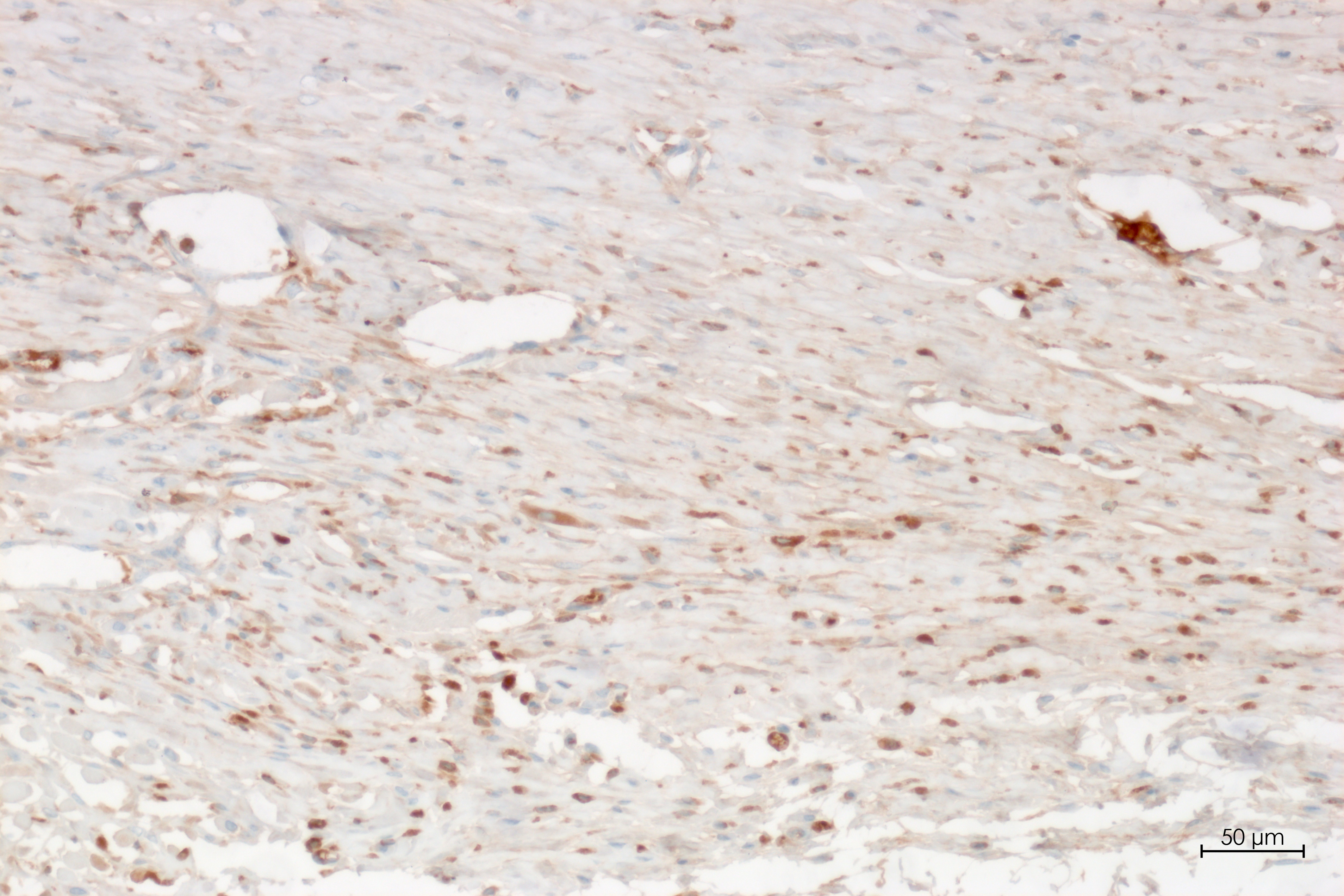

Supplement: Supplementary file 2 — Supporting file 2: adhm70839‐sup‐0002‐Complete Data.zip [file ADHM-15-0-s001.zip › Complete Data/Histology/CD68/CD68-control.jpg]

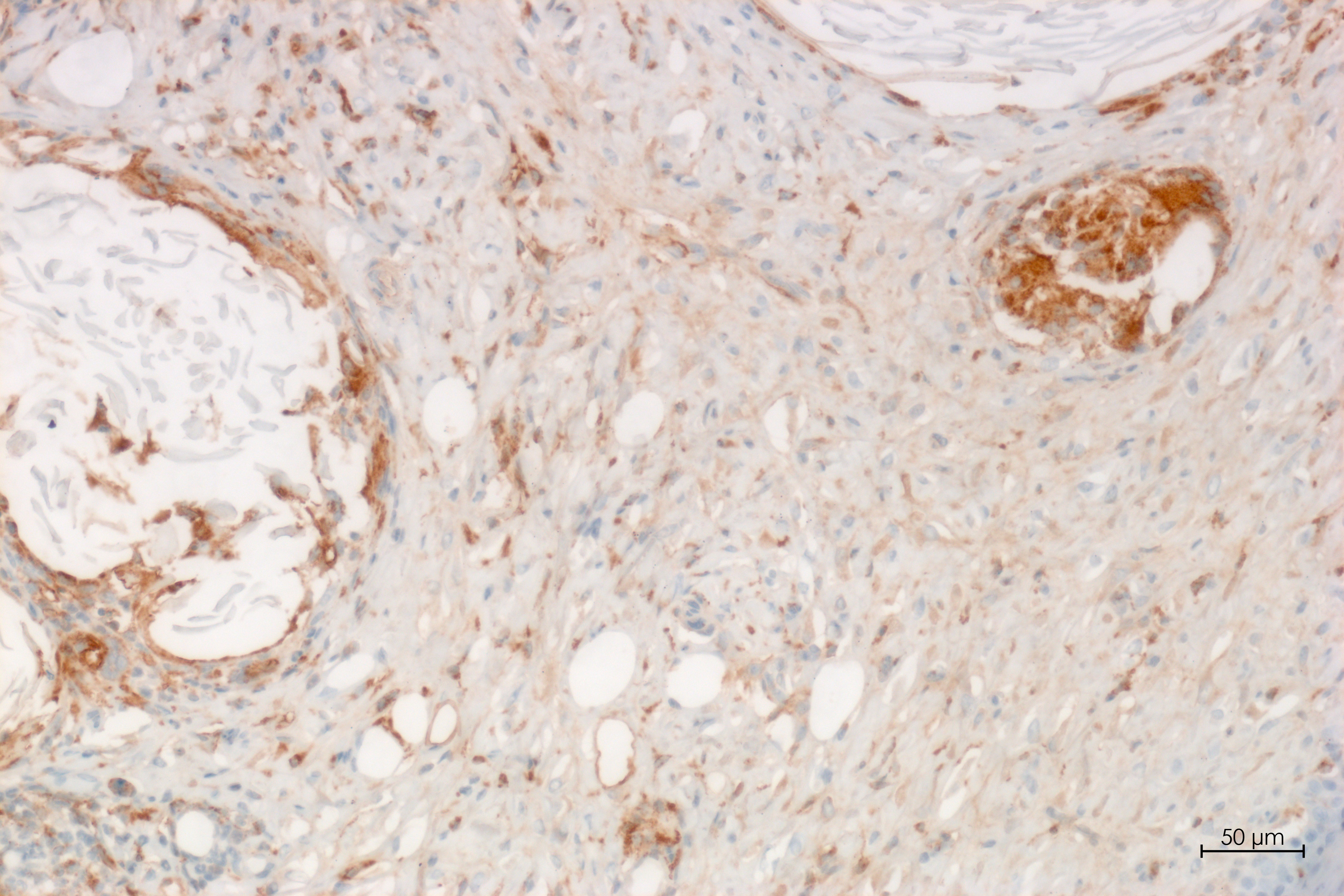

Supplement: Supplementary file 2 — Supporting file 2: adhm70839‐sup‐0002‐Complete Data.zip [file ADHM-15-0-s001.zip › Complete Data/Histology/CD68/CD68-PEO-PLCL pristine.jpg]

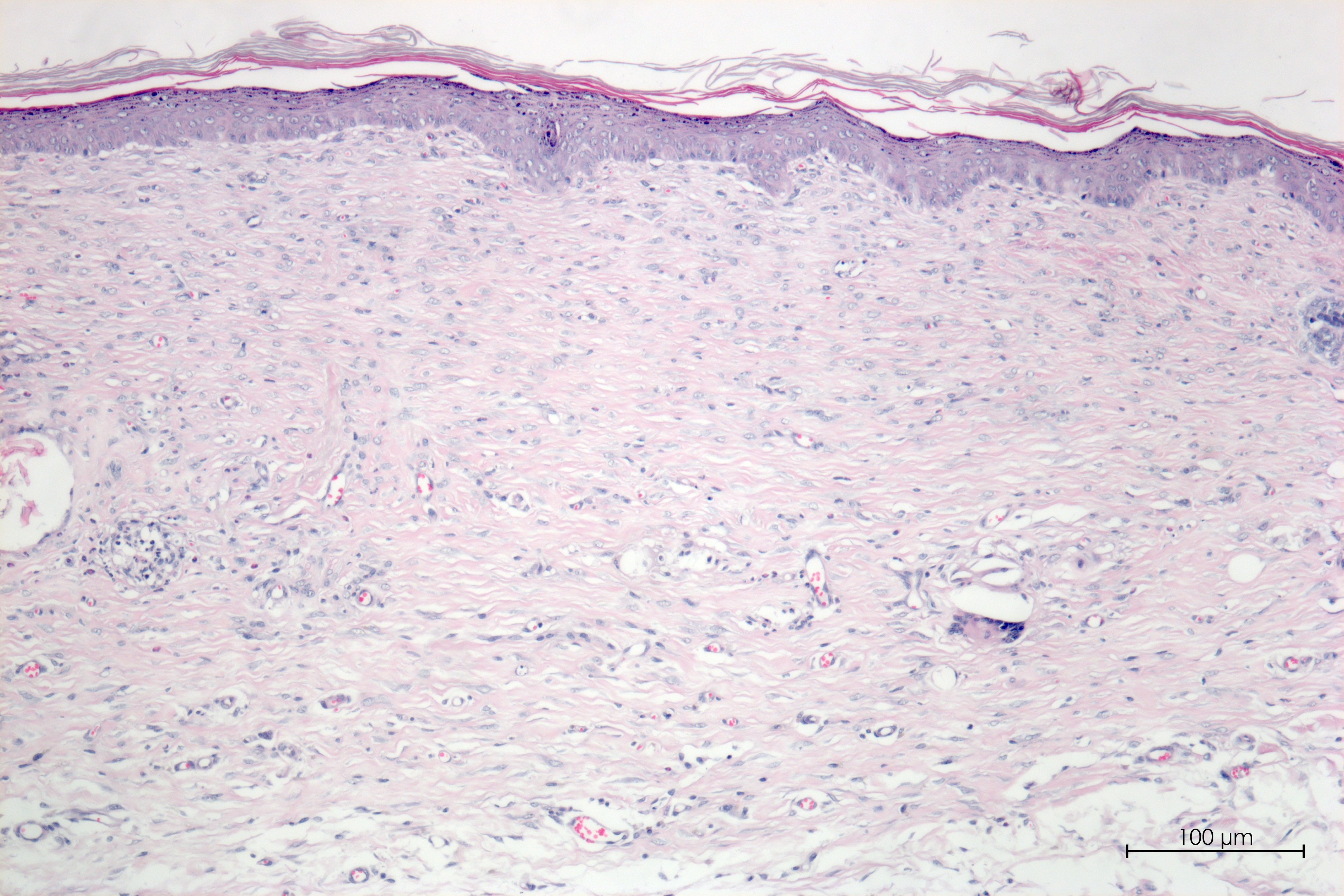

Supplement: Supplementary file 2 — Supporting file 2: adhm70839‐sup‐0002‐Complete Data.zip [file ADHM-15-0-s001.zip › Complete Data/Histology/H&E/CA-PLCL loaded.jpg]

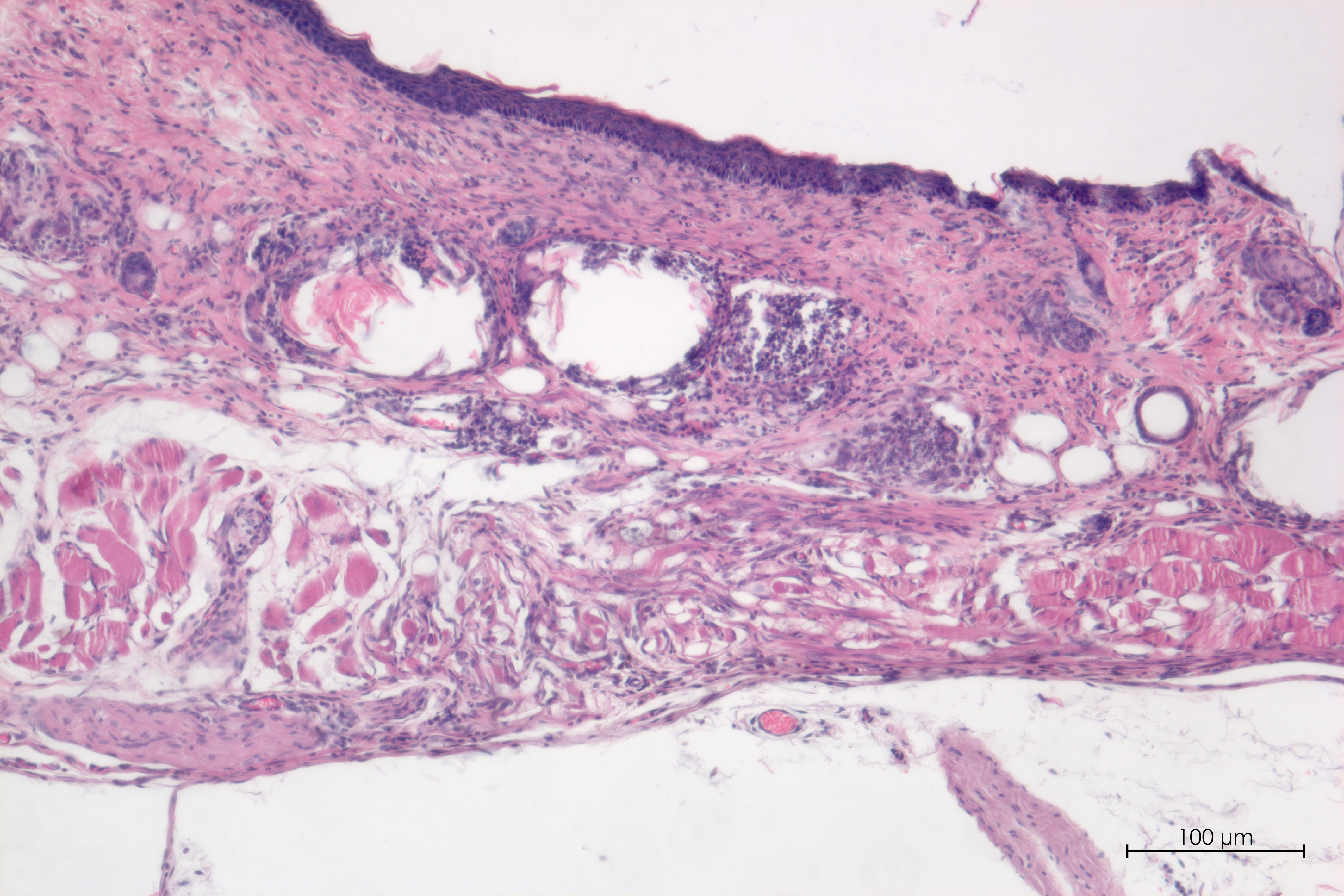

Supplement: Supplementary file 2 — Supporting file 2: adhm70839‐sup‐0002‐Complete Data.zip [file ADHM-15-0-s001.zip › Complete Data/Histology/H&E/CA-PLCL pristine.jpg]

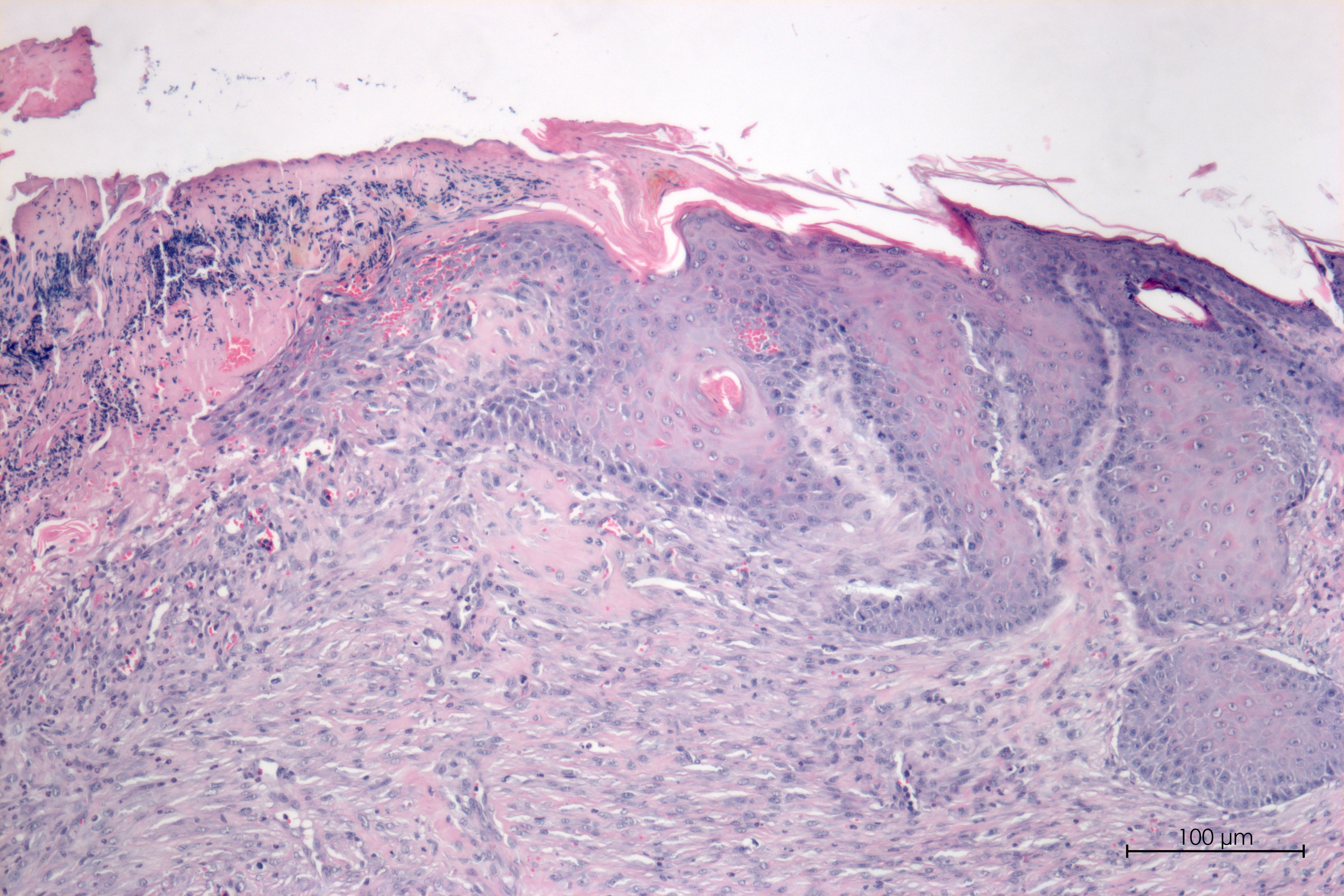

Supplement: Supplementary file 2 — Supporting file 2: adhm70839‐sup‐0002‐Complete Data.zip [file ADHM-15-0-s001.zip › Complete Data/Histology/H&E/Control.jpg]

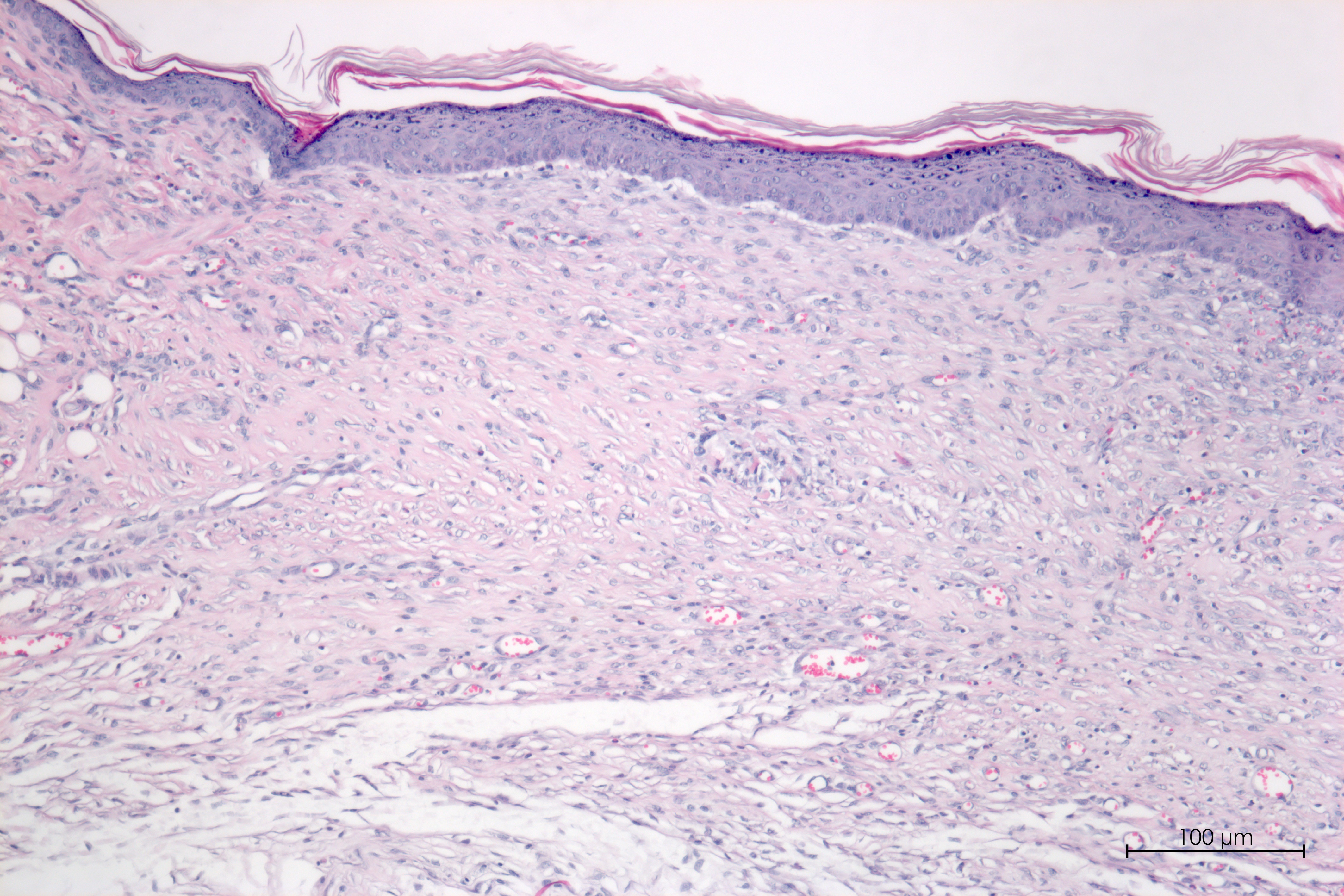

Supplement: Supplementary file 2 — Supporting file 2: adhm70839‐sup‐0002‐Complete Data.zip [file ADHM-15-0-s001.zip › Complete Data/Histology/H&E/PEO-PLCL loaded.jpg]

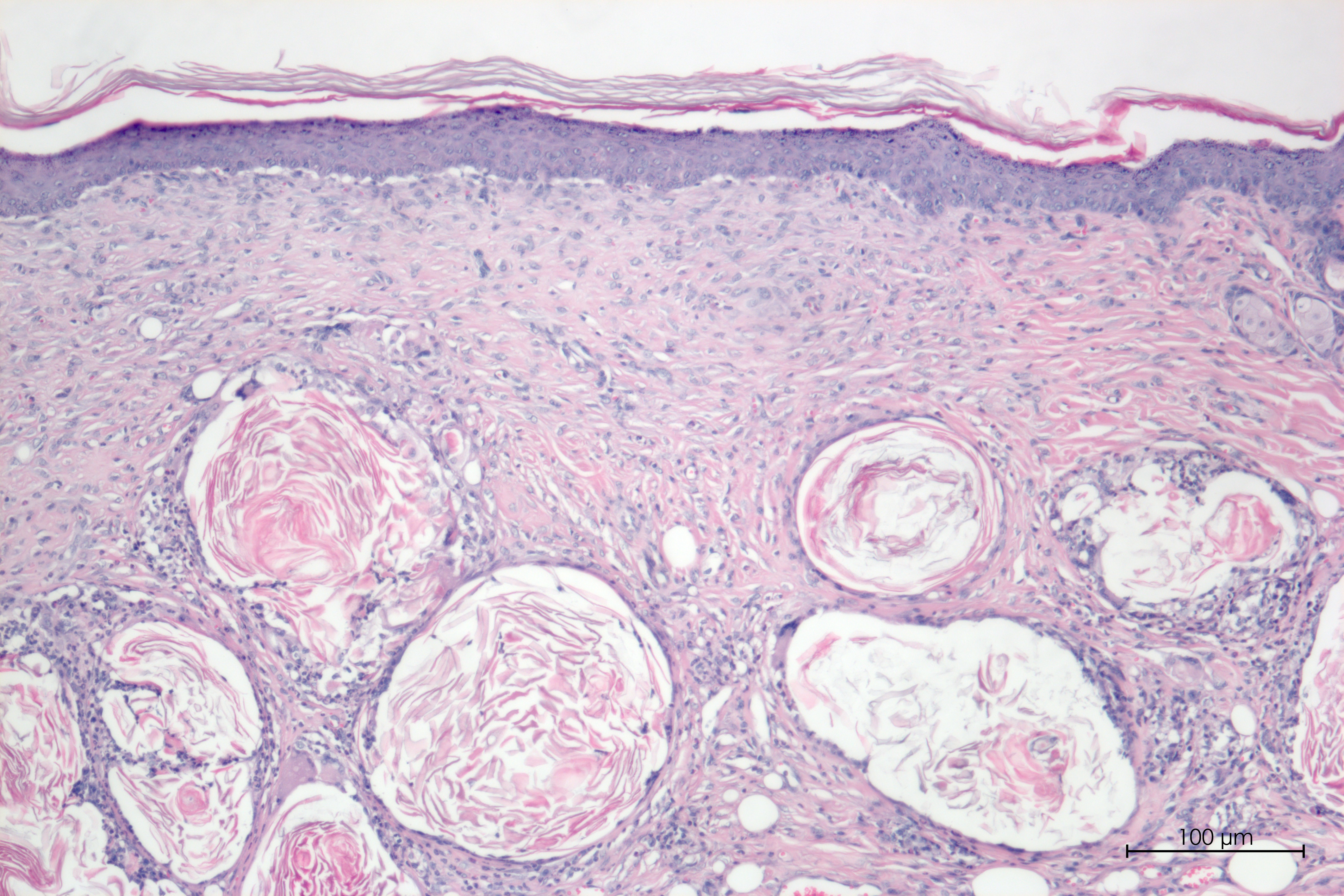

Supplement: Supplementary file 2 — Supporting file 2: adhm70839‐sup‐0002‐Complete Data.zip [file ADHM-15-0-s001.zip › Complete Data/Histology/H&E/PEO-PLCL pristine.jpg]

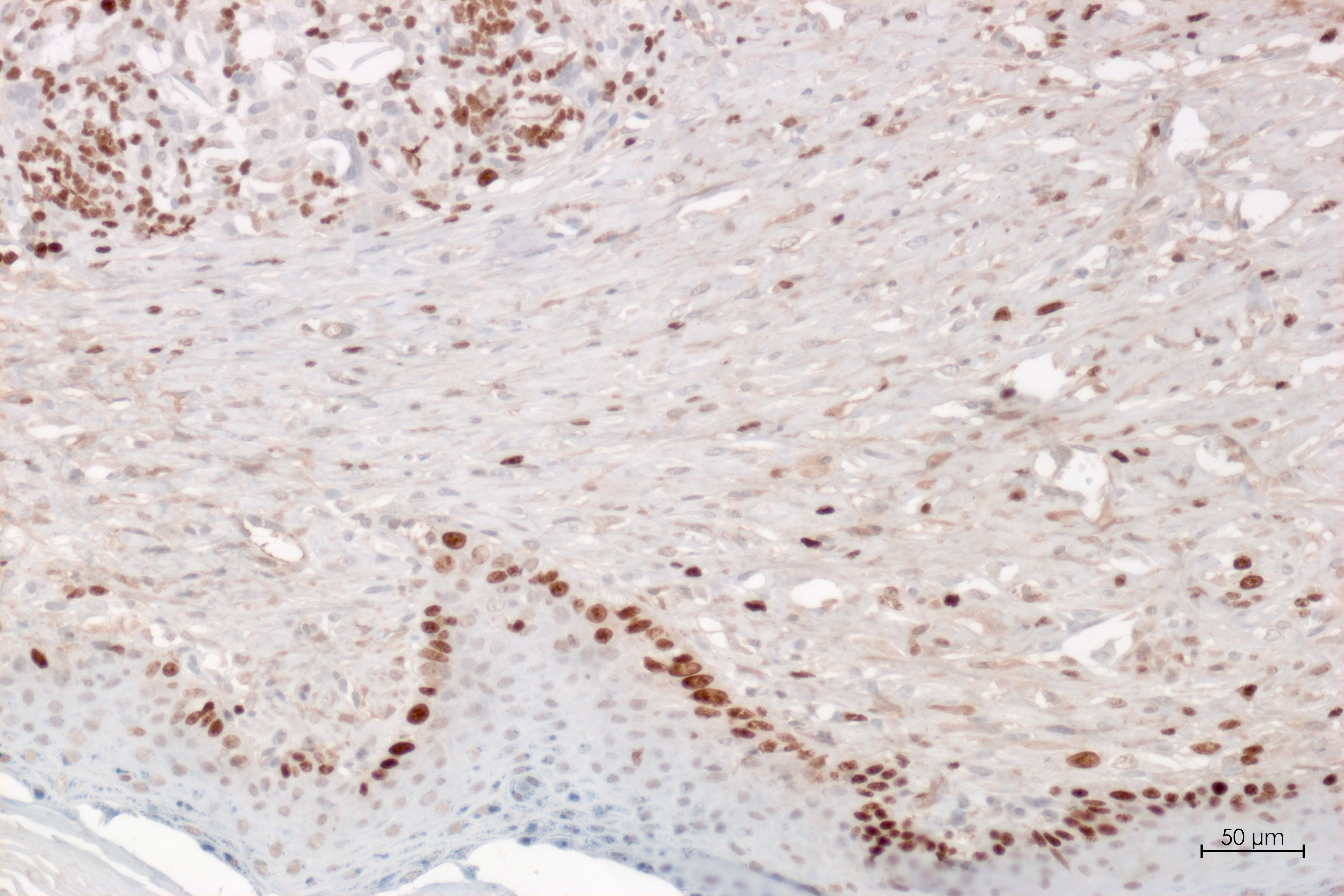

Supplement: Supplementary file 2 — Supporting file 2: adhm70839‐sup‐0002‐Complete Data.zip [file ADHM-15-0-s001.zip › Complete Data/Histology/Ki67/Ki-67-PEO-PLCL loaded.jpg]

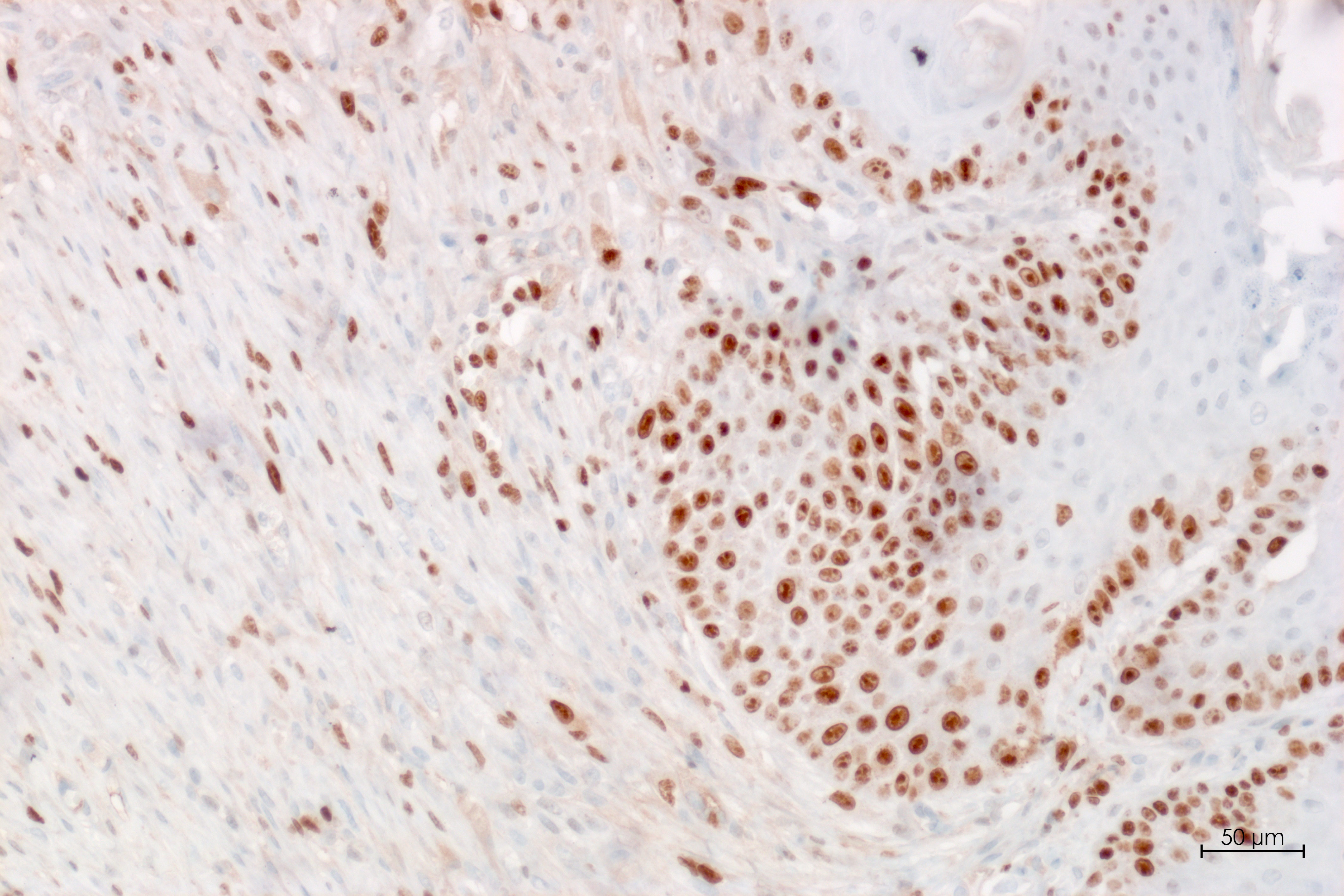

Supplement: Supplementary file 2 — Supporting file 2: adhm70839‐sup‐0002‐Complete Data.zip [file ADHM-15-0-s001.zip › Complete Data/Histology/Ki67/Ki67-control.jpg]

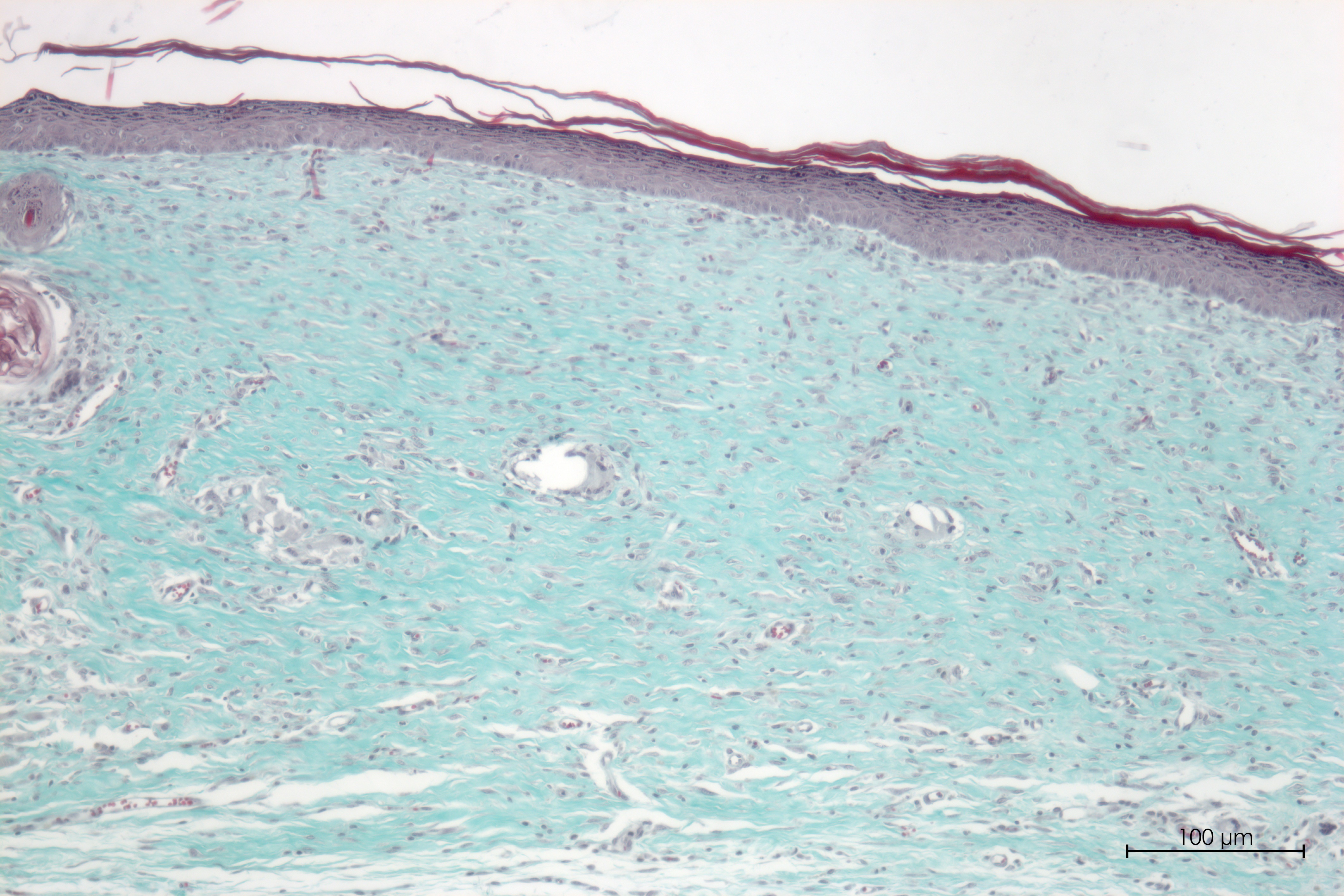

Supplement: Supplementary file 2 — Supporting file 2: adhm70839‐sup‐0002‐Complete Data.zip [file ADHM-15-0-s001.zip › Complete Data/Histology/Trichrome/CA-PLCL loaded Trichr.jpg]

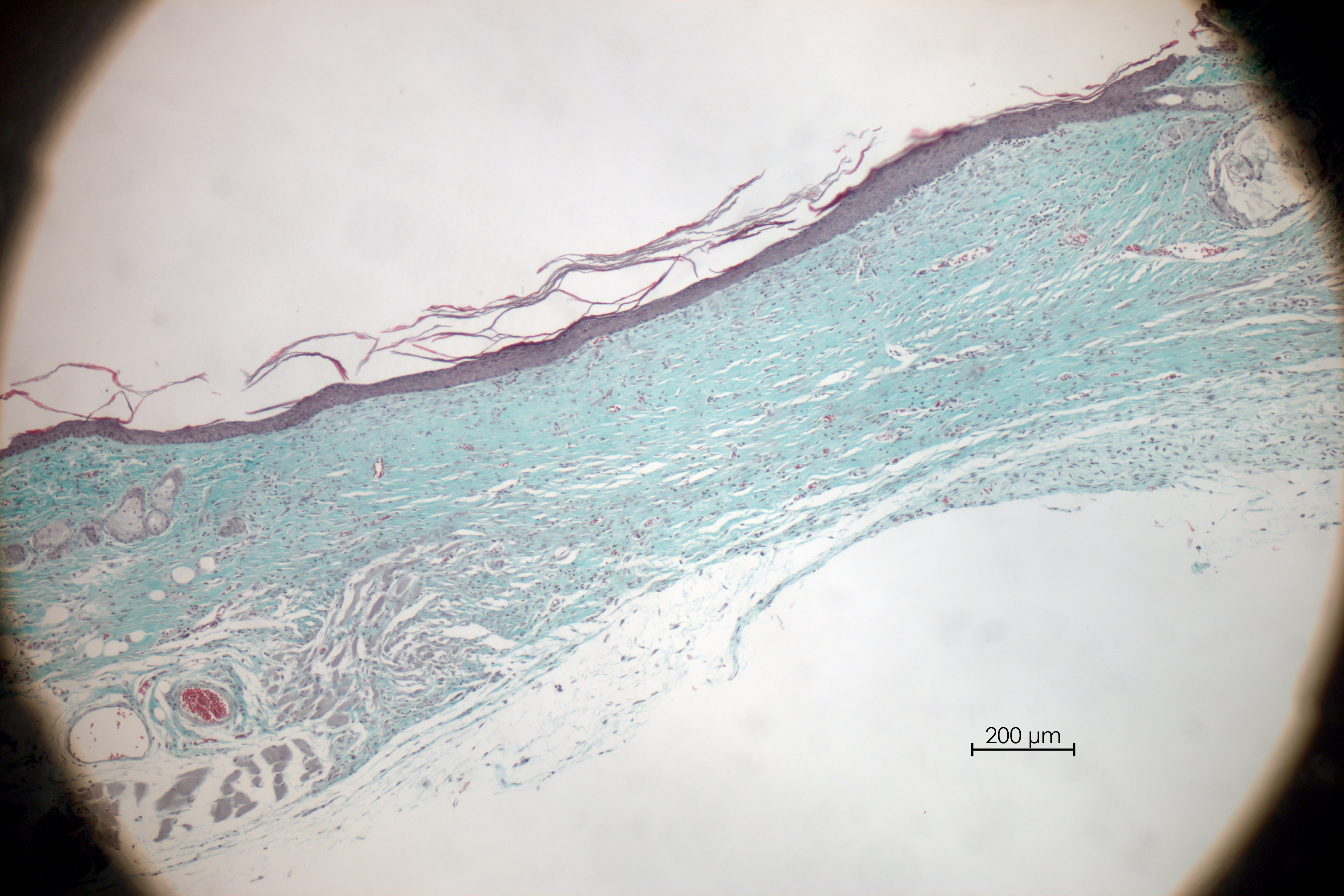

Supplement: Supplementary file 2 — Supporting file 2: adhm70839‐sup‐0002‐Complete Data.zip [file ADHM-15-0-s001.zip › Complete Data/Histology/Trichrome/CA-PLCL pristine - Trichrome.jpg]

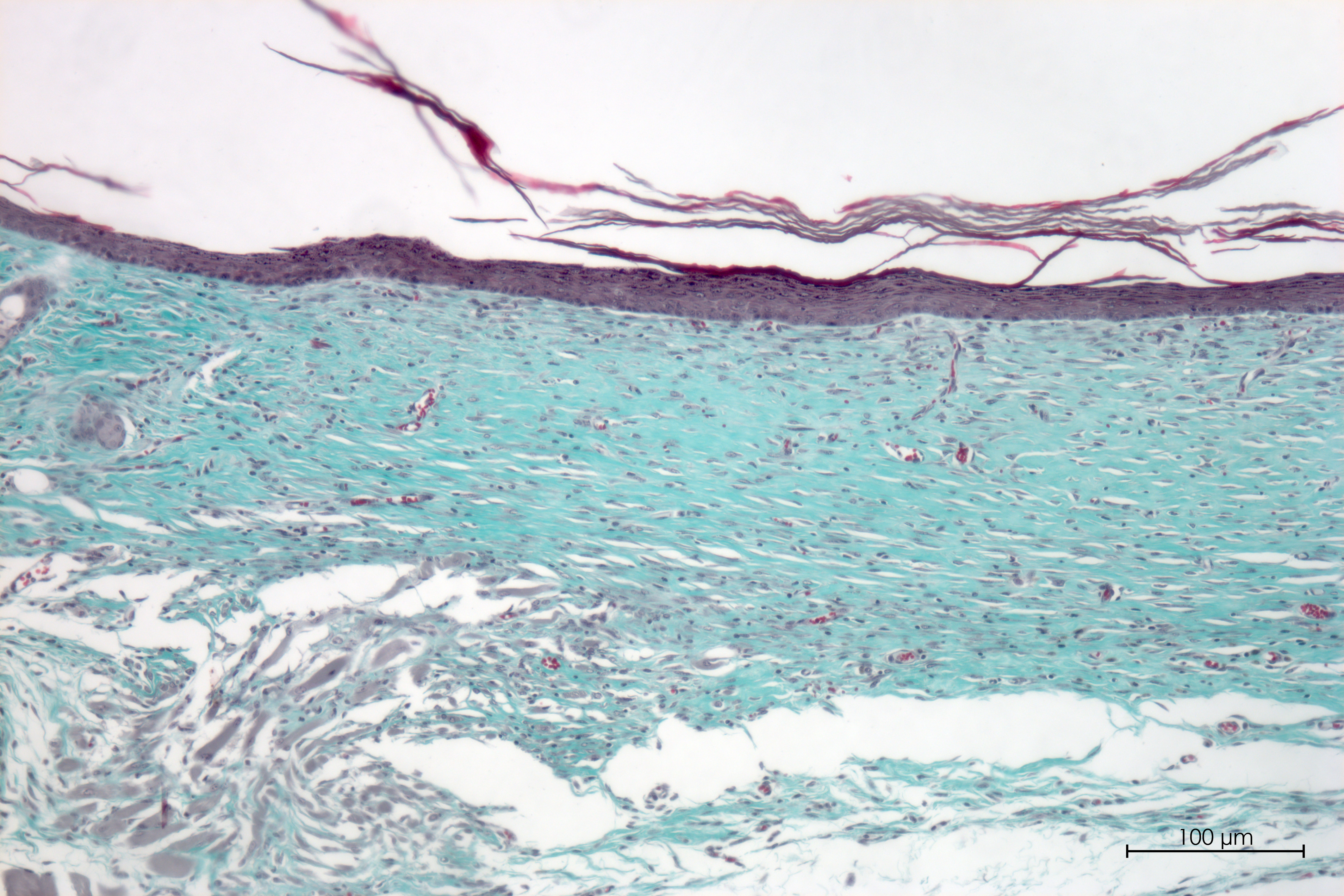

Supplement: Supplementary file 2 — Supporting file 2: adhm70839‐sup‐0002‐Complete Data.zip [file ADHM-15-0-s001.zip › Complete Data/Histology/Trichrome/CA-PLCL pristine Trichr.jpg]

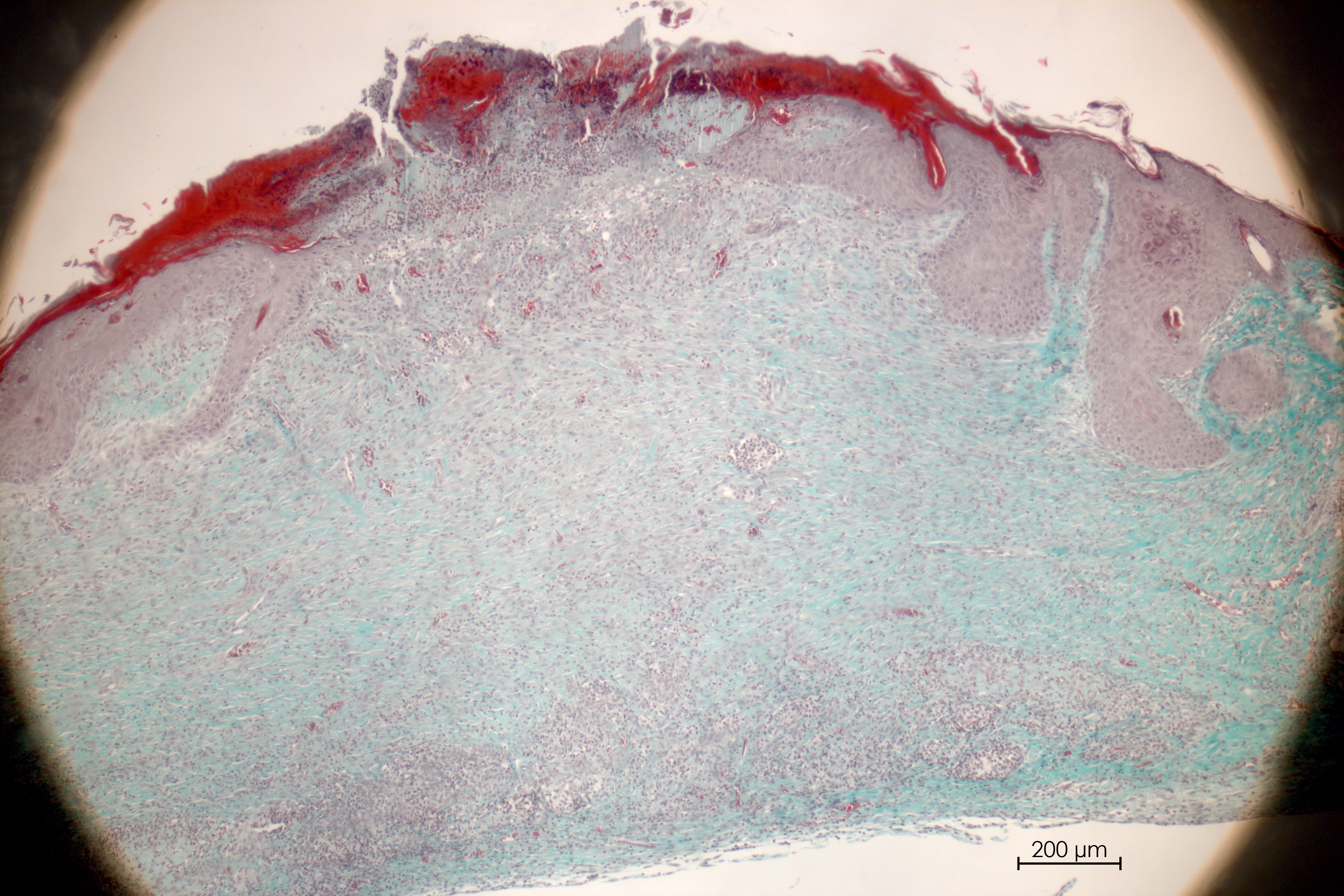

Supplement: Supplementary file 2 — Supporting file 2: adhm70839‐sup‐0002‐Complete Data.zip [file ADHM-15-0-s001.zip › Complete Data/Histology/Trichrome/Control-Trichrome.jpg]

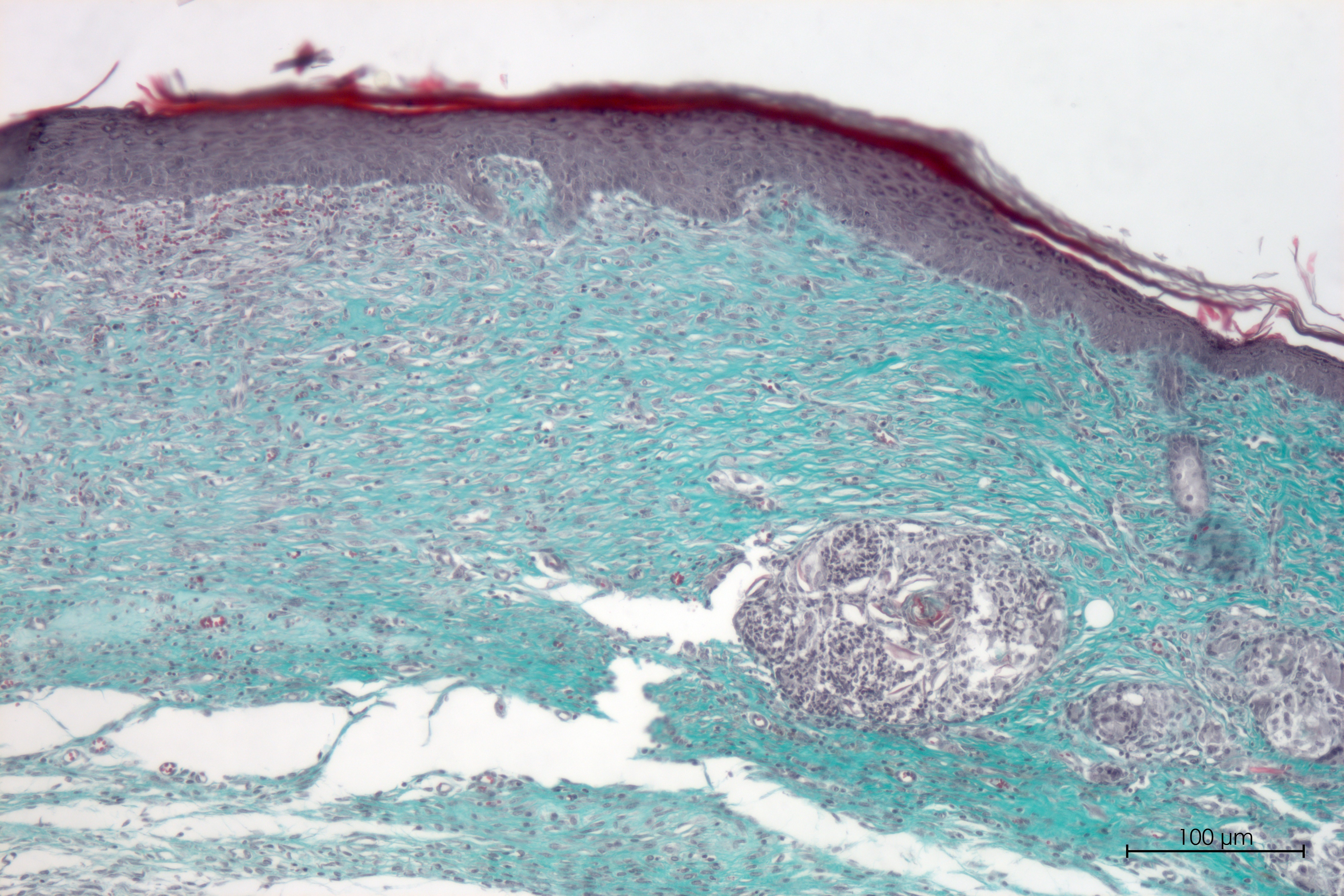

Supplement: Supplementary file 2 — Supporting file 2: adhm70839‐sup‐0002‐Complete Data.zip [file ADHM-15-0-s001.zip › Complete Data/Histology/Trichrome/PEO-PLCL loaded Trichr.jpg]

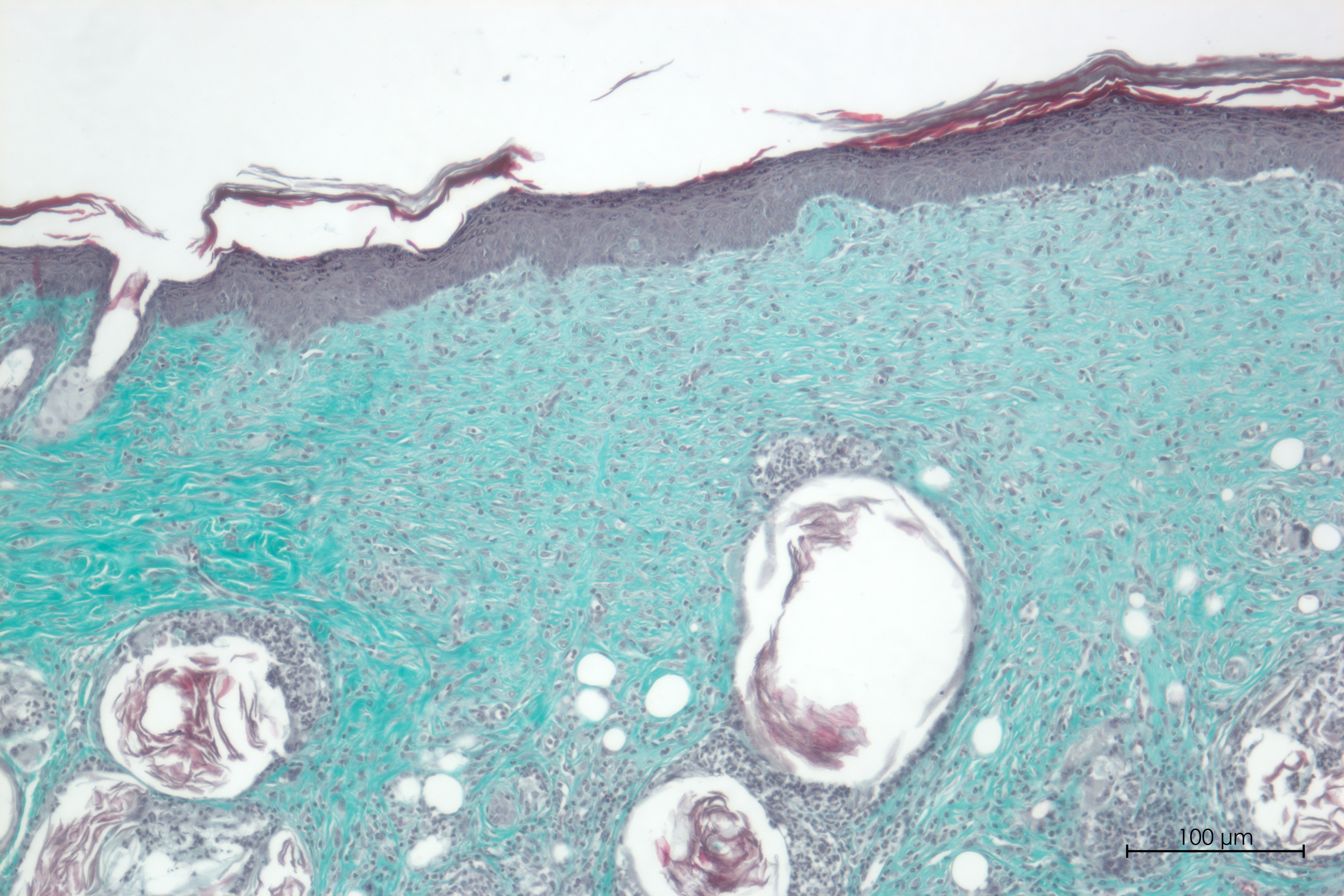

Supplement: Supplementary file 2 — Supporting file 2: adhm70839‐sup‐0002‐Complete Data.zip [file ADHM-15-0-s001.zip › Complete Data/Histology/Trichrome/PEO-PLCL pristine Trichr.jpg]

## Slide 1
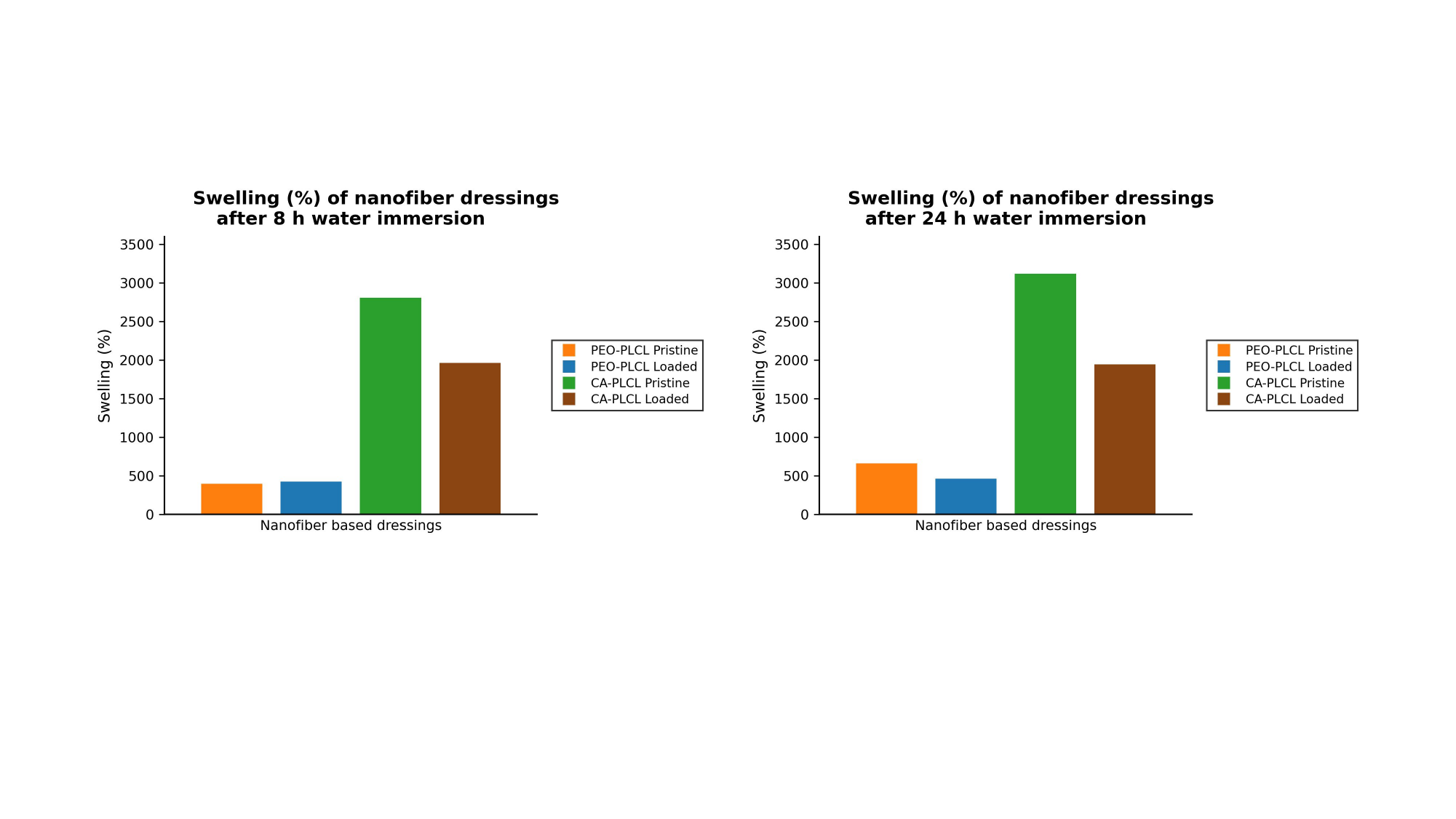

Supplement: Supplementary file 2 — Supporting file 2: adhm70839‐sup‐0002‐Complete Data.zip [file ADHM-15-0-s001.zip › Complete Data/Swelling-Layer thickness/Figure swelling.pptx]

## Slide 1
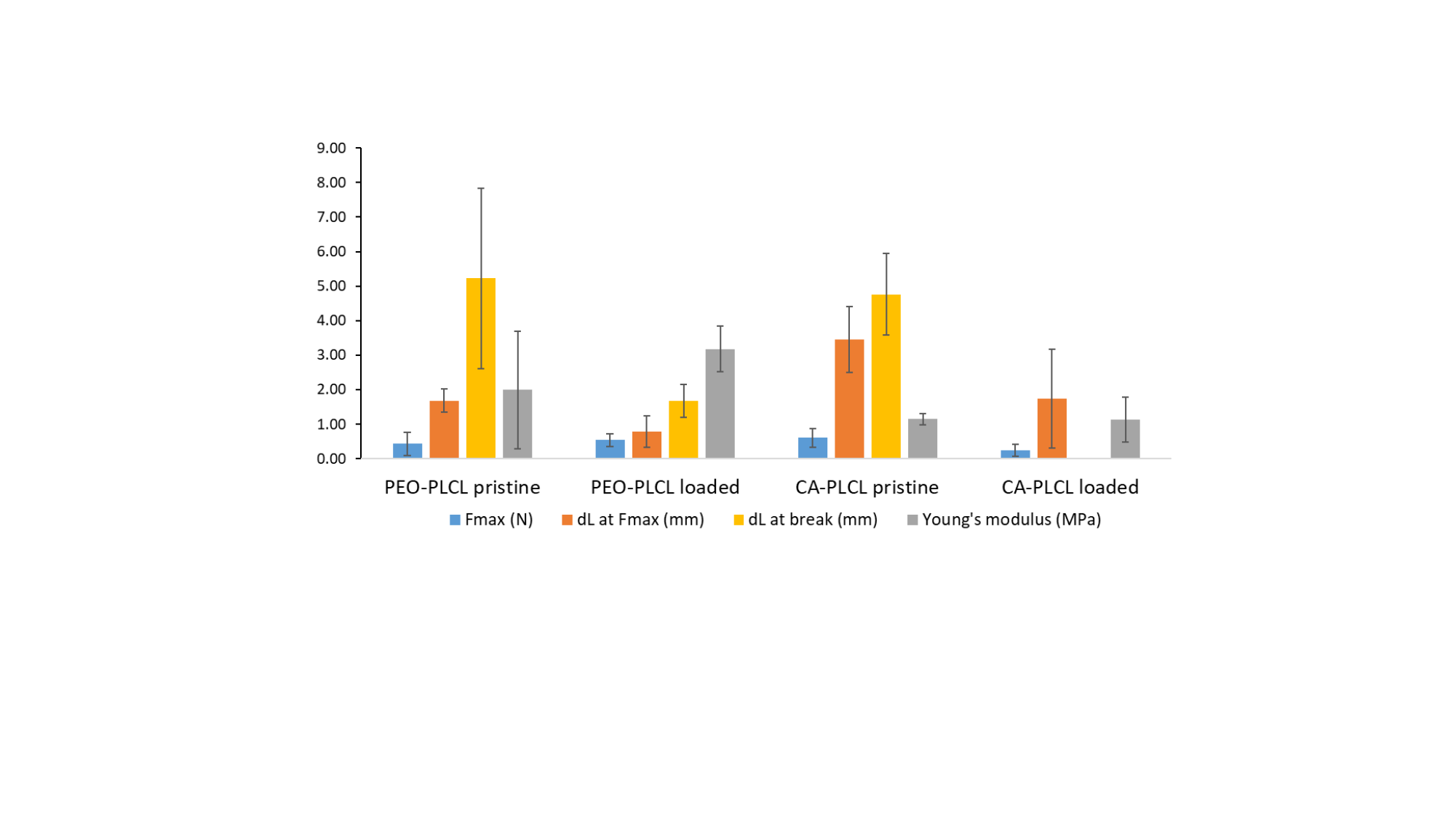

## Slide 2
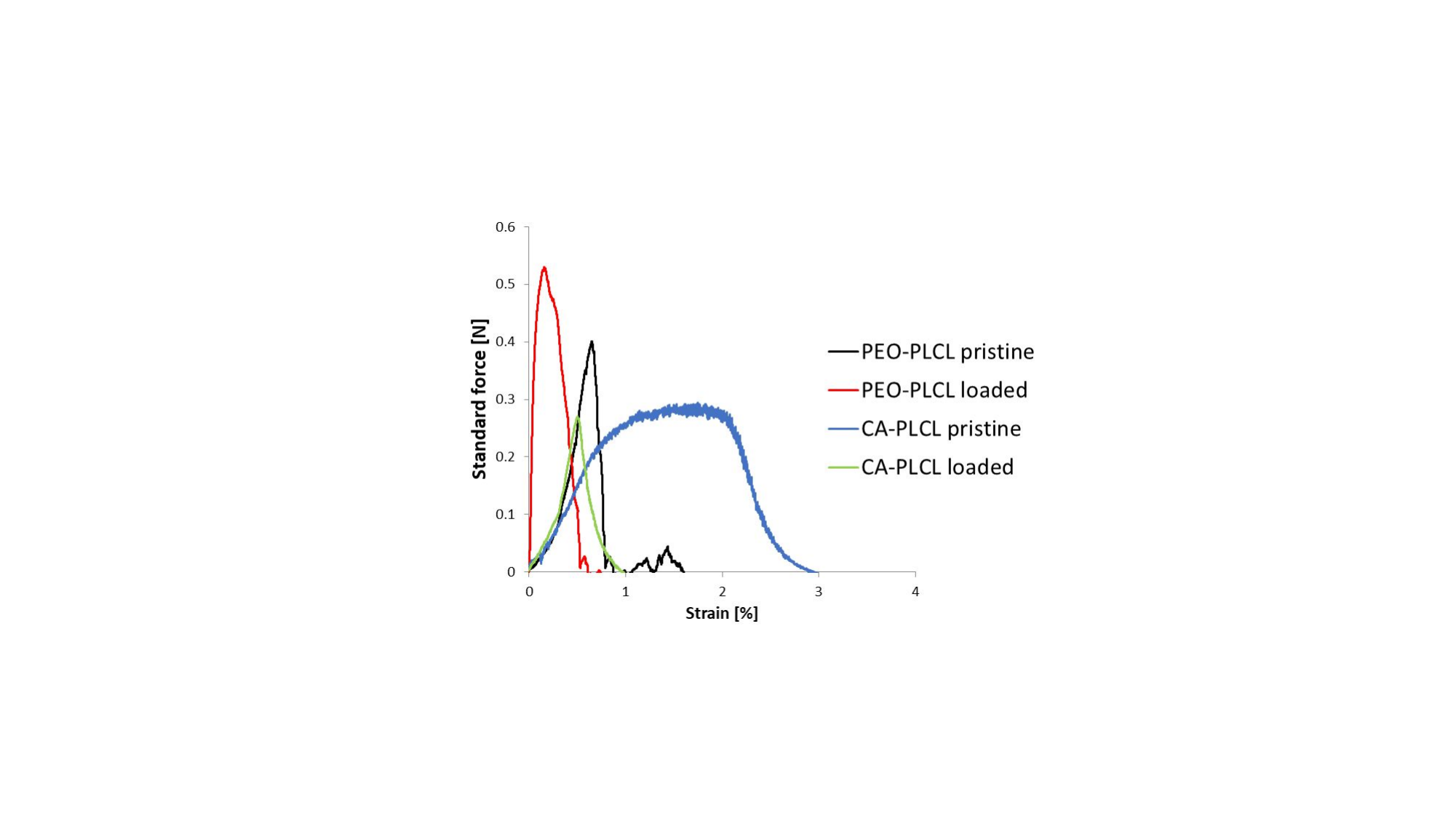

Supplement: Supplementary file 2 — Supporting file 2: adhm70839‐sup‐0002‐Complete Data.zip [file ADHM-15-0-s001.zip › Complete Data/Tensile strength - viscosity/Figure tensile.pptx]

## Slide 1
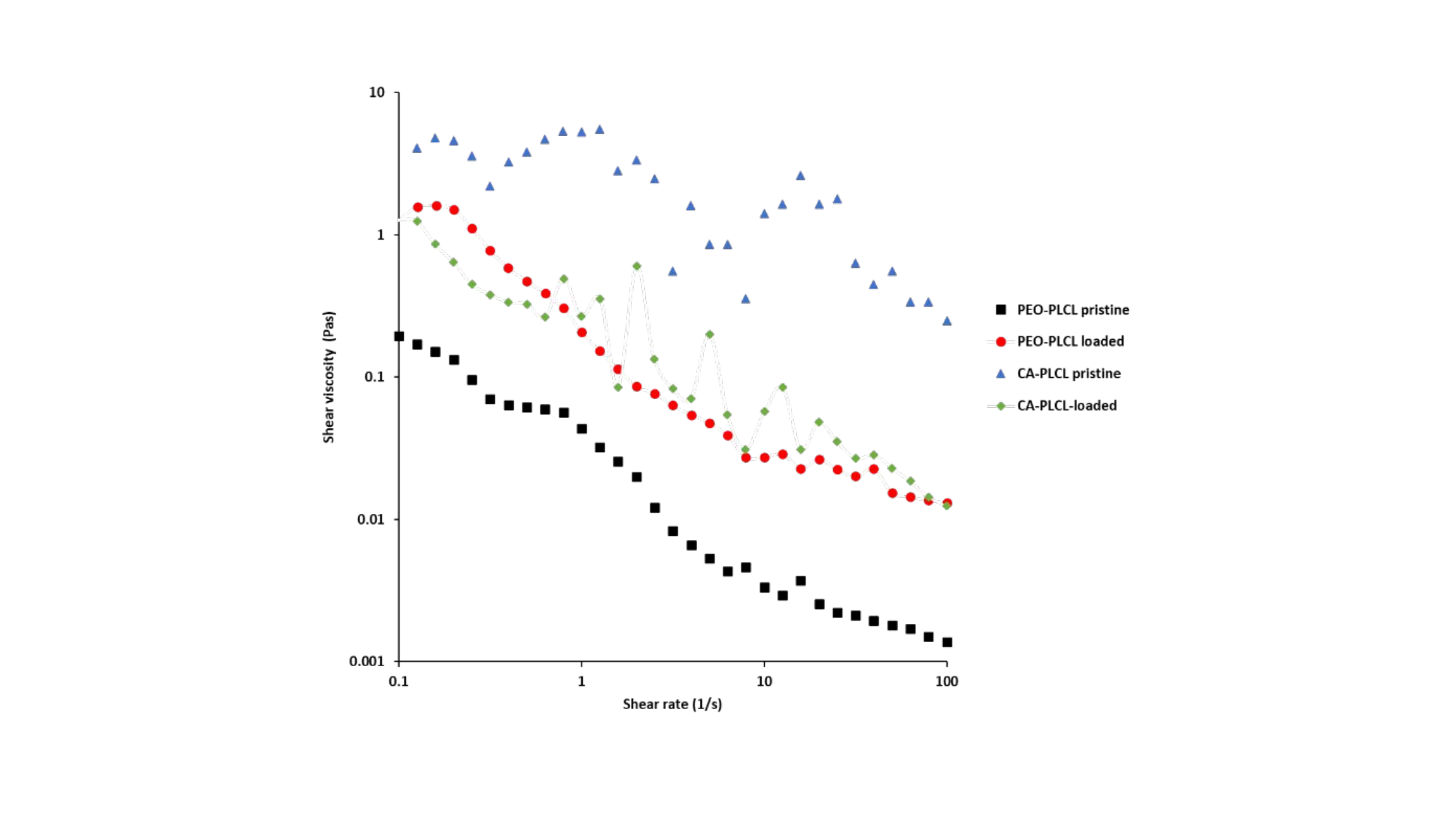

Supplement: Supplementary file 2 — Supporting file 2: adhm70839‐sup‐0002‐Complete Data.zip [file ADHM-15-0-s001.zip › Complete Data/Tensile strength - viscosity/Figure viscosity.pptx]

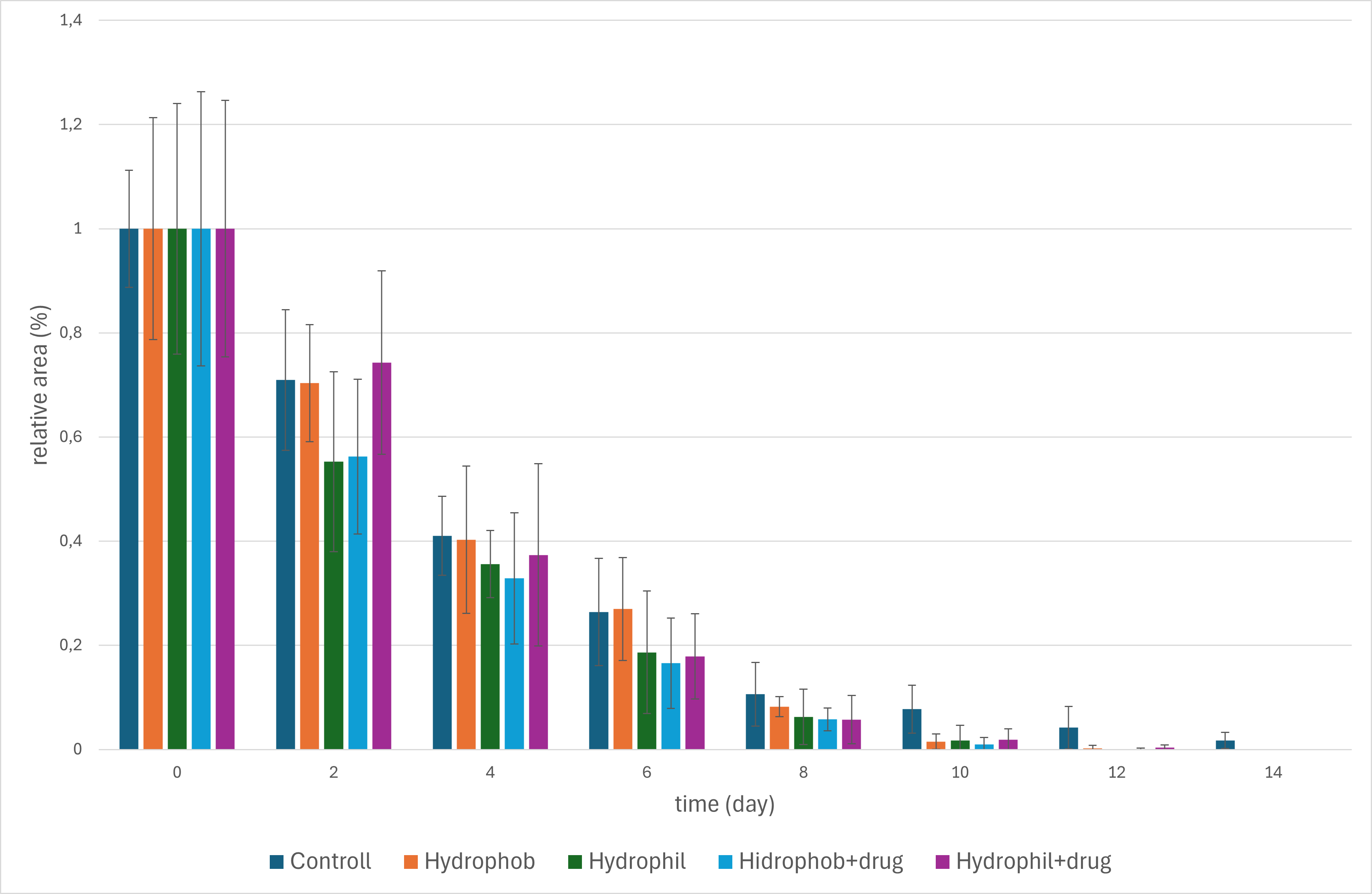

Supplement: Supplementary file 2 — Supporting file 2: adhm70839‐sup‐0002‐Complete Data.zip [file ADHM-15-0-s001.zip › Complete Data/Wound diameter - area/wh_area.png]

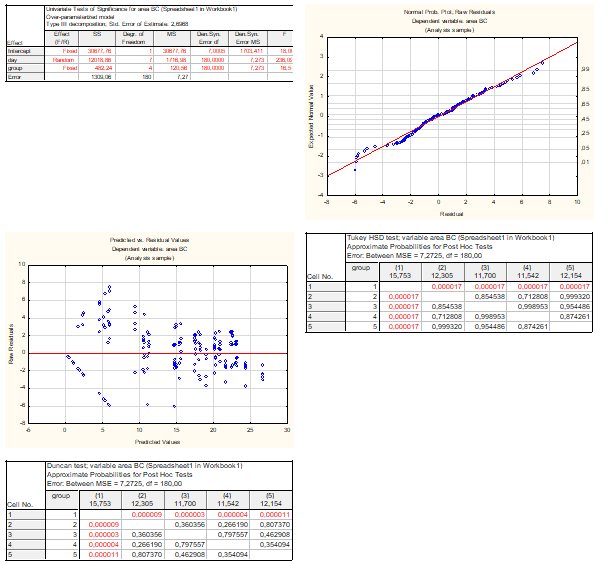

Supplement: Supplementary file 2 — Supporting file 2: adhm70839‐sup‐0002‐Complete Data.zip [file ADHM-15-0-s001.zip › Complete Data/Wound diameter - area/wh_area_stat.png]

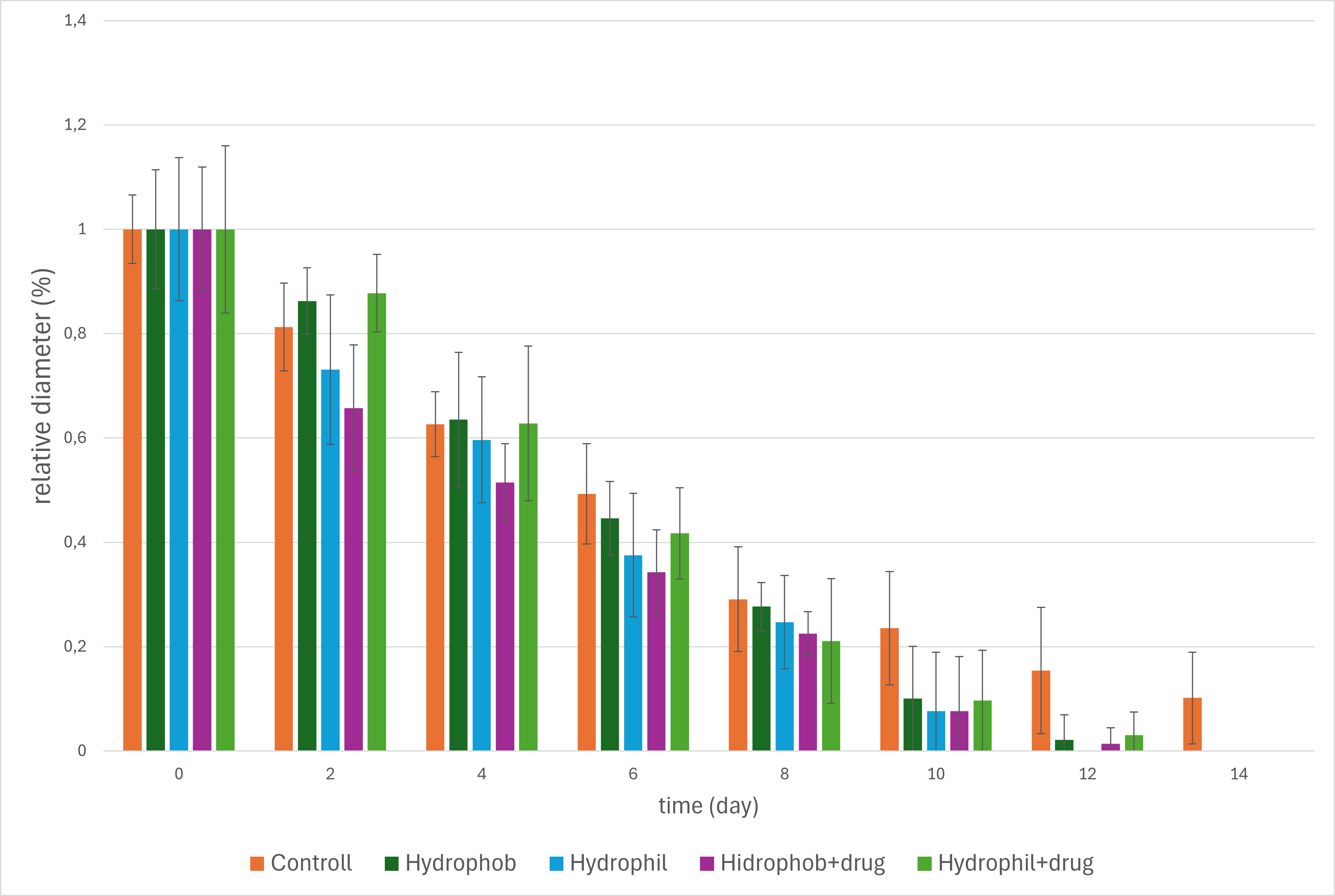

Supplement: Supplementary file 2 — Supporting file 2: adhm70839‐sup‐0002‐Complete Data.zip [file ADHM-15-0-s001.zip › Complete Data/Wound diameter - area/wh_diameter.png]

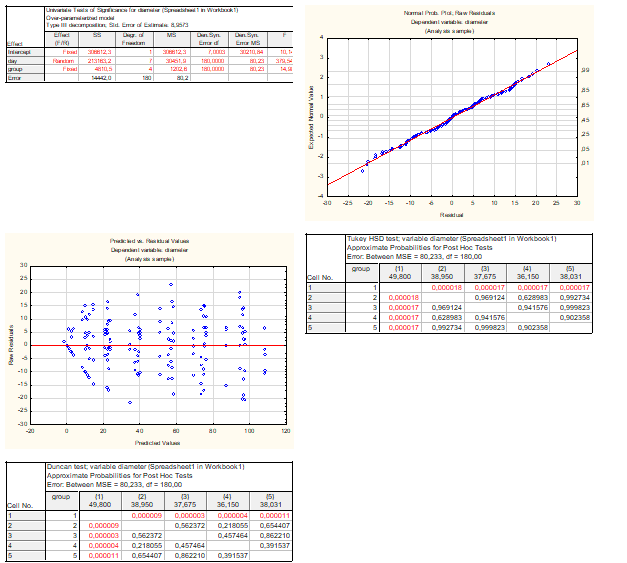

Supplement: Supplementary file 2 — Supporting file 2: adhm70839‐sup‐0002‐Complete Data.zip [file ADHM-15-0-s001.zip › Complete Data/Wound diameter - area/wh_diameter_stat.png]

## Slide 1
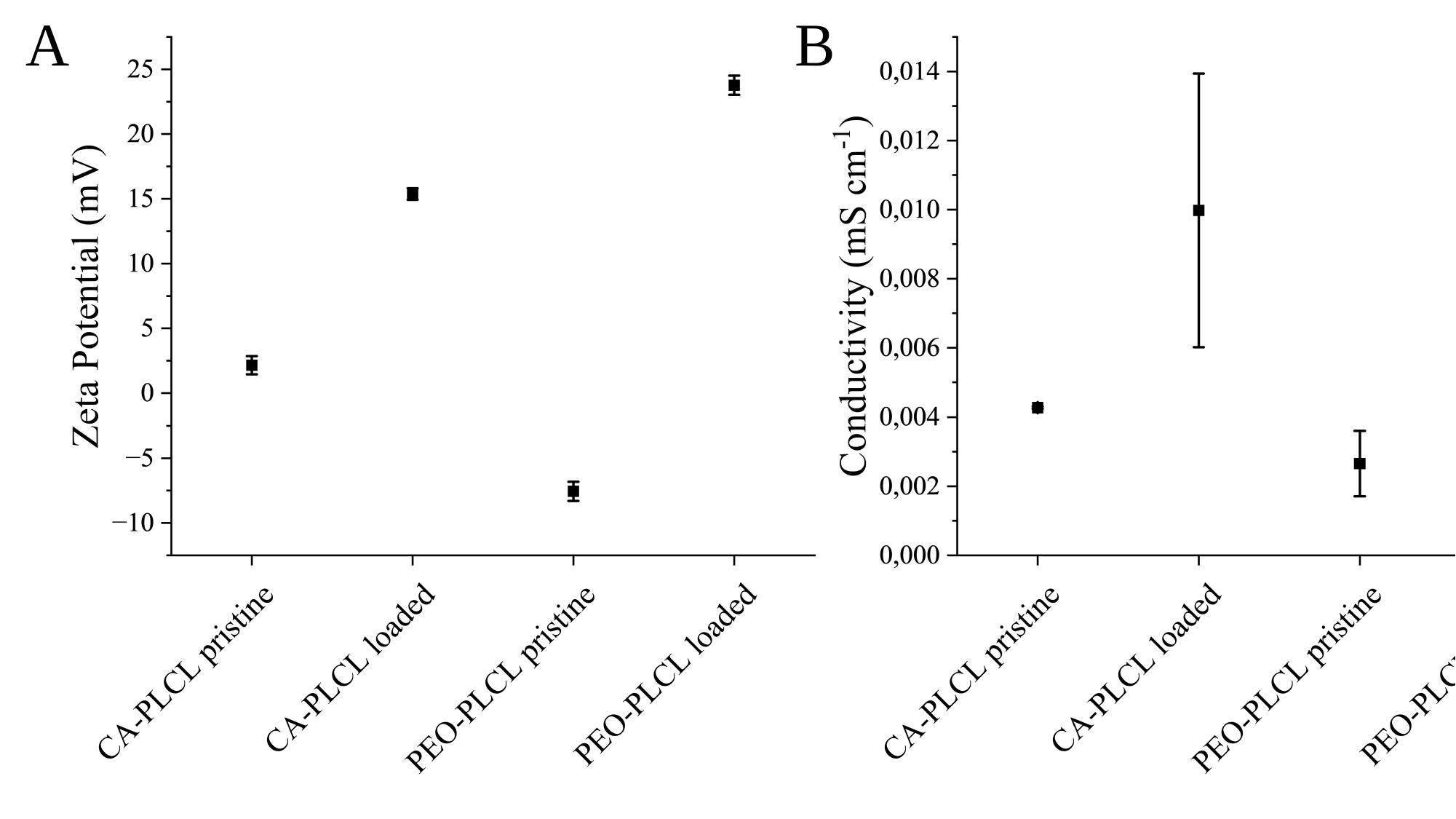

A
B
C

Supplement: Supplementary file 2 — Supporting file 2: adhm70839‐sup‐0002‐Complete Data.zip [file ADHM-15-0-s001.zip › Complete Data/Zeta Potential Data/Figure.pptx]
